# Whole genome sequencing provides comprehensive genetic testing in childhood B-cell acute lymphoblastic leukaemia

**DOI:** 10.1038/s41375-022-01806-8

**Published:** 2023-01-19

**Authors:** Sarra L. Ryan, John F. Peden, Zoya Kingsbury, Claire J. Schwab, Terena James, Petri Polonen, Martina Mijuskovic, Jenn Becq, Richard Yim, Ruth E. Cranston, Dale J. Hedges, Kathryn G. Roberts, Charles G. Mullighan, Ajay Vora, Lisa J. Russell, Robert Bain, Anthony V. Moorman, David R. Bentley, Christine J. Harrison, Mark T. Ross

**Affiliations:** 1 Translational and Clinical Research Institute, Newcastle University Centre for Cancer, Faculty of Medical Sciences, Newcastle upon Tyne, UK; 2 Illumina Cambridge Ltd., Granta Park, Great Abington, Cambridge, UK; 3 Department of Pathology, St. Jude Children’s Research Hospital, Memphis, TN USA; 4 Center for Applied Bioinformatics, St. Jude Children’s Research Hospital, Memphis, TN USA; 5 Department of Haematology, Great Ormond Street Hospital, London, UK; 6 Biosciences Institute, Newcastle University Centre for Cancer, Faculty of Medical Sciences, Newcastle upon Tyne, UK

**Keywords:** Cancer genomics, Cytogenetics

## Abstract

Childhood B-cell acute lymphoblastic leukaemia (B-ALL) is characterised by recurrent genetic abnormalities that drive risk-directed treatment strategies. Using current techniques, accurate detection of such aberrations can be challenging, due to the rapidly expanding list of key genetic abnormalities. Whole genome sequencing (WGS) has the potential to improve genetic testing, but requires comprehensive validation. We performed WGS on 210 childhood B-ALL samples annotated with clinical and genetic data. We devised a molecular classification system to subtype these patients based on identification of key genetic changes in tumour-normal and tumour-only analyses. This approach detected 294 subtype-defining genetic abnormalities in 96% (202/210) patients. Novel genetic variants, including fusions involving genes in the MAP kinase pathway, were identified. WGS results were concordant with standard-of-care methods and whole transcriptome sequencing (WTS). We expanded the catalogue of genetic profiles that reliably classify *PAX5*alt and *ETV6::RUNX1*-like subtypes. Our novel bioinformatic pipeline improved detection of *DUX4* rearrangements (*DUX4*-r): a good-risk B-ALL subtype with high survival rates. Overall, we have validated that WGS provides a standalone, reliable genetic test to detect all subtype-defining genetic abnormalities in B-ALL, accurately classifying patients for the risk-directed treatment stratification, while simultaneously performing as a research tool to identify novel disease biomarkers.

## Introduction

Childhood and adolescent B-cell acute lymphoblastic leukaemia (B-ALL) is one of the success stories of modern medicine, with survival rates exceeding 90% [1]. Risk stratification for treatment within contemporary clinical trials has contributed significantly to this achievement, through classification into risk groups based on genetic subtyping and minimal residual disease (MRD) assessment [2]. Approximately 70% of childhood B-ALL are currently routinely characterised by established cytogenetic abnormalities, associated with good or poor outcomes [3]. The remaining 30% of patients, lacking established chromosomal aberrations and termed “B-other-ALL”, were collectively assigned to the intermediate risk group. More recently, genomic approaches have identified new genetic subtypes among B-other-ALL, including *DUX4*-rearranged (*DUX4-*r, mostly *IGH::DUX4*), ABL-class fusions, and *MEF2D*-rearranged (*MEF2D*-r), which have been associated with specific clinical characteristics and differing outcomes (Table 1) [4–10]. Thus, their accurate detection is increasingly important for assignment to the most appropriate therapy, as now well-established for ABL-class fusions and treatment with tyrosine kinase inhibitor (TKI) therapy [11, 12].

**Table 1:**
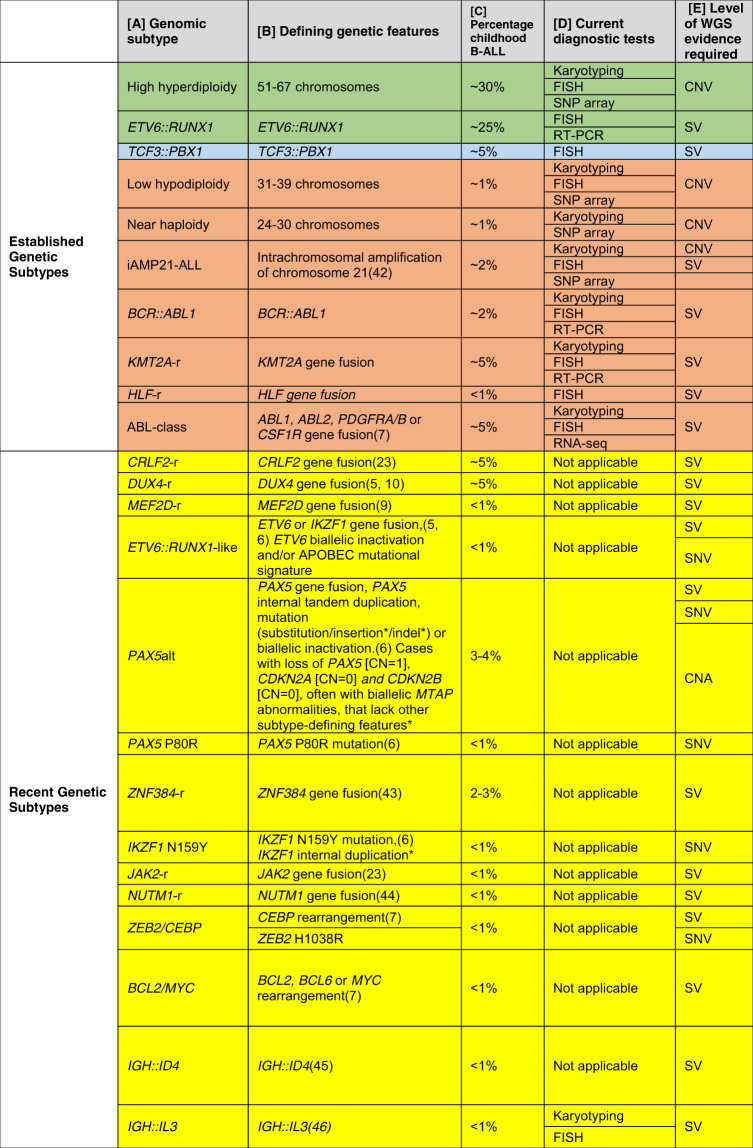
Summary of molecular features used for B-ALL genetic subtyping.

Defining genetic features [B] for each subtype [A] are based on previous studies. *Associated genomic features characteristic of certain subtypes were identified from 85 B-ALL patients with matched WGS and WTS data. The type of genetic abnormality (CNA, SV, SNV) required for subtyping is shown [E]. Diagnostic genetic tests recommended for the detection of clinically relevant genetic abnormalities are provided [D] [13, 14]. Subtypes associated with a favourable (highlighted green), intermediate (highlighted blue) and poor (highlighted orange) risk are shown. Screening is not yet routine for the recent genetic subtypes (highlighted yellow), thus, specific tests have not been recommended (‘not applicable’ in [D]).

A range of standard-of-care techniques are currently used to detect clinically-relevant abnormalities in B-ALL, including karyotyping, fluorescence in situ hybridisation (FISH) and SNP arrays [13, 14]. As the list of subtype-defining genetic abnormalities has grown, multiple tests are often required. In addition, it has been difficult to achieve a single test for some important abnormalities, such as *DUX4*-r, which defines a distinct subtype of B-ALL with a good prognosis [6, 7]. Detection of this rearrangement is challenging due to the repetitive and variable sequences involved [15]. Molecular schema for B-ALL subtyping using whole transcriptome sequencing (WTS) have recently been described, but they require large reference datasets as they are focused on global expression profiles [7, 16]. These challenges, together with the lack of recurrent genetic abnormalities in around 10% of patients [17], drive the need for enhanced genetic diagnostic tools in B-ALL.

Genetic testing using whole genome sequencing (WGS) is becoming increasingly feasible, as many of the analytical demands are being addressed [18, 19]. Indeed, some national healthcare providers, such as the NHS Genomic Medicine Service and Genomic Medicine, Sweden, are offering WGS as a genetic test for haematological and other paediatric malignancies [20]. To date, WGS has been used to explore the genomic landscape of B-ALL subtyped by WTS [17]. A DNA-based molecular schema for subtyping with comprehensive validation of WGS as a standalone diagnostic tool in B-ALL has not been performed. In this study of a large cohort of genetically and clinically well-annotated childhood and adolescent B-ALL, we have produced such a schema for accurate subtyping by WGS, which has clearly demonstrated the future role of WGS for improved genetic risk-directed stratification compared to previous standard-of-care approaches.

## Materials and methods

### Patient information

Diagnostic bone marrow samples from 210 childhood B-ALL were included in this study, and divided into two cohorts: (1) known cytogenetic abnormalities of clinical significance (*n* = 38) (patients 1–38); (2) no established cytogenetic abnormalities detected by standard-of-care methods and treated on the UK childhood ALL treatment trial, UKALL2003 (defined as “B-other-ALL” at the time of the trial, *n* = 172) (patients 39–210, Supplementary Table 1, Supplementary Information) [21, 22]. All patients were diagnosed using standard morphological and immunophenotyping methods. Leukaemic blast count at diagnosis was ≥70% in 92% (163/177) of patients with information available. Post-treatment bone marrow samples were used as matched germline controls for 208 patients (Supplementary Table 1, Supplementary Information).

### Whole genome sequencing (WGS)

WGS was performed on 210 diagnostic (mean sequencing depth of 76×) and 208 matched remission (mean sequencing depth of 38×) DNA samples, as described in Supplementary Information.

Reads were aligned to Human Reference genome version 38 (GRCh38), and germline and somatic variants were identified, as described in Supplementary Information. We considered structural variants (SV) or copy number variants/aberrations (CNV/CNA) that were located within or surrounding (≤10 kb) a gene, as well as single nucleotide variants (SNVs) and indels that were located within the coding sequence of a gene. Tumour-only (T-only) analysis was performed on all patients with established genetic abnormalities of clinical significance (*n* = 38, cohort 1) and diagnostic samples without a matched germline (*n* = 2), as described in Supplementary Information.

Individual B-ALL samples (*n* = 210) were classified into subtypes based on the detection of specific genetic features (Table 1, Supplementary Information). In cases with genetic alterations characteristic of ≥2 subtypes, the primary subtype was assigned as the one with the highest supporting read count, except for *DUX4*-r cases, due to difficulties in mapping reads to these regions. Cases lacking obvious defining features were classified as ‘other’.

### Detection of *DUX4-*rearrangements by WGS

A customised T-only pipeline was developed for the detection of *IGH::DUX4* spanning reads in our B-ALL samples (*n* = 210). Available matched germline samples (*n* = 208) were used to determine the baseline level of spanning reads expected to be false positive alignments. Spanning read pairs from all samples with >10 spanning reads per billion (SRPB) were locally assembled to generate contigs and scaffolds used for *IGH::DUX4*. Cases of *DUX4*-r with other (non-*IGH*) partner genes were identified, as described in Supplementary Information.

### Detection of clinically relevant genetic abnormalities by WGS

Cytogenetics, FISH and Multiplex Ligation-dependent Probe Amplification (MLPA) (MRC Holland, The Netherlands) were performed, as previously described [23–25]. Abnormalities detected by these methods are provided in Supplementary Table 2. Copy number data for nine genes/regions targeted in the SALSA MLPA P335 (*CDKN2A/B, PAX5, IKZF1, BTG1, EBF1, RB1, ETV6*, PAR1 region) and P327 (*ERG*) kits were included. Detection of risk-stratifying genetic abnormalities by WGS required these key features: (1) ploidy and focal CNA required CNA and/or SV information and (2) gene fusion required evidence of a SV [26, 27].

### Cross validation using whole transcriptome sequencing (WTS)

WTS was performed on RNA samples, extracted from diagnostic bone marrow using the RNeasy Extraction kit (Qiagen, Manchester, UK), from 85 patients within the same B-ALL cohort. Sequencing reads were processed and aligned to Human Reference Genome GRCh38. Molecular classification and subtyping was performed, as previously described (Supplementary Information) [6, 7, 28].

## Results

### Robust detection of established cytogenetic abnormalities by WGS

We have demonstrated that WGS can reliably detect the important risk-stratifying genetic abnormalities in B-ALL by investigating the 38 samples with known chromosomal abnormalities (cohort 1) (Supplementary Fig. 1A). The automated DRAGEN T-only pipeline called 37/38 of the primary genetic abnormalities without analysis of the associated germline sample, while automated T-N analysis identified 34/38 of them. Using either approach, the patterns of whole chromosome gain or loss in subtypes associated with aneuploidy (high hyperdiploidy, low hypodiploidy, near-haploidy) and whole chromosome copy number-neutral loss-of-heterozygosity (CN-LOH), identifying masked near-haploidy/low hypodiploidy, were consistent with FISH and cytogenetic analyses (Supplementary Table 2). In addition, the complex genomic profile of ALL with intrachromosomal amplification of chromosome 21 (iAMP21-ALL, *n* = 3), and in-frame gene fusions of *KMT2A*-r (*n* = 8), *ETV6::RUNX1* (*n* = 5) and *TCF3::PBX1* (*n* = 3), were consistently identified using T-N and T-only approaches.

T-N analysis failed to identify the expected fusion gene in four patients: one of three *BCR::ABL1* and all three *EBF1::PDGFRB* (ABL-class subtype) cases (Supplementary Table 2). Two factors affected their detection by the T-N automated pipeline: (1) *high levels of residual leukaemic blasts (high MRD) in the germline sample*. Aligned sequencing reads showed evidence of the fusion gene in both leukaemia and matched germline samples in all four patients (Supplementary Fig. 1B–E). Although remission bone marrow was easily accessible for use as a matched germline control in this study, other germline samples, including skin biopsies, hair or nail extracts, may be better options. Crucially, automated T-only analysis identified *EBF1::PDGFRB* in all three patients. (2) *Complex rearrangement near the breakpoint*. The *BCR::ABL1* rearrangement showed a second rearrangement (duplication) visible in the aligned reads close (418 bp) to the *BCR::ABL1* breakpoint, which had been called as a separate event by automated analysis (Supplementary Fig. 2). Interestingly, this same duplication close to the *BCR::ABL1* breakpoint was found in a second *BCR::ABL1* patient, which may indicate a recurring event. Importantly, all 38 risk-stratifying genetic abnormalities were successfully detected by WGS.

### Molecular classification and subtyping of B-other-ALL by WGS

Next, we investigated the 172 cases in cohort 2 by WGS T-N analysis. Reinforcing the accuracy of WGS, 19 cases harboured established chromosomal abnormalities, which were previously undetected due to limited material and incomplete standard-of-care testing at the time of diagnosis: high hyperdiploidy (*n* = 5), iAMP21-ALL (*n* = 3), *ETV6*::*RUNX1* (*n* = 1), *TCF3*::*PBX1* (*n* = 8), *TCF3*::*HLF* (*n* = 1), low hypodiploidy (*n* = 1) (Supplementary Table 1, Supplementary Information, Fig. 1). The eight *TCF3*::*PBX1* and one *TCF3*::*HLF* cases showed normal/undefined karyotypes and *TCF3* FISH had not been performed. Among the remaining 153 cases, we were able to characterise 145 patients with cytogenetically-cryptic, subtype-defining genetic abnormalities (Supplementary Table 3, Fig. 2). The predominant subtype was *DUX4*-r (*n* = 59), followed by *PAX5*alt (n = 29), *ZNF384*-r (*n* = 12) and *ETV6::RUNX1*-like (*n* = 12). An additional *DUX4*-r, in association with iAMP21-ALL, was identified within cohort 1 (20724), bringing the total *DUX4*-r cases to 60. Two subtype-defining genetic abnormalities were identified in the same patient sample in eight cases, as indicated in Supplementary Table 3. Although the clinical significance of such co-existing abnormalities requires further assessment, their detection and estimate of subclonality was facilitated by WGS.

**Fig. 1:**
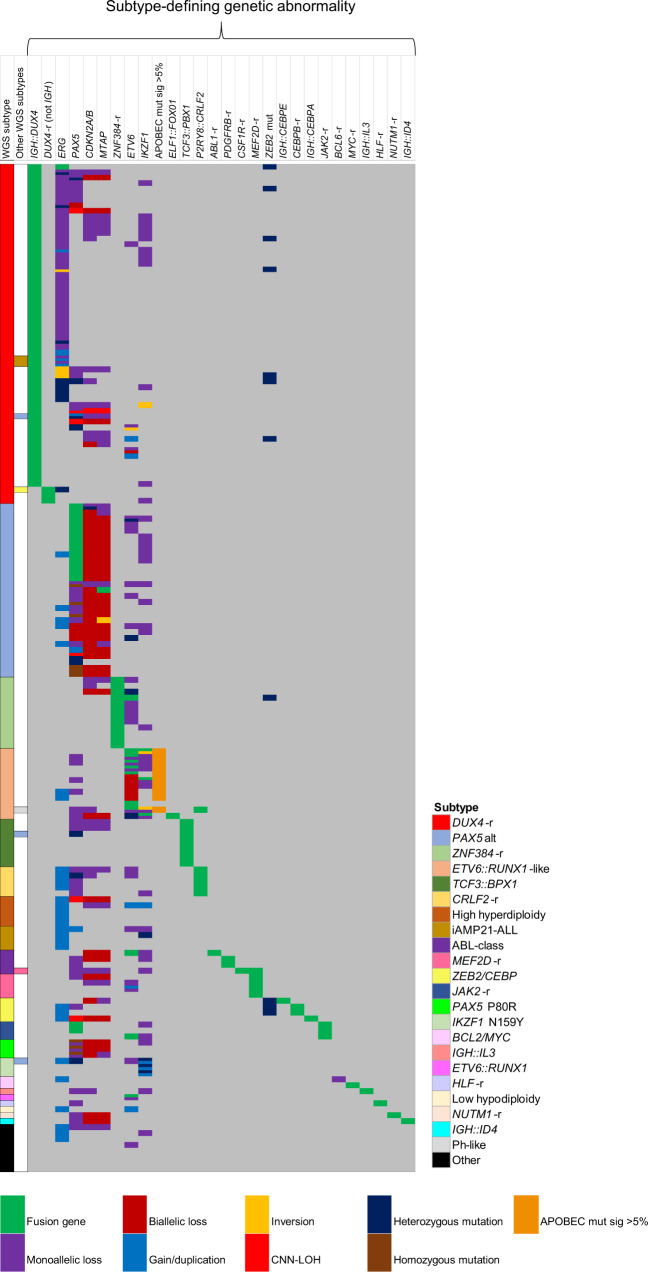
The landscape of subtype-defining genetic alterations. Oncoplot showing the subtype definition of each case and the associated subtype-defining genetic abnormalities observed by WGS. Colours define the subtype of each patient and the type of rearrangement for each genetic abnormality.

**Fig. 2:**
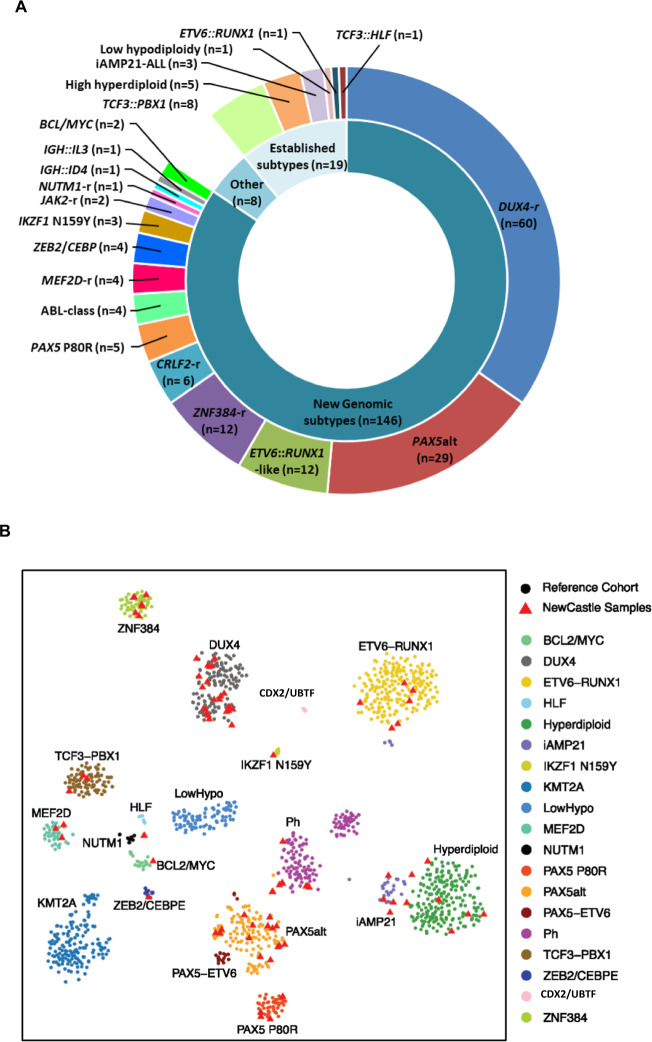
Molecular subtyping of B-other-ALL. **A** Genetic subtypes as defined by WGS of 173 B-ALL patients, including 172 B-other-ALL patients (cohort 2) and one patient with iAMP21-ALL (cohort 1) in which a *DUX4*-r was observed. Eight patients had no subtype-defining genetic abnormalities (termed ‘other’). **B** t-distributed stochastic neighbour embedding (tSNE) plot of 85 B-other-ALL patients from this study (red triangles) and 1452 B-ALL patients from our previous study, demonstrating the subtype groupings (colour coded) of each patient based on WTS data [7]. This analysis validated the identification of recent subtype-defining genetic abnormalities that were identified within the WGS data.

**Fig. 3:**
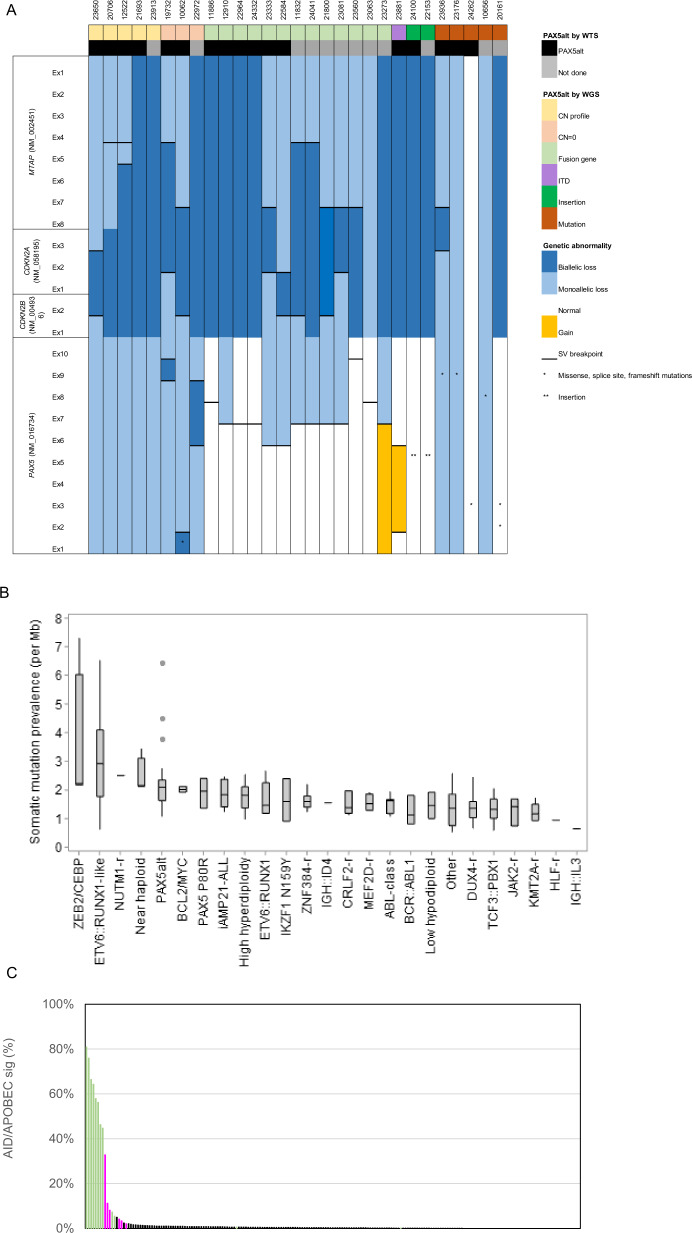
Novel subtype-defining abnormalities discovered by WGS. **A** Genetic abnormalities (CNA, SV, missense/frameshift/splice site mutations and small insertions (<500 bp)) involving individual exons of *PAX5*, *CDKN2A*, *CDKN2B* and *MTAP* in *PAX5*alt cases (*n* = 29). ‘CN profile’ describes a group of *PAX5*alt cases identified by WGS with *PAX5* loss, biallelic *CDKN2A* and *CDKN2B* loss, often with *MTAP* abnormalities. *PAX5*alt subtyping was validated in all patients with matched WTS data (*n* = 17). **B**, **C** The mutational load in *ETV6::RUNX1*-like patients is shown to be elevated (median 2.91, range 0.63–6.5) (**B**), andthe AID/APOBEC family of cytidine deaminases represents >5% of the mutational signature profile in 10/12 *ETV6::RUNX1*-like cases (green) (**C**). Enrichment of the AID/APOBEC mutational signature is also evident in *ETV6::RUNX1* patients (pink), as previously reported [30].

Among the subtypes characterised by gene rearrangements, 55 different fusion genes were identified by WGS (Supplementary Table 4). These included three different partner genes of *DUX4*: *IGH* (*n* = 57) and novel partners *MYB* (*n* = 1, #23445) and *DNTT* (*n* = 1, #11148). One partner gene (#22355) remains unidentified, as discussed in Supplementary Information. *PAX5* and *ETV6* rearrangements were the most variable in relation to breakpoint and partner genes involved (Supplementary Fig. 3). Fusion genes involving *PAX5* and *ETV6* are characteristic of *PAX5*alt and *ETV6::RUNX1*-like ALL, respectively, which are often genetically complex (Supplementary Table 5) and associated with a range of underlying genetic abnormalities that drive global transcriptome profiles as defined by WTS [6, 29]. Here, WGS identified subtype-defining genetic abnormalities in all *PAX5*alt and *ETV6::RUNX1*-like cases, including a number of new aberrations. For example, a large insertion (>250 bp) involving exon 5 of *PAX5* was observed in two *PAX5*alt cases, and five cases of *PAX5*alt were found to share a common profile of monoallelic *PAX5* and biallelic *CDKN2A*/*B* losses, often with biallelic *MTAP* abnormalities (4/5 patients). Importantly, these cases lacked other subtype-defining genetic abnormalities and were validated as *PAX5*alt in those patients with matched WTS data (Fig. 3A). Separately, the mutational signature associated with the AID/APOBEC family of cytidine deaminases and a higher mutational load was demonstrated to be a robust associated genetic abnormality in *ETV6::RUNX1*-like patients, as previously reported (Fig. 3B, C) [17, 30]. Notably, the co-existence of an internal tandem duplication of *IKZF1* (consistently involving exon 5) was observed in all *IKZF1* N159Y patients in this study (3/3). The wide range of genetic profiles detected within B-other-ALL by WGS emphasises the challenges in their detection using standard-of-care techniques.

### Characterisation of ‘other’ cases by WGS

**Fig. 4:**
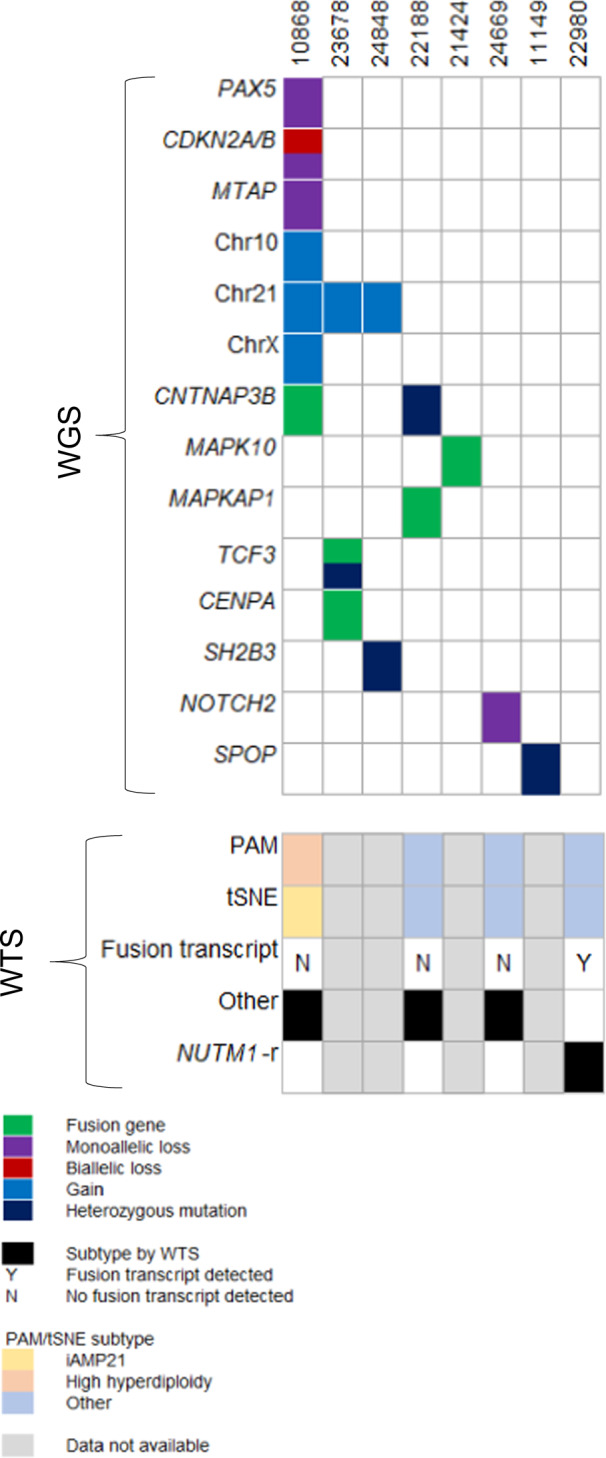
Key genetic abnormalities in eight “other” patients subtyped by WGS. The oncoplot provides details of genetic abnormalities that were clonal, recurrent or within ALL-associated genes detected by WGS. The primary subtype (black) defined by WTS is shown for four patients with matched WTS data. The subtype definition of each case based on Prediction Analysis of Microarrays (PAM) or two-dimensional t-distributed stochastic neighbour embedding (tSNE) analyses is shown. The presence of a subtype-defining fusion transcript in each sample is given; apart from patient 22980 with a *ZNF618-NUTM1* fusion by WTS only. No fusion transcript was detected in the remaining patients.

Although no subtype-defining genetic abnormalities were observed in eight patients (Fig. 4**)**, seven of them harboured genetic abnormalities that were clonal, recurrent in our cohort and/or located within known ALL-associated genes. A *SH2B3* mutation in combination with gain of chromosome 21 was identified in one patient (*n* = 1, #10868), an association that we have previously reported [31]. Two patients had abnormalities of *CNTNAP3B*, a gene previously reported to be rearranged in infant *KMT2A*-r ALL cases: [32] a *CNTNAP3B::C20orf203* fusion (#10868) and a missense mutation (Gly520Ala) (#22188). Multiple clonal rearrangements involving *TCF3* and novel partner genes were identified in one patient (#23678), and a 1.5 MB (chr1:119983209-121429772) deletion was identified that targeted up to eight genes, including exons 1–6 of *NOTCH2*, in patient #24669. Previously unreported recurrent or clonal rearrangements involved genes within the mitogen-activated protein kinase (MAPK) pathway: *UBA6_AS1::MAPK10* (*n* = 1, #21424) and *RRAGB::MAPKAP1* (*n* = 1, #22188) (Supplementary Table 4).

We had matched WTS data for four of the above eight patients (Fig. 4). The results in relation to subtype definition by WGS concurred in three patients; the fourth case (22980) was classified as *NUTM1*-r by WTS alone. By WGS, no recurrent or clonal abnormalities were detected, even from inspection of aligned reads over *NUTM1*. However, it had a lower blast count (56% compared to an average blast count of 89% for the entire cohort), potentially explaining the inability to detect *NUTM1-r*, or any other clonal genetic feature, by WGS. As lower counts may hinder accurate detection of abnormalities, enrichment of blasts prior to DNA extraction or increased sequencing coverage in samples with <70% blasts, may be beneficial.

### Improved detection and characterisation of *DUX4* rearrangements

**Fig. 5:**
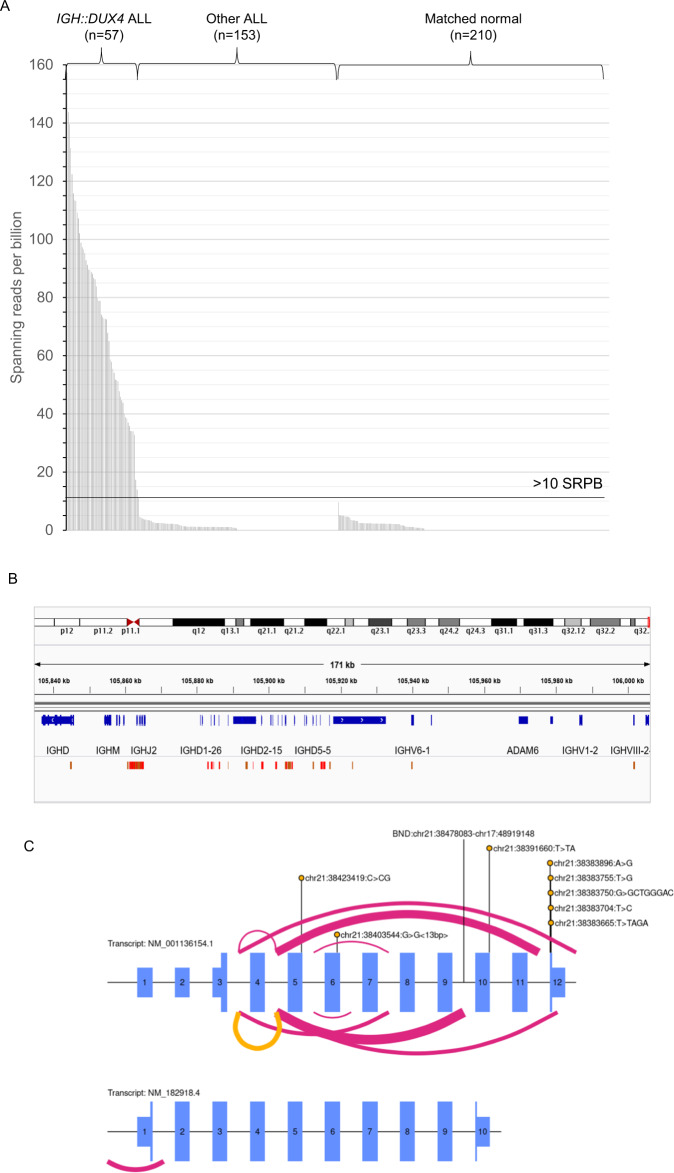
Characterisation of *DUX4*-r patients. **A** Spanning reads per billion (SRPB) between the *IGH* and *DUX4* loci. *IGH::DUX4* patients (*n* = 57) were found to have 11.1-157.3 SRPB. ALL samples from other subtypes (and matched germline samples) show lower values, ranging from 0–9.6 SPRB. A threshold of >10 SRPB was applied to define patients with *IGH::DUX4* abnormalities. Three *DUX4*-r patients did not show >10 SRPB as the rearrangement involved alternative genomic regions. **B** Breakpoint mapping of *IGH::DUX4* breakpoint within the IGH locus of 53 patients. Breakpoints mapping to the forward (red) or reverse (brown) strand are shown. A cluster breakpoint region (CBR) within the IGH J (joining) segment is present, in which 47/53 cases harbour a breakpoint (chr14:105860602-105865246). **C** *ERG* abnormalities are seen in 68.3% (41/60) of *DUX4*-r patients. The exon structure of *ERG* is depicted in NM_001136154.1 and NM_182918.4 (not to scale); exons are numbered and represented with purple rectangles. The type of abnormalities range from deletion (pink), inversion (yellow), mutation (lollipop stick) and translocation (lollipop stick labelled ‘BND’). The width of the ribbon represents the number of cases with the abnormality.

We developed a novel, customised analytical approach that identified 57 B-ALL patients with >10 spanning reads per billion (SRPB) between *IGH* and *DUX4* (Fig. 5A, Supplementary Information, Supplementary Table 6). There was complete concordance in detection of *DUX4*-r among cases with both WTS and WGS (*n* = 21), including the case with *DUX4::MYB* and the patient with fewest supporting reads by WGS (SRPB, 11.1), demonstrating the accuracy of WGS in *DUX4*-r subtyping. Although all 21 *DUX4*-r cases had global transcriptome profiles associated with *DUX4*-r and overexpression of *DUX4*, an *IGH::DUX4* fusion was only observed in 14/21 of the cases by WTS, while overlapping levels of *DUX4* expression were found in non-*DUX4*-r cases (Supplementary Fig. 4). These discrepancies demonstrate the requirement for a comparator cohort and validated analyses before relying solely on WTS for accurate *DUX4*-r classification.

It is estimated that 11-100 near-identical copies of *DUX4* are repeated within the subtelomeric regions of chromosomes 4 and 10 [15]. To improve their characterisation, *de novo* assembly of sequencing reads in 53 *IGH::DUX4* cases generated an average of 2.09 contigs/scaffolds per patient sample (Supplementary Table 7). The contigs/scaffolds revealed a common breakpoint region (CBR) within the *IGH* locus, chr14:105860602-105865246, in which 47/53 cases harboured a breakpoint (Fig. 5B). Interestingly, a second *IGH* breakpoint >100 kb distant from the first was observed in 24/53 of cases. Although 28% of *DUX4*-aligned sequences mapped to the chromosome 4 and 10 reference sequences with similar identity, 42% and 30% of *DUX4*-aligned sequences mapped more precisely to chromosome 4 or 10, respectively. In some cases, there was evidence of more complex rearrangement patterns, including *IGH::DUX4::IGH* to *IGH::IGH::DUX4* (Supplementary Fig. 5). The transcriptional consequence of these complex rearrangement patterns could not be explored due to a lack of cases with matched WTS data.

### Detection of focal abnormalities important for improved genetic classification of ALL

The UKALL-CNA classifier defines prognostic subtypes based on focal copy number changes in eight key genes/regions associated with B-ALL [33]. Furthermore, *ERG* deletions are considered to be a surrogate marker of *DUX4*-r due to them being found exclusively in this B-ALL subtype [10, 17, 34]. Somatic genetic abnormalities were detected in these key genes/regions in 172/208 patients from the WGS T-N cohort (Supplementary Table 8). The relative incidence and size of the focal genetic CNA varied between subtypes, as previously described (Supplementary Fig. 6, and Supplementary Table 9) [33, 35]. Among 367 genetic abnormalities observed by WGS, 332 involved CNA used in the UKALL-CNA classifier or within *ERG*, including seven focal deletions within PAR1 on chromosome X/Y, resulting in *P2RY8::CRLF2* fusion. The remaining variants (*n* = 35) involved whole chromosome/chromosome arm gains, which are not included in the UKALL-CNA classifier.

Parallel WGS and MLPA data were available for 304/332 CNA observed by WGS within the UKALL-CNA classifier. Results were concordant for 242/304, while the remaining 62 CNA were called by WGS only (Supplementary Table 8). By MLPA, CNA were not called if: (1) the copy number level was outside the detection threshold (MLPA probe ratio <0.75 for deletions) (31/62 CNA) or (2) there was no or single MLPA probe coverage (≥two successive probes must be abnormal to call CNA by MLPA) (31/62 CNA) (Supplementary Fig. 7). These findings indicate that the recommended cut-off levels for positive MLPA results may be too stringent, as evidenced by the *IKZF1* exonic duplications. They were exclusively observed in *IKZF1* N159Y patients (*n* = 3), however they were not called by MLPA in two patients because the gains were restricted to a single probe (Supplementary Fig. 7B).

In this study, *ERG* abnormalities were observed in *DUX4*-r patients at an incidence of 68% (41/60) by WGS, compared to 35% (21/60) by MLPA. Most patients harboured a single *ERG* abnormality, but two distinct genetic variants were identified in five patients. The range of *ERG* abnormalities included deletion (*n* = 34), mutation (*n* = 8), inversion (*n* = 3) and translocation (*n* = 1) (Fig. 5C). Importantly, copy number profiling methods will only detect *ERG* deletions, while they will miss the small variants and translocations detectable by WGS.

## Discussion

In this study, we have shown the excellent performance of WGS as a standalone diagnostic genetic screen in childhood B-ALL. T-only analysis provided rapid and accurate detection of those clinically-important genetic abnormalities required for risk stratification for treatment in a greater number of cases than standard-of-care techniques. In particular, we showed that WGS was most effective for the detection of the rapidly increasing list of newly reported and cytogenetically-cryptic abnormalities [7, 33, 36]. Combining T-only with T-N analysis, using appropriate germline samples and accepted mean sequencing depths provides fully comprehensive analysis of somatic variants for discovery of novel abnormalities and a deeper understanding of associated genetic changes.

Molecular subtypes defined by a range of genetic abnormalities present a challenge for accurate classification by current standard-of-care diagnostic tests. For example, *PAX5*alt and *ETV6::RUNX1*-like patients have unique gene expression profiles, but the driving genetic abnormalities are diverse and often undefined by WTS [5, 6]. Here, we have shown the significant contribution made by WGS in defining the complex genomic landscape underlying the *PAX5*alt and *ETV6::RUNX1*-like subtypes, highlighting some advantages of WGS over WTS, including detection of focal CNA and other rearrangements not involving fusion genes. Robust detection of all subtype-defining genetic abnormalities is key to future improvements in risk-directed therapy. Thus, the development of a DNA-based molecular schema that has been validated for accurate B-ALL subtyping using WGS in this way is timely.

In this study, we reported MAPK pathway gene fusions and *CNTNAP3B* abnormalities as recurrent changes in B-ALL. The latter has been previously reported in infant *KMT2A*-r ALL [32]. Such abnormalities may emerge as novel subtype-defining genomic changes in expanded patient cohorts. Furthermore, using WGS a number of new genetic features were recently associated with B-ALL subtypes previously defined by WTS. These discoveries highlight the importance of the continual discovery element associated with comprehensive WGS analysis.

We are confident from the results presented here to recommend T-only analysis for sensitive detection of clinically-relevant genetic variants as a rapid diagnostic test for implementation of risk-directed treatment stratification. This statement is supported by the failure of only one genetic abnormality in a single patient to be called by T-only analysis. This unusual case harboured a *BCR::ABL1* fusion, formed through a complex rearrangement characteristic of templated insertion. This process leads to duplicated sequence surrounding double stranded breakpoints, as previously reported in multiple myeloma, and often involving the *MYC* gene [37]. The breakpoints surrounding *BCR::ABL1* were visible within the alignment (bam) files. Thus, due to the clinical importance of *BCR::ABL1* and ABL-class subtype detection in relation to their poor prognosis [2, 11, 38] and potential treatment with tyrosine kinase inhibitor (TKI) therapy [12, 39], we are developing bespoke automated calling approaches, as we achieved for *IGH::DUX4*. The ability for continual optimisation of such pipelines, in response to newly identified or difficult-to-detect genetic abnormalities, emphasises the potential for WGS to keep pace in routine diagnostic testing.

*IGH::DUX4* accounts for ~10% of B-other-ALL and is regarded as a genetic marker of good-risk [7, 40]. However, studies have been limited by small cohort sizes and/or heterogeneous therapies. We developed a novel automated bioinformatic pipeline to reliably identify 60 patients with *DUX4*-r within UKALL2003, representing the largest *DUX4*-r cohort within an individual clinical trial to date. Previous studies have shown that *IGH::DUX4* patients have a high incidence of *ERG* deletions, proposed as a surrogate marker to overcome difficulties in detection of *DUX4*-r [10, 23, 34, 41]. As around one third of *DUX4*-r cases do not have detectable *ERG* deletions, targeted identification of *DUX4*-r is a priority for accurate diagnosis. Furthermore, *ERG* abnormalities were detected in 68% of *DUX4*-r cases by WGS compared to 35% of the same cohort by MLPA. In addition, we have demonstrated the improved sensitivity of WGS to detect other patterns of secondary genetic deletions that predict treatment response (*IKZF1*-plus, UKALL-CNA-classifier), demonstrating the versatility of WGS in comprehensive detection of all levels of risk-stratifying genetic abnormalities [33, 36].

In an era in which genomics is driving enormous scientific progress and demonstrating the potential for precision medicine, this study endorses the clinical advantage of introducing WGS as a first line diagnostic test in childhood B-ALL. While accurately detecting the range of clinically-relevant cytogenetic abnormalities, it identified an expanded list of genetic abnormalities, which may define novel subtypes or co-operating genetic abnormalities in larger collaborative studies. Although the cost and infrastructural requirements of WGS has been limiting for many countries, the decreasing prices and rapidly expanding list of genetic tests required for accurate diagnosis are making WGS a viable option for some healthcare providers. Pipelines include prioritisation of genetic abnormalities that require an immediate clinical response, while providing wider access to sequencing, demographic and treatment data to allow new clinical associations to emerge. This study validates the importance of this new diagnostic service to detect clinically actionable genetic abnormalities and build a unique and invaluable resource for developing genetic-based risk stratification algorithms in the future.

## Supplementary information

Supplementary Information

## Data Availability

Sequencing data have been deposited in the European Genome-phenome Archive (EGA) under the Accession Code EGAS00001006863. Alternatively these data will be made available upon request from Prof Christine Harrison (christine.harrison@newcastle.ac.uk).
