# Supplementary material for: Whole genome sequencing provides comprehensive genetic testing in childhood B-cell acute lymphoblastic leukaemia
Source: Leukemia. 2023 Jan 19;37(3):518–28. doi: 10.1038/s41375-022-01806-8 (PMC9991920; doi:10.1038/s41375-022-01806-8)
Supplement: Supplementary file 1 — Supplementary Information [file 41375_2022_1806_MOESM1_ESM.pdf]

Supplementary Information

1. Patients and methods Page 2
   a. Whole genome sequencing Page 2
   b. *DUX4*-r detection Page 5
   c. Whole transcriptome sequencing (WTS) Page 7
   d. References Relevant to Supplementary Information Page 8

Supplementary Figures

Supplementary Figure 1: Detection of clinically-relevant chromosomal abnormalities by WGS.
Supplementary Figure 2: A complex *BCR::ABL1* fusion.
Supplementary Figure 3: *PAX5* and *ETV6* fusion genes are highly variable.
Supplementary Figure 4: *DUX4* (ENSG00000260596.5) transcript levels in B-ALL subtypes.
Supplementary Figure 5: Complex rearrangement pattern of *IGH::DUX4*
Supplementary Figure 6: Genetic abnormalities that affect nine key genes/regions in ALL.
Supplementary Figure 7: Samples per subtype with genetic abnormalities of nine key genes/regions that were detected by WGS but not called by MLPA

Supplementary Tables

Supplementary Table 1: Cohort information.
Supplementary Table 2: Validation of cytogenetic abnormalities characteristic of established B-ALL subtypes.
Supplementary Table 3: Subtype breakdown of individual patients by WGS and WTS.
Supplementary Table 4: Summary of subtype-defining genetic abnormalities per case.
Supplementary Table 5: Genetic abnormalities characteristic of *PAX5*alt and *ETV6::RUNX1*-like cases.
Supplementary Table 6: *IGH::DUX4* detection.
Supplementary Table 7: *IGH::DUX4* breakpoint mapping.
Supplementary Table 8: Genetic variants detected by WGS involving nine genes/regions.
Supplementary Table 9: UKALL-CNA risk group status of patients.

Patients and methods

a. Whole genome sequencing
Patient cohort

Diagnostic and matched post-treatment samples from 210 and 208 childhood B- lineage acute lymphoblastic leukemia (B-ALL) patients, respectively, were prepared for whole genome sequencing (WGS) (**Supplementary Table 1**). Samples were obtained from the Blood Cancer UK Childhood Leukaemia Cell Bank approved by the South West-Central Bristol Research Ethics Committee. Written informed consent was obtained from patients or parents, in accordance with the Declaration of Helsinki. Patients were divided into two distinct cohorts: 1) with known established chromosomal abnormalities used in risk-stratification for treatment (n=38) (*BCR::ABL1*, ABL-class fusion, *ETV6::RUNX1*, near-haploidy, high hyperdiploidy, low hypodiploidy, iAMP21-ALL, *KMT2A*-r and *TCF3::PBX1*), to validate the effectiveness of detection by WGS. 2) Patients from a single clinical trial (UKALL2003) with sufficient diagnostic and germline material available that were negative for the above abnormalities based on routine testing by cytogenetic and FISH analyses (according to recommended testing for *BCR::ABL1*, *ETV6::RUNX1* and *KMT2A*-r in UKALL2003, as previously described(1)). Of note, *TCF3::PBX1* patients were identified by chromosomal analysis only in UKALL2003, no *TCF* FISH was performed, explaining the high number that emerged in cohort 2.

Cohort 2 patients were termed "B-other-ALL" within this study. This cohort included a high number of cases with normal karyotypes (n=50) or failed cytogenetic results (n=23), for which limited material restricted the level of genetic testing at diagnosis in some patients. Patients with *CRLF2* rearrangements (*CRLF2*-r) were identified as previously described.(2, 3) They were under-represented in this study, because, patients with unknown subtype-defining genetic abnormalities were prioritized, thus known *CRLF2*-r patients were excluded. Additional FISH tests were performed following WGS to validate key genetic abnormalities in some cases; these data are provided in **Supplementary Table 1**.

Sample collection and preparation

Diagnostic samples comprised bone marrow cells collected prior to the start of treatment. DNA extracted from bone marrow cells after the start of treatment was used as a matched germline sample in 208 patients. The time point from which the germline sample was taken was available for 203 patients; 11% (23/203) were taken before the end of induction therapy (EoI) (week 5), as indicated in **Supplementary Table 1**. Induction therapy for B-ALL is highly effective at reducing disease burden; it was previously shown that 94% of B-ALL patients have <1% leukaemic blast cells in their bone marrow at EoI.(4) Of the 23 cases with samples taken before EoI, 13 had leukemic blast counts <0.05%, while six had leukemic blast counts ≥1% (range, 1%-90%), while four samples had missing data, potentially representing germline samples contaminated with leukaemic cells. Patients with detectable levels of leukaemic blasts in their germline samples (minimal residual disease, MRD) were problematic for T-N data analyses, which was particularly relevant among patients with *BCR::ABL1* and *EBF1::PDGFRB* fusions, who tend to be refractory or respond slowly to initial therapy.(5-7)

To prepare the sequencing libraries, 1-3ug of high-quality double-stranded DNA (ds-DNA) was extracted from bone marrow aspirates using the DNeasy Extraction kit (Qiagen, Manchester, UK). The Qubit® dsDNA BR (Broad-Range) Assay kit was used to quantify dsDNA concentrations (Thermo Fisher Scientific, California, USA). WGS libraries were prepared using the TruSeq® DNA PCR-Free Library Preparation kit (Illumina) with 600ng input DNA. Libraries were sequenced as paired-ends (2 × 150 cycles) on a HiSeqX platform to a mean depth of >38x for germline and >76x for diagnostic samples. We assessed the effect of sequencing depth on the ability of WGS to detect structural variants from information of clonality (based on FISH results). We were able to detect structural variants when present in <20% of cells (based on FISH data) and with less than 18x in the diagnostic sample.

A small initial subset of samples were prepared with 500ng of input DNA using a pre-released version of the TruSeq PCR free protocol and libraries were sequenced as 100 bp paired-end reads on a Hiseq2500.

WGS data analysis

DNA sequence reads of the diagnostic and remission samples were aligned to human reference genome GRCh38 using the Whole Genome Sequencing pipeline (WGSv8) with the Illumina Isaac aligner (SAAC01325.18.01.29) and the removal of duplicate read-pairs.(8) Genetic variants were detected from the sequencing reads of diagnostic and remission DNA using Strelka for small variants, defined as single nucleotide variants (SNV) and indels <50bp.(9) Manta was employed for the identification of structural variants (SV) >50bp (deletions, duplications, inversions, insertions and translocations) and Canvas was used for the discovery of copy number variants.(10, 11) The Tumour Normal pipeline (TNWv6) used the somatic joint-calling mode in Strelka, Manta and Canvas to call somatic small and structural variants and copy number alterations. The version and description of individual software used in WGSv8 and TNWv6 in this study are summarised in the tables below.

Whole Genome Sequencing pipeline (WGSv8). Version and description of individual software used in WGSv8 in this study.

Software	Version	Description
WGS analysis pipeline	7.8.0.627	Overall pipeline of algorithms used for analysis
Isaac Aligner	SAAC01325.18.01.29	Align reads to the human GRCh38 reference
Strelka Variant Caller	2.9.2	Germline SNV and indel caller
Canvas (CNV Caller)	1.35.2.7	Germline copy number variant caller
Manta (SV Caller)	1.3.2	Germline structural variant caller
Nirvana	2.0.8	Variant annotation against a variety of sources
DataCollectionVersion	91.26.44	Annotation of data

Tumour Normal pipeline (TNWv6). Version and description of individual software used in TNWv6 in this study.

Software	Version	Description
TumorNormalWorker	7.4.0.78	Overall pipeline of algorithms used for analysis
Strelka Variant Caller	2.9.4	Somatic small variant caller
Canvas (CNV Caller)	1.38.0.1554	Copy number aberration caller
Manta (SV Caller)	1.3.2	Somatic structural variant caller
Nirvana	2.0.11	Annotation of somatic variants
DataCollectionVersion	91.26.44	Annotation of data

Tumour-only (T-only) analysis was performed on all patients with established genetic abnormalities of clinical significance (n=38, cohort 1) and diagnostic samples without a matched germline (n=2), using DRAGEN v3.7.5.(12)

Germline and somatic small variants, copy number changes and structural variants were annotated by Nirvana using Ensembl Human Genome v91 gene annotations as described in the table below.(13, 14) Mutational signatures were assessed using the counts of each mutation- type (C>A/G>T, C>G/G>C, C>T/G>A, T>A/A>T, T>C/A>G, T>G/A>C) at each trinucleotide in the reference genome as previously described, using COSMIC version 3 release v89 (May 2019).(15) The sum of SBS2 and SBS13 was used to identify cases with >5% of mutations attributed to the AID/APOBEC family of cytidine deaminases. The criteria for iAMP21-ALL diagnosis was at least one BFB cycle, stepwise gain in copy number up to the highest region of gain on the chromosome 21 long arm and/or chromothripsis of chromosome 21, as previously described.(16)

Annotation summary of Ensembl Human Genome v91. Version of databases used to annotate genetic variants in Ensembl Human Genome v91. Annotation data collection 91.26.44.

Annotation	Version
Ensembl	Human v91 GRCh38
ClinVar	29/01/2018
COSMIC	v84
dbSNP	v150
ExAC	v0.3.1
DGV	15/05/2016
gnomAD	2.0.2
MITOMAP	28/02/2018
OMIM	13/02/2018
1000 Genomes	Phase 3 v5a
phyloP	2009-11-10

b. *DUX4*-r detection

Detection of *IGH::DUX4* is challenging as the DNA sequences are repetitive and variable at both breakpoints. Repetitive sequence is problematic for the detection of other *DUX4* rearrangements that do not involve *IGH*. Thus, a customised pipeline was developed for the detection of rearrangements involving *DUX4* (with *IGH* or other partner genes).

IGH::DUX4

Two D4Z4 macrosatellite arrays are located proximal to the subtelomeric region of chromosomes 4 and 10. Each array contains 11 to more than 100 repeated units. Each repeat unit of D4Z4 is approximately 3.3-kb in length and contains an open reading frame (ORF) encoding the *DUX4* gene. These ORFs have >99% sequence similarity within each array and >98% similarity between arrays. Therefore, fragmented reads from *DUX4* can be aligned at multiple positions on the reference and due to this uncertainty are frequently assigned alignment MAPQ scores of 0. The *IGH* locus contains multiple copies of the repetitive IGHV gene-containing segments that range in size (4 to 24-kb) and exhibit high sequence similarity.

A review of samples harbouring *ERG* deletions, associated with *IGH::DUX4* rearrangements in previous studies, was able to identify the characteristic clusters of reads pairs spanning putative *IGH::DUX4* breakpoints in some but not all samples.(17-19) An investigation of the samples where putative *IGH::DUX4* breakpoints could be detected revealed that within these samples the spanning reads often mapped to different D4Z4 repeat units and to different arrays. The hypothesis was that all *ERG* deleted samples have multiple reads spanning their *IGH::DUX4* breakpoint but in most samples those read pairs are mapped to many different repeats units and the expected cluster of spanning reads is not present. It was tested by identification of all paired reads that mapped to both the *IGH* locus and the D4Z4 repeat array to determine if: 1) they were in excess in *ERG* deleted samples, 2) if after local assembly, the longer contigs mapped to a unique set of breakpoints, where one read mapped to a D4Z4 repeat array (chr4:189950000-190214555 or chr10:133614430-133797422 in GRCh38) and its pair mapped to the *IGH* locus (chr14:105536437-106929844 in GRCh38). This analysis was performed on 210 B-ALL and 208 matched germline samples.

The germline samples were split into a training set (n=100 samples) and the remaining samples in the cohort (n=318) were used as a validation set. The count of all spanning read pairs per sample was normalized to total read count. For each sample, the reads mapping to the *IGH* locus were placed into 1000bp non-overlapping bins. In the training set, the bin (chr14:1086883000- 1086883999) contained ≥5 reads. All read pairs that mapped between these coordinates were excluded from further analysis of all samples. Spanning read pairs from all samples with >10 spanning reads per billion (SRPB) were locally assembled using SOAP21 and k-mers 15, 31, 41, 51.(20) Not all k-mers generated successful assemblies, thus the assembly that generated the longest scaffold was chosen for further analysis. All assembled contigs and scaffolds were aligned to the GRCh38 reference genome using BLASTN2.(21) Any contigs or scaffolds that mapped entirely within the D4Z4 microsatellite array or the *IGH* locus were excluded. The remaining contigs were used to determine the unique set of breakpoints in the rearrangements between *IGH* and *DUX4*.

***DUX4*-r detection involving other partner genes**

Although *IGH* is the most common partner gene of *DUX4*, other partner genes have been reported in ALL, such as *ERG*.(19) A similar approach to *IGH::DUX4* detection was used to identify cases that harbour *DUX4*-r involving other (non-*IGH*) partner genes. Briefly, all paired reads that mapped to both the D4Z4 repeat array and any other genomic region were identified. The training set of 100 germline samples identified 14x 1000bp bins in five regions of the genome (equivalent to <0.0001% of the genome) that had ≥6 reads mapping between the region and the *DUX4* locus. These 14 regions, detailed in the table below, were excluded from analysis, as non-specific mapping may lead to the false positive detection of *DUX4*-r.

Regions excluded from *DUX4*-r detection involving other (non-*IGH*) partner genes.
Chromosome and start and end co-ordinate of the 14x 1000bp binned regions is shown.

Chromosome	Start position	End position
chr18	105000	106000
chr18	106000	107000
chr18	107000	108000
chr18	108000	109000
chr18	109000	110000
chr18	110000	111000
chr18	112000	113000
chr2	32916000	32917000
chr20	29411000	29412000
chr20	30811000	30812000
chr20	30812000	30813000
chr22	12692000	12693000
chr22	12693000	12694000
chr3	75669000	75670000

This analysis identified two new *DUX4* partner genes: 1) *DUX4::MYB* (patient #23445) with 61 spanning reads and 2) *DUX4::DNTT* (patient #11148) with 23 spanning reads. We have been unable to identify the partner gene in a third patient with an *ERG* frameshift mutation (frameshift_variant; p.(Leu297PhefsTer12); VAF=0.45). Due to the exclusive association of *ERG* abnormalities with *DUX4*-r ALL,(19, 22) it is highly likely that an undetected breakpoint is located within a binned region or a region not represented in GRCh38.

c. Whole transcriptome sequencing (WTS)

Sample preparation

Total RNA or mRNA sequencing was performed on 85 B-ALL samples. The quality and concentration of RNA was assessed using the Agilent RNA 6000 Nano Kit on the Agilent 2100 Bioanalyzer system (Agilent Technologies, Santa Clara, USA). Libraries were prepared for 68 patients using the TruSeq® Stranded Total RNA Library Preparation Globin kit (Illumina) using 100ng RNA for samples of RIN □6 and 200ng for samples of RIN <6. Libraries were sequenced on the Illumina NovaSeq 6000 using 75 base paired-end chemistry (110-315 million read-pairs per sample). Furthermore, 40 samples were prepared using the TruSeq® Stranded mRNA Library Prep sequencing kits (Illumina) and sequenced on the Illumina HiSeq 2500 using 125 base paired-end chemistry by AROS Applied Biotechnology A/S (Aarhus, Denmark) and Eurofins Genomics (Ebersberg, Germany).

Data analysis

Sequencing reads were aligned to human reference genome GRCh38 and transcript variants were identified using RNA-Seq alignment v2.0.1. The version and description of individual software used in RNA sequencing alignment v2.0.1 is provided in the table below.

RNA-Seq alignment v2.0.1. The version and description of individual software used in RNA-seq alignment v2.0.1 pipeline.

Software	Version	Description
Isas	3.19.1.12+master	Overall pipeline of algorithms used for analysis
Star	2.6.1a	Aligner
Strelka Variant Caller	2.9.9	Small variant caller
Samtools	0.1.20	
Salmon	0.11.2	Transcript and gene quantification
Manta (SV Caller)	1.4.0	RNA structural variant caller
Nirvana	2.0.10.0	Annotation of RNA variants
DataCollectionVersion	91.26.44	Annotation of data

B-ALL patients were subtyped by combining the samples from this study (n=85) with a reference cohort containing 1452 B-ALL samples from Gu *et al*.(23) t-distributed stochastic neighbor embedding (tSNE) projection, Prediction Analysis of Microarrays (PAM) classifier, SNV calling and fusion gene detection was used for determining the subtypes as previously described.(24) Briefly, sequencing reads were mapped to the GRCh38 human reference genome by STAR and read counts estimated by RSEM tool.(25, 26) DESeq2 Bioconductor R package and varianceStabilizingTransformation command was used for sample normalization.(27) Batch effect correction based on library preparation strategies and sequencing lengths was performed using the ComBat function in sva R package.(28) PAM was used to identify subgroups as previously described.(23) tSNE implemented in Rtsne R package was used to visualize subtype clusters in two-dimensions. Arriba, Fusioncather, Pizzly, SQUID, STAR-fusion were used for detecting fusion genes for the 85 samples.(29-33) Fusions were manually reviewed. GATK4 was used for detecting key SNVs for *IKZF1* (N159Y), *PAX5* (P80R) and *ZEB2* (H1038R) from WTS data following the GATK best practices.(34) Variants were annotated using ANNOVAR.(35)

The Ph-like (or *BCR::ABL1*-like) group is a subtype defined by WTS in which patients have a similar gene expression profile to *BCR::ABL1* positive patients.(36) At the genomic level, it includes patients with abnormalities involving genes in the JAK-STAT pathway: including *CRLF2-r* (*IGH::CRLF2* and *P2RY8::CRLF2*) and *JAK*-r; and samples with ABL-class fusions involving the genes *ABL1, ABL2, PDGFRB and CSF1R.*(2)

d. References Relevant to Supplementary Information

1. Harrison CJ, Moorman AV, Barber KE, Broadfield ZJ, Cheung KL, Harris RL, et al. Interphase molecular cytogenetic screening for chromosomal abnormalities of prognostic significance in childhood acute lymphoblastic leukaemia: a UK Cancer Cytogenetics Group Study. British journal of haematology. 2005;129(4):520-30.
2. Schwab CJ, Murdy D, Butler E, Enshaei A, Winterman E, Cranston RE, et al. Genetic characterisation of childhood B-other-acute lymphoblastic leukaemia in UK patients by fluorescence in situ hybridisation and Multiplex Ligation-dependent Probe Amplification. British journal of haematology. 2021;196(3):753-63.
3. Ensor HM, Schwab C, Russell LJ, Richards SM, Morrison H, Masic D, et al. Demographic, clinical, and outcome features of children with acute lymphoblastic leukemia and CRLF2 deregulation: results from the MRC ALL97 clinical trial. Blood. 2011;117(7):2129-36.
4. O'Connor D, Enshaei A, Bartram J, Hancock J, Harrison CJ, Hough R, et al. Genotype-Specific Minimal Residual Disease Interpretation Improves Stratification in Pediatric Acute Lymphoblastic Leukemia. Journal of clinical oncology : official journal of the American Society of Clinical Oncology. 2018;36(1):34-43.
5. den Boer ML, Cario G, Moorman AV, Boer JM, de Groot-Kruseman HA, Fiocco M, et al. Outcomes of paediatric patients with B-cell acute lymphocytic leukaemia with ABL-class fusion in the pre-tyrosine-kinase inhibitor era: a multicentre, retrospective, cohort study. Lancet Haematol. 2021;8(1):e55-e66.
6. Cario G, Leoni V, Conter V, Attarbasc A, Zaliova M, Sramkova L, et al. Relapses and treatment-related events contributed equally to poor prognosis in children with ABL-class fusion positive B-cell acute lymphoblastic leukemia treated according to AIEOP-BFM protocols. Haematologica. 2020;105(7):1887-94.
7. Schwab C, Ryan SL, Chilton L, Elliott A, Murray J, Richardson S, et al. EBF1-PDGFRB fusion in pediatric B-cell precursor acute lymphoblastic leukemia (BCP-ALL): genetic profile and clinical implications. Blood. 2016;127(18):2214-8.
8. Raczy C, Petrovski R, Saunders CT, Chorny I, Kruglyak S, Margulies EH, et al. Isaac: ultra-fast whole-genome secondary analysis on Illumina sequencing platforms. Bioinformatics. 2013;29(16):2041-3.
9. Kim S, Scheffler K, Halpern AL, Bekritsky MA, Noh E, Kallberg M, et al. Strelka2: fast and accurate calling of germline and somatic variants. Nat Methods. 2018;15(8):591-4.
10. Chen X, Schulz-Trieglaff O, Shaw R, Barnes B, Schlesinger F, Kallberg M, et al. Manta: rapid detection of structural variants and indels for germline and cancer sequencing applications. Bioinformatics. 2016;32(8):1220-2.
11. Roller E, Ivakhno S, Lee S, Royce T, Tanner S. Canvas: versatile and scalable detection of copy number variants. Bioinformatics. 2016;32(15):2375-7.
12. Illumina. DRAGEN v3.7.5 Software Release Notes. 2021(1000000142362_00):18.
13. Stromberg M, Roy R, Lajugie J, Jiang Y, Li H, Margulies E. Nirvana: Clinical Grade Variant Annotator. ACM-BCB '17: Proceedings of the 8th ACM International Conference on Bioinformatics, Computational Biology,and Health Informatics. 2017.
14. Yates AD, Achuthan P, Akanni W, Allen J, Allen J, Alvarez-Jarreta J, et al. Ensembl 2020. Nucleic Acids Res. 2020;48(D1):D682-D8.
15. Alexandrov LB, Nik-Zainal S, Wedge DC, Aparicio SAJR, Behjati S, Biankin AV, et al. Signatures of mutational processes in human cancer. Nature. 2013;500(7463):415-21.
16. Li Y, Schwab C, Ryan S, Papaemmanuil E, Robinson HM, Jacobs P, et al. Constitutional and somatic rearrangement of chromosome 21 in acute lymphoblastic leukaemia. Nature. 2014;508(7494):98-102.
17. Lilljebjorn H, Henningsson R, Hyrenius-Wittsten A, Olsson L, Orsmark-Pietras C, von Palffy S, et al. Identification of ETV6-RUNX1-like and DUX4-rearranged subtypes in paediatric B-cell precursor acute lymphoblastic leukaemia. Nat Commun. 2016;7:11790.
18. Liu YF, Wang BY, Zhang WN, Huang JY, Li BS, Zhang M, et al. Genomic Profiling of Adult and Pediatric B-cell Acute Lymphoblastic Leukemia. EBioMedicine. 2016;8:173-83.
19. Zhang J, McCastlain K, Yoshihara H, Xu B, Chang Y, Churchman ML, et al. Deregulation of DUX4 and ERG in acute lymphoblastic leukemia. Nature genetics. 2016;48(12):1481-9.

20. Luo R, Liu B, Xie Y, Li Z, Huang W, Yuan J, et al. SOAPdenovo2: an empirically improved memory-efficient short-read de novo assembler. Gigascience. 2012;1(1):18.
21. Altschul SF, Gish W, Miller W, Myers EW, Lipman DJ. Basic local alignment search tool. J Mol Biol. 1990;215(3):403-10.
22. Zaliova M, Potuckova E, Hovorkova L, Musilova A, Winkowska L, Fiser K, et al. ERG deletions in childhood acute lymphoblastic leukemia with DUX4 rearrangements are mostly polyclonal, prognostically relevant and their detection rate strongly depends on screening method sensitivity. Haematologica. 2019;104(7):1407-16.
23. Gu Z, Churchman ML, Roberts KG, Moore I, Zhou X, Nakitandwe J, et al. PAX5-driven subtypes of B-progenitor acute lymphoblastic leukemia. Nature genetics. 2019;51(2):296-307.
24. Jeha S, Choi J, Roberts KG, Pei D, Coustan-Smith E, Inaba H, et al. Clinical significance of novel subtypes of acute lymphoblastic leukemia in the context of minimal residual disease-directed therapy. Blood Cancer Discovery. 2021.
25. Pui C-H, Yang JJ, Hunger SP, Pieters R, Schrappe M, Biondi A, et al. Childhood Acute Lymphoblastic Leukemia: Progress Through Collaboration. Journal of Clinical Oncology. 2015;33(27):2938-48.
26. Li B, Dewey CN. RSEM: accurate transcript quantification from RNA-Seq data with or without a reference genome. BMC Bioinformatics. 2011;12(1).
27. Anders S, Huber W. Differential expression analysis for sequence count data. Genome Biology. 2010;11(10).
28. Leek JT, Johnson WE, Parker HS, Jaffe AE, Storey JD. The sva package for removing batch effects and other unwanted variation in high-throughput experiments. Bioinformatics. 2012;28(6):882-3.
29. Uhrig S, Ellermann J, Walther T, Burkhardt P, Fröhlich M, Hutter B, et al. Accurate and efficient detection of gene fusions from RNA sequencing data. Genome Research. 2021;31(3):448-60.
30. Nicorici D, Satalan M, Edgren H, Kangaspeska S, Murumagi A, Kallioniemi O, et al. 2014.
31. Bray NL, Pimentel H, Melsted P, Pachter L. Near-optimal probabilistic RNA-seq quantification. Nature Biotechnology. 2016;34(5):525-7.
32. Ma C, Shao M, Kingsford C. SQUID: transcriptomic structural variation detection from RNA-seq. Genome Biology. 2018;19(1).
33. Haas BJ, Dobin A, Stransky N, Li B, Yang X, Tickle T, et al. 2017.
34. McKenna A, Hanna M, Banks E, Sivachenko A, Cibulskis K, Kernytsky A, et al. The Genome Analysis Toolkit: A MapReduce framework for analyzing next-generation DNA sequencing data. Genome Research. 2010;20(9):1297-303.
35. Wang K, Li M, Hakonarson H. ANNOVAR: functional annotation of genetic variants from high-throughput sequencing data. Nucleic Acids Research. 2010;38(16):e164-e.
36. Roberts KG, Li Y, Payne-Turner D, Harvey RC, Yang YL, Pei D, et al. Targetable kinase-activating lesions in Ph-like acute lymphoblastic leukemia. N Engl J Med. 2014;371(11):1005-15.

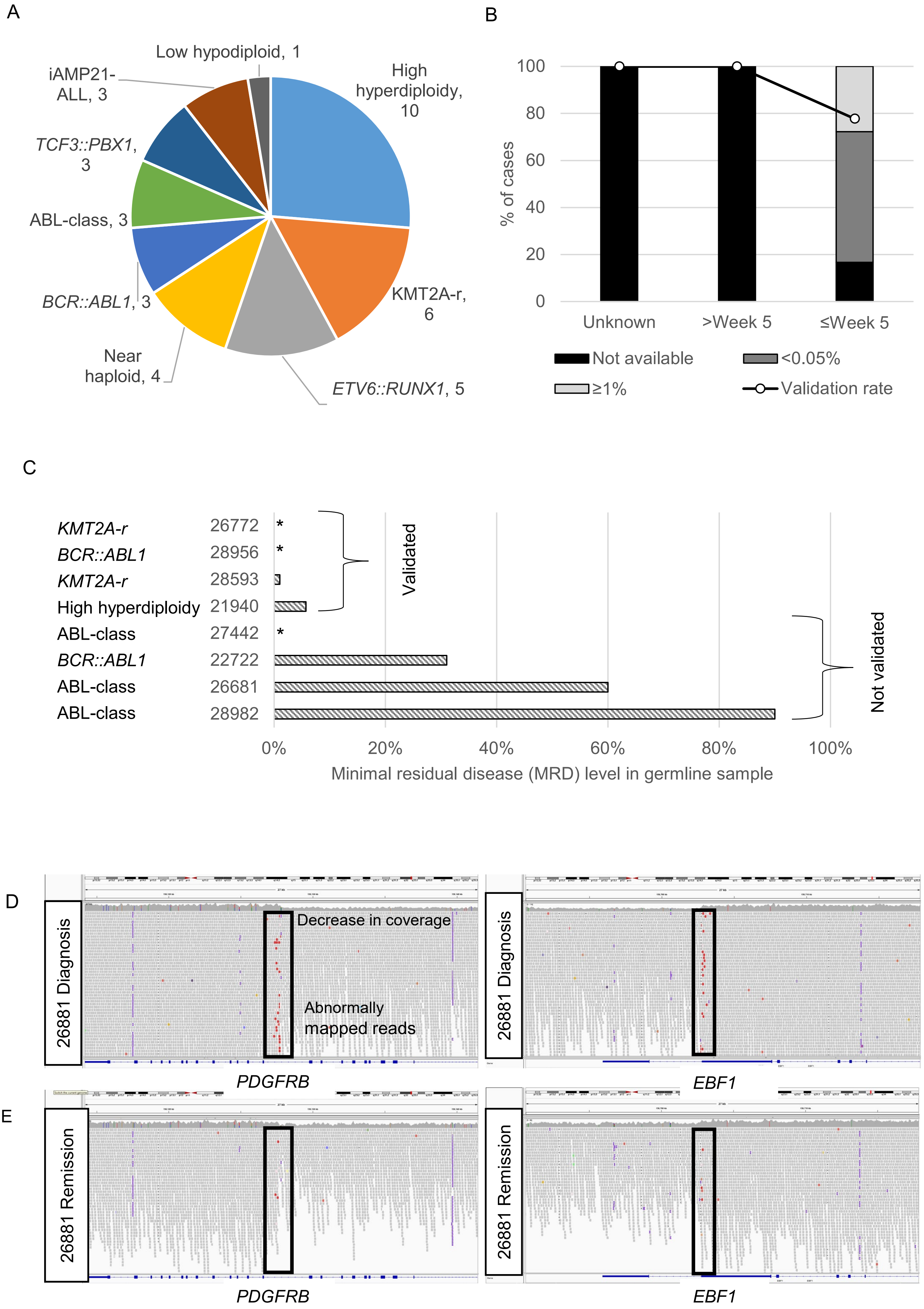
A
Low hypodiploid, 1
High hyperdiploidy, 10
iAMP21-ALL, 3
TCF3::PBX1, 3
ABL-class, 3
BCR::ABL1, 3
Near haploid, 4
ETV6::RUNX1, 5
KMT2A-r, 6
B
% of cases
100
80
60
40
20
0
Unknown
>Week 5
≤Week 5
Not available
<0.05%
≥1%
Validation rate
C
KMT2A-r 26772 *
BCR::ABL1 28956 *
KMT2A-r 28593
High hyperdiploidy 21940
ABL-class 27442 *
BCR::ABL1 22722
ABL-class 26681
ABL-class 28982
Validated
Not validated
0%
20%
40%
60%
80%
100%
Minimal residual disease (MRD) level in germline sample
D
26881 Diagnosis
Decrease in coverage
Abnormally mapped reads
PDGFRB
EBF1
E
26881 Remission
PDGFRB
EBF1

Supplementary Figure 1:
Detection of clinically-relevant chromosomal abnormalities by WGS. (A) WGS subtype classification of 38 patients with established chromosomal abnormalities of clinical significance. (B) The validation rate, defined as the proportion of cases in which WGS concurred with current standard-of-care testing (cytogenetic analysis, FISH), is shown for each germline timepoint. Matched germline samples were taken before (<5 weeks (n=18)) or after (≥5 weeks (n=19)) the end of induction (EOI) therapy. One patient had a germline sample from an unknown timepoint. MRD levels in germline samples taken before EOI was <0.05% (dark grey bar), ≥1% (light grey bar) or not available (black) in ten (55.6%), five (27.8%) and three (16.7%) patients, respectively. (C) Eight patients with germline samples taken before the EOI had MRD levels not available (*) or ≥1% (individual bars). Clinically-relevant subtype-defining chromosomal abnormalities were not validated by WGS in four patients; *BCR::ABL1* (n=1) or *EBF1::PDGFRB* (n=3). (D-E) *EBF1::PDGFRB* fusion was detected in the diagnostic (D) and matched germline (E) sample of patient #26881. Inspection of reads aligned to the region surrounding the rearrangement, which fuses *EBF1* exon 1-15 to *PDGFRB* exons 11-23, showed inappropriately mapped paired-end reads (red sequencing reads in highlighted window), indicative of a deletion. A decrease in coverage of mappable reads within the region confirms a deletion. The germline sample was taken at the EOI (day 28), when residual leukaemic blasts (MRD) represented 60% of the sample. Poor response to induction therapy, characterised by high MRD levels at the EOI, has been reported in *EBF1::PDGFRB* B-ALL.(1,2)

References:
1. Schwab C, Ryan SL, Chilton L, Elliott A, Murray J, Richardson S, et al. EBF1-PDGFRB fusion in pediatric B-cell precursor acute lymphoblastic leukemia (BCP-ALL): genetic profile and clinical implications. Blood. 2016;127(18):2214-8.
2. den Boer ML, Cario G, Moorman AV, et al. Outcomes of paediatric patients with B-cell acute lymphocytic leukaemia with ABL-class fusion in the pre-tyrosine-kinase inhibitor era: a multicentre, retrospective, cohort study. *Lancet Haematol*. 2021;8(1):e55-e66.

A

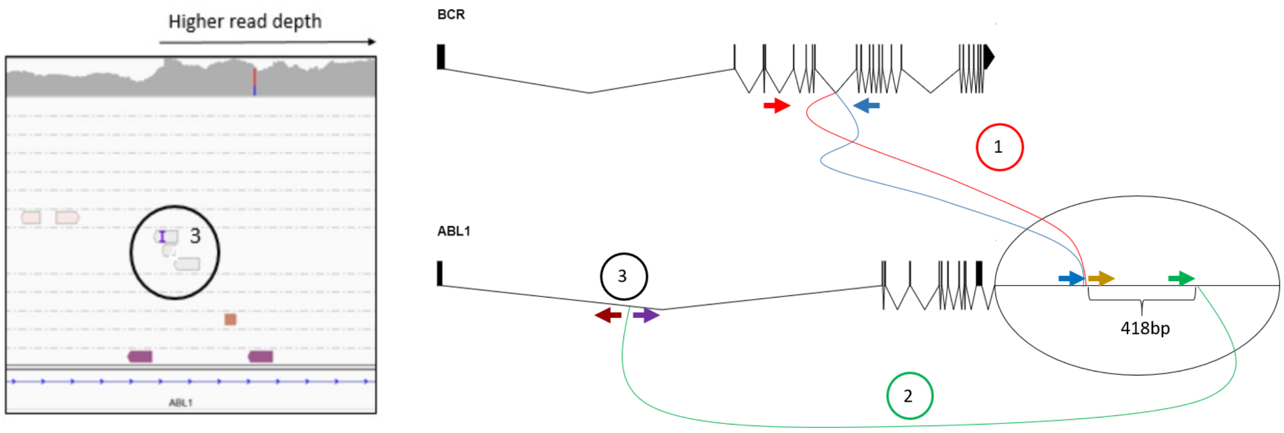

B

[read 1]
NGGCAGTAACAAGGATTTTATTTTCTGTTTTTGCCAATGACAAGTTTGGTCATAAAGTAATCATGTTTTTG
ATTTTTTTTTGTTTTTTTTTTACCTCCCACCCCCGGCAGGGTGGAGGTTGCAGCACGCTATGATCGTGCA
ACCGTATTC…………………………………………………………………….. [read 2]
GTGCTTGGATTACAGGCATGAGCCAGGGCGCCCGGCCCGCCACTTTTTTTTTTTTGTCTGTCATTTGTG
GAAGCTAGCACAACAGTTTAAACGGAGGCCCATAGGCCATGTCTAAATATTTAAAAGTTACAAATGTGAC
AGACTGTGAAA

Read #	Chr	Start position	End position	Strand	Span	Genomic location
Read 1	22	23272114	23272174	+	61bp	Intron 6 of *BCR*
Read 1	9	130889292	130889321	+	30bp	Downstream of *ABL1*
Read 2	9	130889631	130889685	+	55bp	Downstream of *ABL1*
Read 2	9	130790043	130790140	+	98bp	Intron 1 of *ABL1*

Supplementary Figure 2: A complex *BCR::ABL1* fusion, formed through two rearrangements, in patient 22722. The mate-pairs of rearrangement 1 (red) map to intron 6 of *BCR* and downstream of *ABL1*. While rearrangement 2 (green) had one mate located 418bp away from rearrangement 1 and the mate-pair mapped within intron 1 of *ABL1*. This complex rearrangement pattern will produce an in-frame *BCR::ABL1* fusion (*BCR* exon 1-6 fused to *ABL1* exon 2-11) for which there was weak read support; this is highlighted by three reads with poor mapping quality and duplication of *ABL1* exon 2-11, as depicted by the higher read depth (3). (B) Alignment of paired-reads (labelled read 1 and read 2) that were split across genomic regions, demonstrating the *BCR::ABL1* fusion between intron 6 of *BCR* and intron 1 of *ABL1* via a number of rearrangements. The sequence in the read that mapped to a specific location in the table and in (A) are colour co-ordinated.

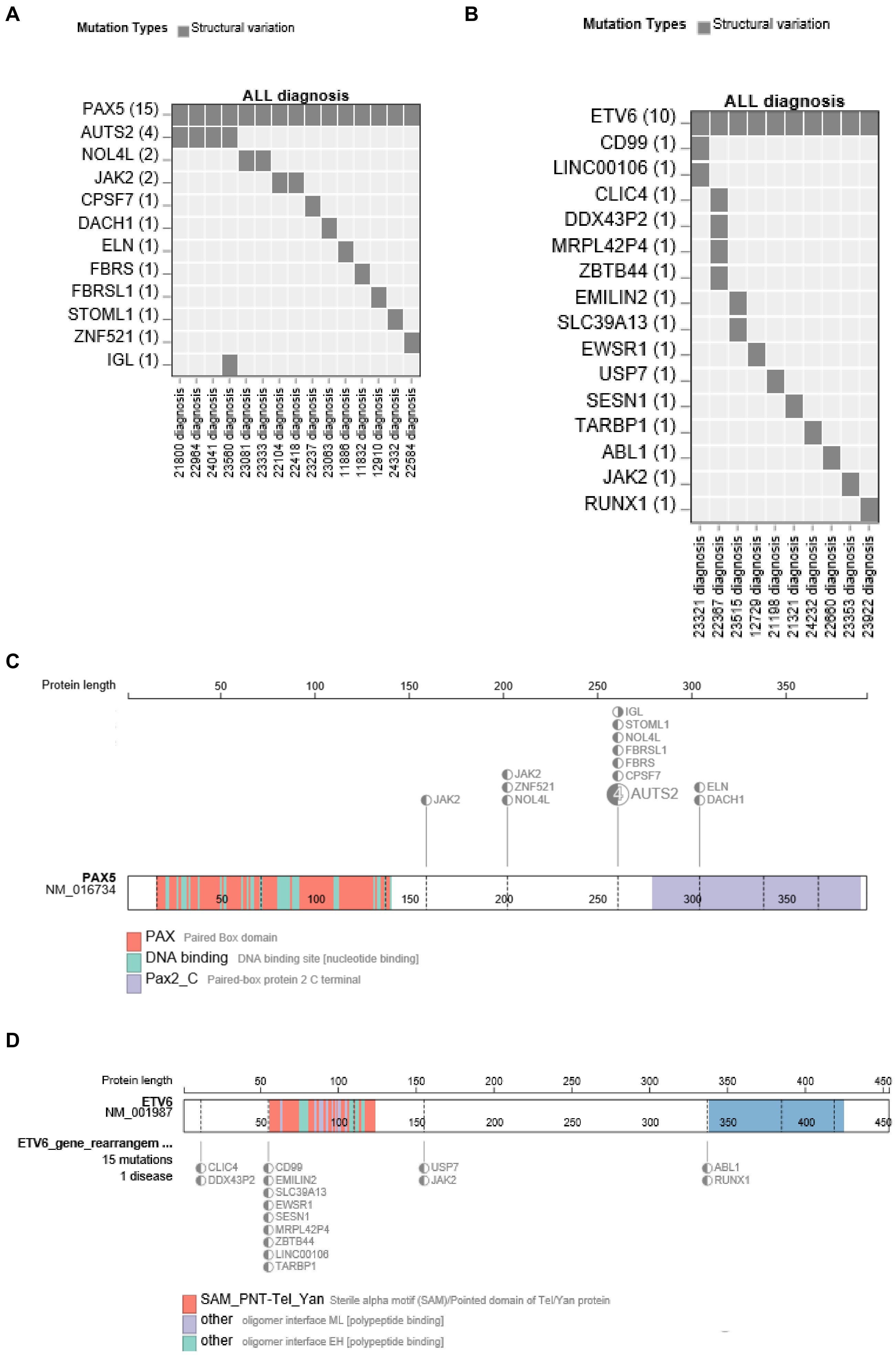

Supplementary Figure 3: *PAX5* and *ETV6* fusion genes are highly variable. Structural variants (SV) that generate fusion genes involving *PAX5* (A) or *ETV6* (B) are common but the partner gene can be highly variable. Some patients harbour multiple breakpoints that interrupt *ETV6* (23321 and 22367). Gene fusions involving *PAX5* or *ETV6* are observed in *PAX5*alt, *ETV6::RUNX1*-like, ABL-class and *JAK2*-r subtypes in this study (Supplementary Table 4). (C) Breakpoints within *PAX5* are highly variable; the coloured domains are detailed. Each lollipop stick represents a breakpoint with a single gene and the number of cases with the same genetic rearrangement is indicated. Most SV produce a fusion gene with the 5' part of *PAX5* as indicated by the grey shading of the lollipop. (D) Localisation of the breakpoints within *ETV6* for all fusion genes. Most SV produce a fusion gene with the 5' part of *ETV6,* as indicated by the grey shading of the circle. Figures were created using the ProteinPaint programme (https://proteinpaint.stjude.org).

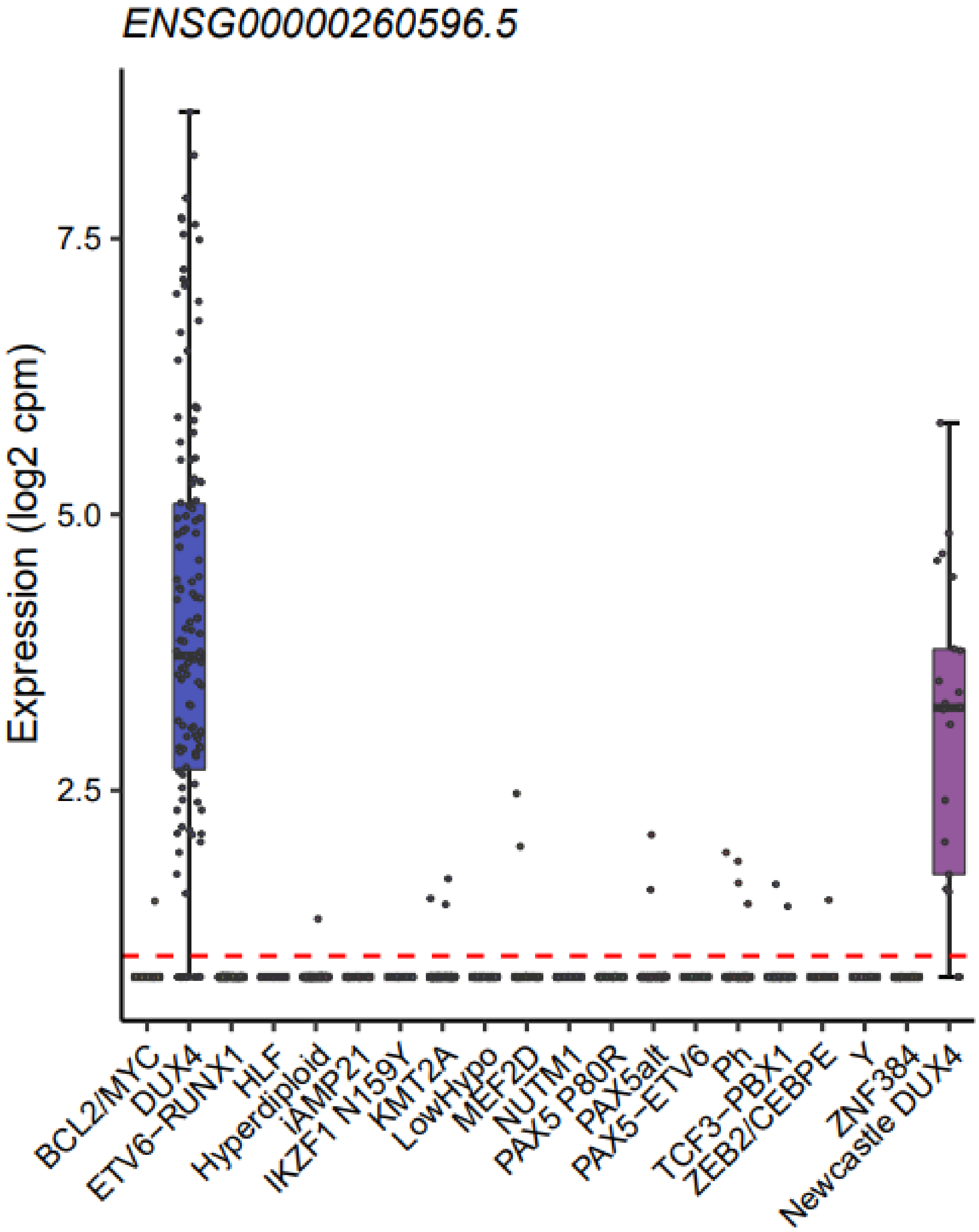

Supplementary Figure 4: *DUX4* (ENSG00000260596.5) transcript levels in B-ALL subtypes. *DUX4* transcript expression in 1473 B-ALL patients, including 1452 patients with established subtypes from our previous study[1,2] and 21 *DUX4*-r patients from this study (termed ‘Newcastle DUX4’). *DUX4* transcript expression levels are higher in patients with tSNE and PAM scores associated with *DUX4*-r B-ALL. *IGH::DUX4* is detected in 66.7% (14/21) of *DUX4*-r B-ALL cases. However, similar levels of *DUX4* transcript expression is found in other B-ALL subtypes, for example high hyperdiploidy, *KMT2A*-r and *PAX5*alt.

References

1. Gu Z, Churchman ML, Roberts KG, et al. PAX5-driven subtypes of B-progenitor acute lymphoblastic leukemia. *Nat Genet*. 2019;51(2):296-307.
2. Jeha S, Choi J, Roberts KG, et al. Clinical significance of novel subtypes of acute lymphoblastic leukemia in the context of minimal residual disease-directed therapy. *Blood Cancer Discovery*. 2021.

LP2100076-DNA_H02, C5

AACTATTGAAATTGAGTGTGAGCGGATAAGAGTGAGAGAAACAGTGGATACGTGTGGCAGTTTCTGACCAGGGTTTCTTTTTGTTT
GCAGGTGTCCAGTGTGAGGTGCAGCTGGTGGAGTCTGGGGGAGGCTTGGTACAGCCTGGGGGGTCCCTGAGACTCTCCTGTGCA
GCCTCTGGATTCACCTTCAGTTACTACTACATGAGCGGGGTCCGCCAGGCTCCCGGGAAGGGGCTGGAATGGGTAGGTTTCATTAG
AAACAAAGCTAATGGTGGGACAACAGAATAGACCACGTCTGTGAAAGGCAGATTCACAATCTCAAGAGATGATTCCAAAAGCATC
ACCTATCTGCAAATGAAGAGCCTGAAAACCGAGGACACGGCCGTGTATTACTGTTCCAGAGTCCTTATATTGTGGTGGTGACTGCTA
AATCGCGCCCTGTCTCTCCGGCCCCCGAAAGGCTGGCCATGCCGACTGTTTGCTCCCGGAGCTCTGCGGGCACCCGGAAACATGC
AGGGAAGGGTGCAAGCCCGGCATGGTGCCTTCGCTCTCCTTGCCAGGTTCCAAACCGGCCACACTGCAGACTCCCCACGTTGCCG
CACGCGGGAATCCATCGTCAGGCCATCACGCCGGGGAGGCATCTCCTCTCTGGGGTCTCGCTCTGGTCTTCTACGTGGAAATGAAC
GAGAGCCACACGCCTGCGTGTGCGAGACCGTCCCGGCAACGGCGACGCCCACAGGCATTGCCTCCTTCACGGAGAGAGGGCCTG
GCACACTCAAGACTCCCACG

Subject ID	% identity	query length	alignment length	q.start	q.end	Subject Chr	d/l	s.start	s.end
C5	100	788	402	1	402	chr14	igh	106258164	106257763
C5	100	788	21	408	428	chr14	igh	105888575	105888555
C5	100	788	349	440	788	chr4	Dux4	190070369	190070717
C5	100	788	349	440	788	chr4	Dux4	190073662	190074010
C5	100	788	349	440	788	chr4	Dux4	190076955	190077303
C5	100	788	349	440	788	chr4	Dux4	190080252	190080600
C5	100	788	349	440	788	chr4	Dux4	190083546	190083894
C5	100	788	349	440	788	chr4	Dux4	190086842	190087190
C5	100	788	349	440	788	chr4	Dux4	190090139	190090487

Supplementary Figure 5: Complex rearrangement pattern of *IGH::DUX4*. *IGH::IGH::DUX4* rearrangement in patient #21437. The contig sequence (in red, black and green text) was aligned to GRCh38, mapping to three distinct regions of the genome. The co-ordinates of the contig sequence (query start (q.start), query end (q.end)) that map to a distinct genomic region (sequence start (s.start), sequence end (s.end)) are shown in the table. In patient #21437, the contig maps to two unique regions of the *IGH* locus (chr14:106258164-106257763 (q.start-q.end; 1-402bp) and chr14:105888575-105888555 (408-428bp) and the *DUX4* locus. Due to the highly repetitive nature of the *DUX4* locus, the contig sequence (q.start-q.end; 349-440bp) maps with 100% identity to seven different genomic regions within the subtelomeric region of chromosome 4 (table, green text). Similar patterns of *IGH::DUX4* rearrangement are observed in patients #12460, #12820, #22346 and #23842. The average SRPB count for *IGH::DUX4* cases with complex rearrangements is 80.4 (range, 44.6-157.3), simillar to the average SRPB count of the entire *IGH-DUX4* cohort (n=57); 73.8.

A

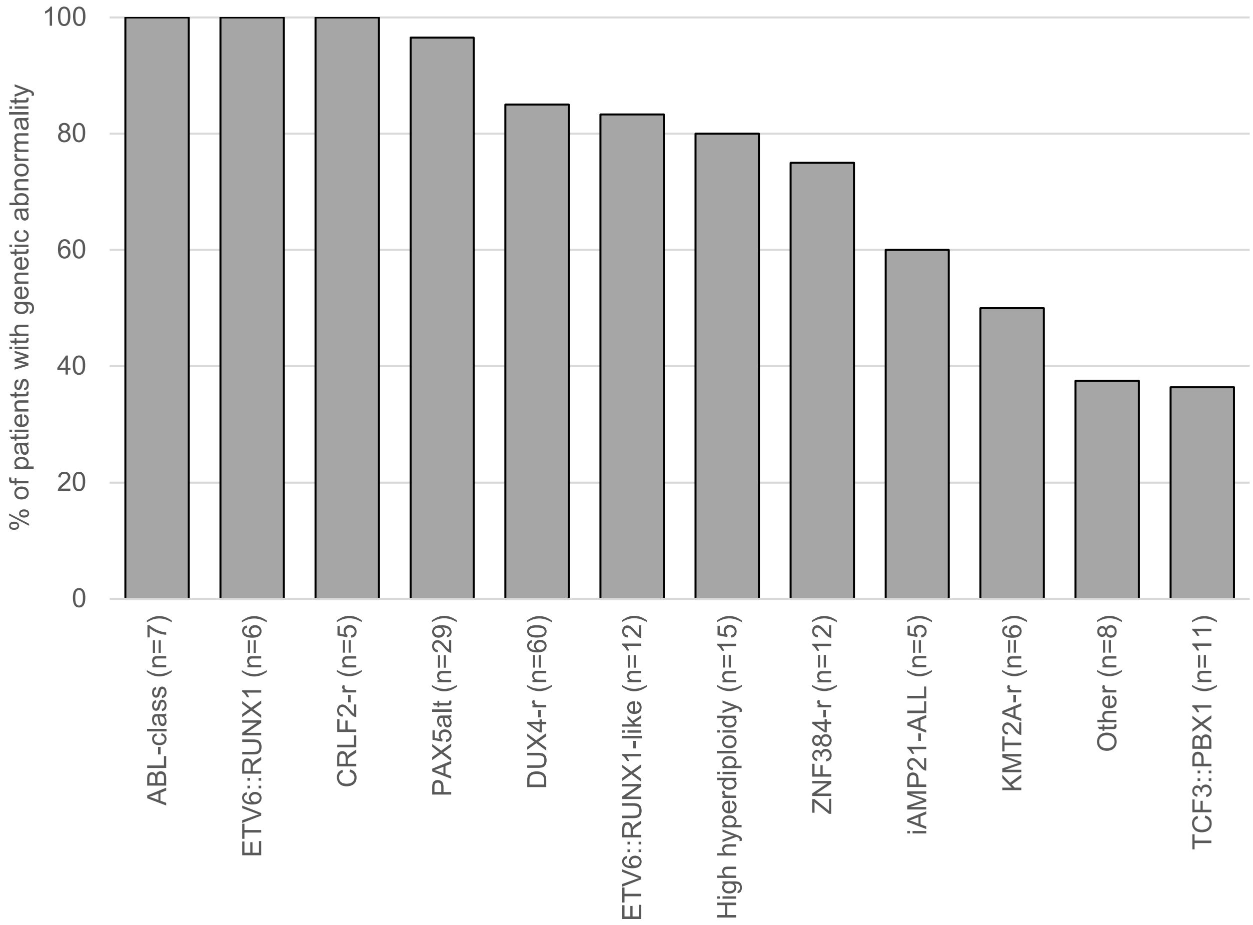
% of patients with genetic abnormality
100
80
60
40
20
0
ABL-class (n=7)
ETV6::RUNX1 (n=6)
CRLF2-r (n=5)
PAX5alt (n=29)
DUX4-r (n=60)
ETV6::RUNX1-like (n=12)
High hyperdiploidy (n=15)
ZNF384-r (n=12)
iAMP21-ALL (n=5)
KMT2A-r (n=6)
Other (n=8)
TCF3::PBX1 (n=11)

B

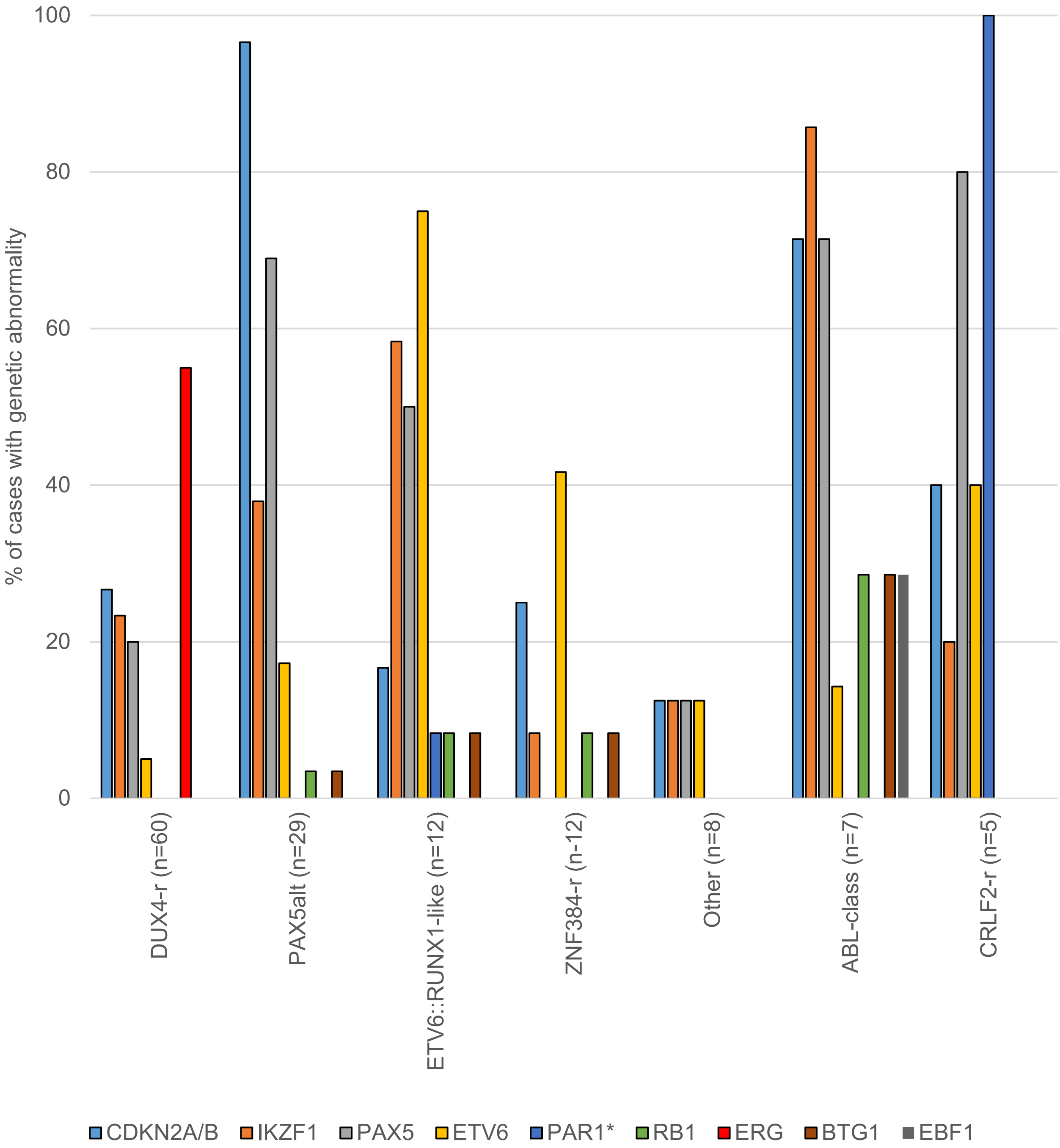
% of cases with genetic abnormality
100
80
60
40
20
0
DUX4-r (n=60)
PAX5alt (n=29)
ETV6::RUNX1-like (n=12)
ZNF384-r (n-12)
Other (n=8)
ABL-class (n=7)
CRLF2-r (n=5)
CDKN2A/B
IKZF1
PAX5
ETV6
PAR1*
RB1
ERG
BTG1
EBF1

C

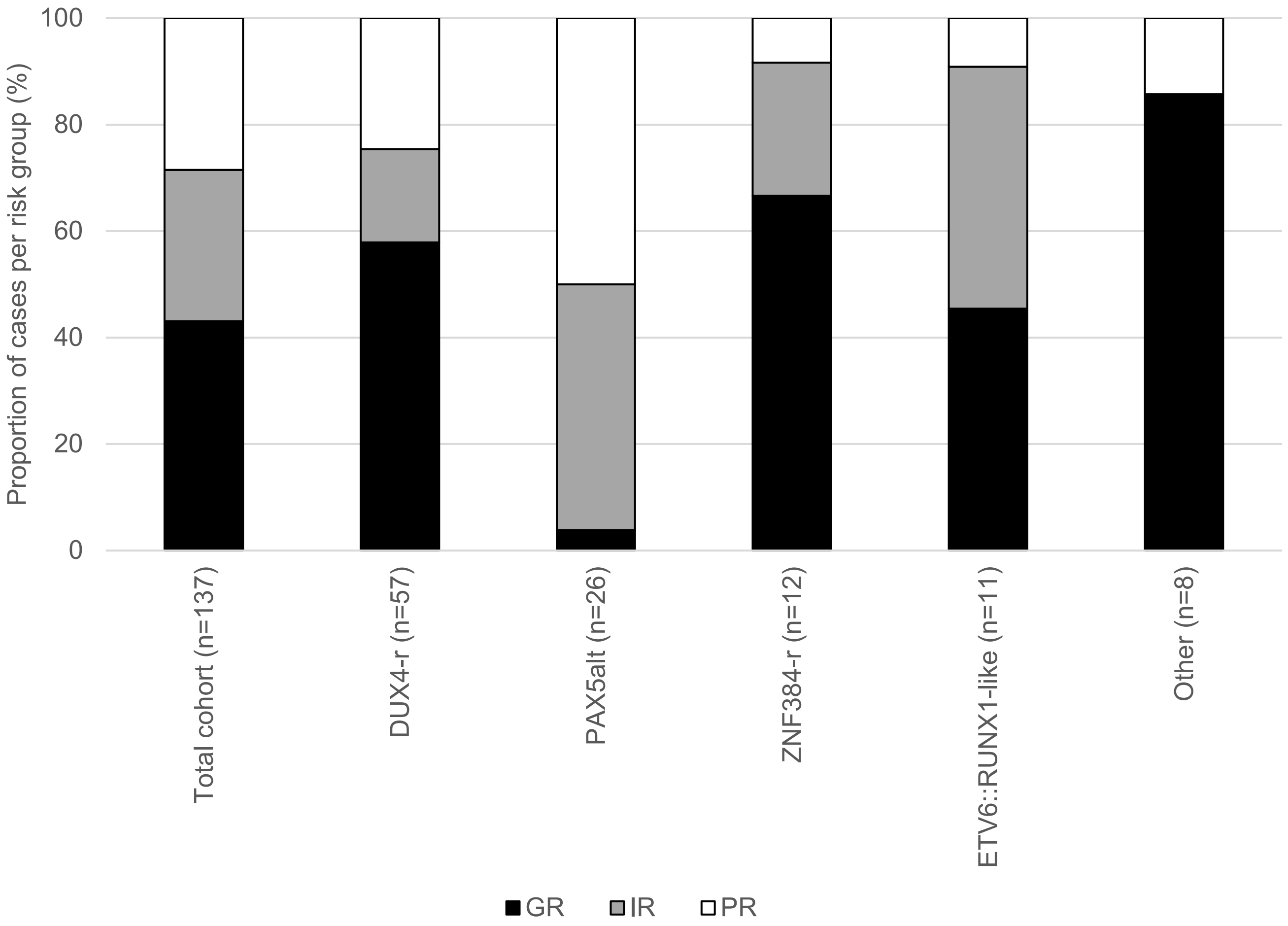

Supplementary Figure 6: Genetic abnormalities that affect nine key genes/regions in ALL. (A) Proportion of each subtype with genetic abnormalities (SV, CNA) that affect nine key genes/regions, as detected by WGS. The total number of cases per subtype is shown on the x-axis. Only subtypes with ≥5 patients were plotted. In the total cohort, 367 genetic abnormalities were identified in the nine key genes/regions, however 35 variants represent whole chromosome/arm gain, which are not relevant to the UKALL-CNA classifier.[1] (B) Of the 332 genetic abnormalities that would be employed in the UKALL-CNA classifier or involve the *ERG* gene, the proportion of cases with a genetic abnormality of each gene (*PAX5*, *IKZF1*, etc.) is represented per subtype on the x-axis. Data are plotted for subtypes that lack risk-stratifying chromosomal abnormalities (i.e. high hyperdiploidy, iAMP21-ALL) with ≥5 patients. As previously described, deletion of either *CDKN2A* or *CDKN2B* was sufficient for the locus to be classified as *CDKN2A/B* deleted.[2] Furthermore, *PAX5/IKZF1* intragenic duplications were grouped with *PAX5/IKZF1* deletions as they are predicted to be functionally equivalent. *PAR1 abnormalities specifically refer to the focal deletion that generates the *P2RY8::CRLF2* fusion. (C) Breakdown of genetic subtypes by UKALL-CNA risk group, which was previously determined by MLPA.[1] Risk group information is available for 137 patients and the number of cases per subtype is provided in the header of the x-axis. Individual risk groups are coloured: GR/good risk (black), IR/intermediate risk (grey) and PR/poor risk (white). The proportion (%) of good-, intermediate- and poor-risk cases in the total cohort and per genetic subtype is shown.

References

1. Moorman AV, Enshaei A, Schwab C, et al. A novel integrated cytogenetic and genomic classification refines risk stratification in pediatric acute lymphoblastic leukemia. *Blood*. 2014;124(9):1434-1444.
2. Schwab CJ, Chilton L, Morrison H, et al. Genes commonly deleted in childhood B-cell precursor acute lymphoblastic leukemia: association with cytogenetics and clinical features. *Haematologica*. 2013;98(7):1081-1088.

A

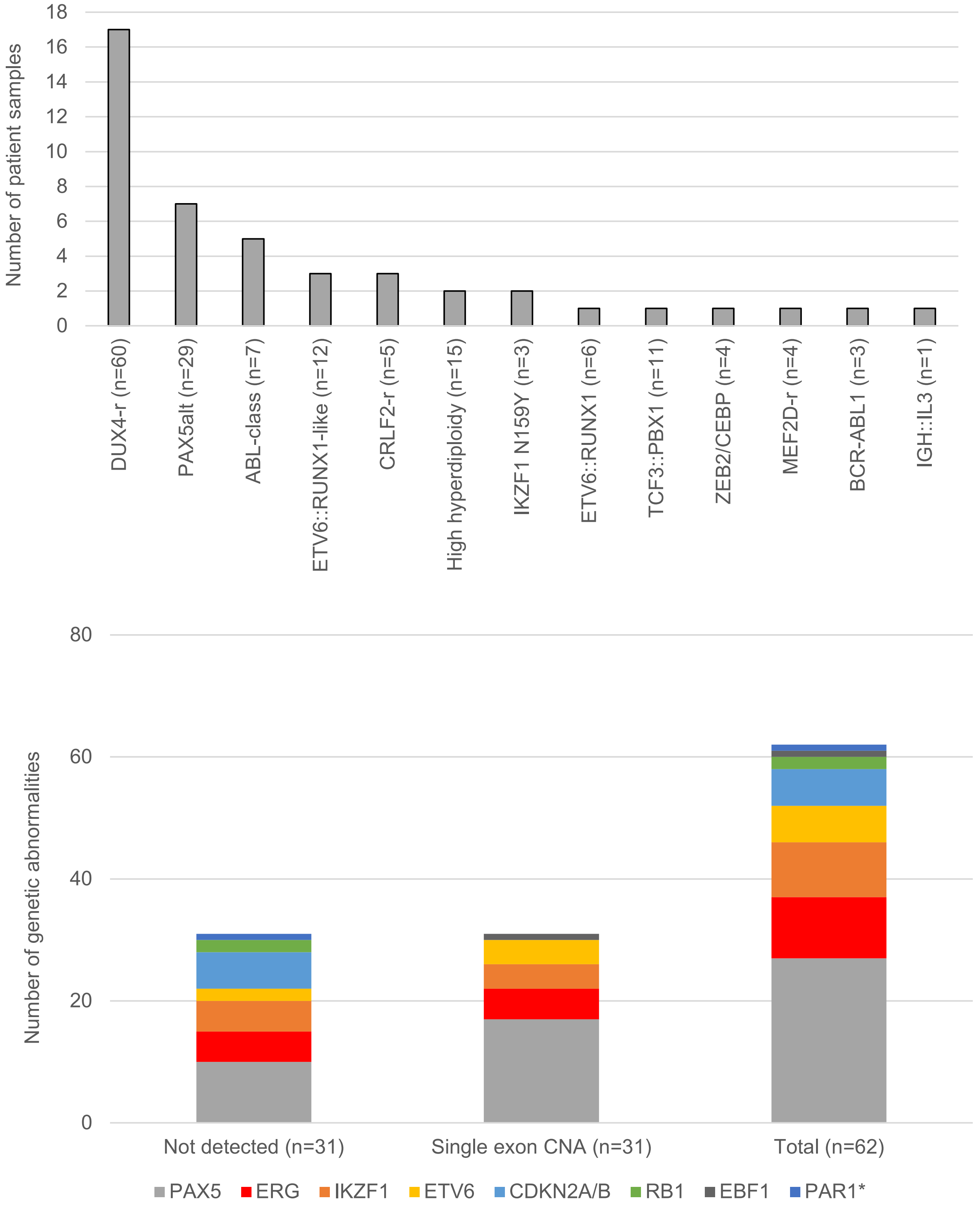

B

Supplementary Figure 7: Samples per subtype with genetic abnormalities of nine key genes/regions that were detected by WGS but not called by MLPA (A) Number of patient samples per subtype with genetic abnormalities of nine key genes/regions that were detected by WGS but not called by MLPA. They were undetected by MLPA for two main reasons: 1) single exon CNA defines genetic abnormalities that affect a single exon/region not included/included in the MLPA kit or genetic abnormalities that affect multiple exons covered by a single MLPA probe (≥two successive MLPA probes must be abnormal to call CNA by MLPA). 2) Not detected by MLPA includes genetic abnormalities that do not pass the MLPA detection threshold (≤0.75), often as a result of genetic heterogeneity. (B) The type of genetic abnormality that was undetected by MLPA and the main reason for lack of detection is shown. As previously described, deletion of either *CDKN2A* or *CDKN2B* was sufficient for the locus to be classified as *CDKN2A/B* deleted.[1] Furthermore, *PAX5/IKZF1* intragenic duplications were grouped with *PAX5/IKZF1* deletions as they are predicted to be functionally equivalent. *PAR1 abnormalities specifically refer to the focal deletion that generates the *P2RY8::CRLF2* fusion gene.

References

1. Schwab CJ, Chilton L, Morrison H, et al. Genes commonly deleted in childhood B-cell precursor acute lymphoblastic leukaemia: association with cytogenetics and clinical features. *Haematologica*. 2013;98(7):1081-1088.

Supplementary Table 1: Cohort information. The study comprises two cohorts: cohort 1 (patients 1-38) with known chromosomal abnormalities used in risk-stratification for treatment (n=38); cohort 2 (patients 39-210) negative for these abnormalities according to standard-of-care testing (Supplementary Information). Key clinical and genetic data are provided. Abnormal FISH results are described; GAIN and LOSS include centromeric probes (such as CEP4, CEP10, CEPX) and gene-specific probes (such as *RUNX1* (chromosome 21), *IGH* (chromosome 14), *CRLF2* (chromosome X)), -r=gene rearrangement. The copy number of each probe [CN=x] and % of cells containing the abnormality [x%] are provided; GAIN is CN=3 and LOSS is CN=1 unless otherwise stated. Abnormal MLPA results: copy number [CN=x] of a gene or exonic region (Ex) of a gene is provided ; GAIN is CN=3 and DEL (deletion) is CN=1 unless otherwise stated. Subgroup classification is based on the detection of primary genetic abnormalities (Table 1). Cases lacking subtype-defining genetic variants are classified as 'Other' with key (recurrent or previously associated with leukemia) genetic abnormalities listed. Some cases had genetic abnormalities that characterise ≥2 genetic subtypes of ALL, for example patient 28956 harboured a *BCR::ABL1* gene rearrangement and a pattern of chromosome gain characteristic of high hyperdiploidy. WTS subtype data is summarised and the primary subgroup of each case is provided. The blast count at diagnosis and timepoint from which the germline sample was taken is provided where available. Only 12% (25/207) of germline samples were from a timepoint before the end of induction (week 5), at which point >90% of B-ALL patients have <1% leukemic blast count in their marrow [1]. Of these, 14/25 samples had minimal residual disease (MRD) values <0.05% at the end of induction. Five samples had no result. However, six germline samples had a higher leukemic blast count, which indicatess contamination with leukaemic cells. Cases with established abnormalities found by WGS are highlighted in grey. On review of routine cytogenetic and FISH results, in some of these highlighted cases there had been indicators of specific abnormalities, including *RUNX1* (chromosome 21) gain in patients with a normal or failed karyotype that were subsequently diagnosed as high hyperdiploidy by WGS. Ph-like definition in this table indicates abnormalities that drive the unique GEP of the subtype (Ph-like) that is defined by WTS.

Cohort	Patient	Unique ID	Cytogenetic subgroup classification	Age at diagnosis (years)	Gender	Karyotype	Abnormal FISH results	Abnormal MLPA results	WGS subgroup	Other WGS subgroups	WTS subgroup	Diagnostic blast count (%)	Germline sample timepoint	Leukemic blast count in samples taken before end of induction (%)
**Cohort 1**														
1	1	28956	*BCR::ABL1*	8	M	46,XY,t(9;22)(q34.1;q11.2)[6]	*BCR::ABL1* [88%]	*IKZF1* Ex1,6,8 GAIN<br>PAR1 GAIN	*BCR::ABL1*	High Hyperdiploidy		Unknown	Week 5	No result
1	2	22722	*BCR::ABL1*	12	M	46,XX[20]	*BCR::ABL1* [54%]	*IKZF1* Ex 2-7 DEL<br>*PAX5* Ex1-6 DEL	*BCR::ABL1*			88	Day 28	>31%
1	3	10194	*BCR::ABL1*	11	M	45,XY,dic(7;9)(p1?;p1?),t(9;22)(q34;q11)[9]	*BCR::ABL1* [86%]	*CDKN2A/B* DEL<br>*IKZF1* DEL<br>*PAX5* DEL	*BCR::ABL1*			90	Week 11	NA
1	4	26681	ABL-class	5	F	47,XX,+5[3]	*EBF1::PDGFRB* [59%]	*IKZF1* Ex4-7 DEL<br>*PAX5* Ex2-5 DEL<br>RB1 Ex19-26 DEL	ABL-class			Unknown	Week 5	60%
1	5	27442	ABL-class	8	F	46,XX[20]	*EBF1::PDGFRB* [73%]	*IKZF1* Ex4-7<br>*CDKN2A/B* DEL [CN=0]<br>PAX5 DEL<br>ETV6 DEL	ABL-class			Unknown	Week 5	No result
1	6	28982	ABL-class	9	M	46,XY[20]	*EBF1::PDGFRB* [42%]	*IKZF1* Ex4-8 DEL	ABL-class			Unknown	Day 30	90%
1	7	21190	*ETV6::RUNX1*	12	F	Failed	*ETV6::RUNX1* [91%]	*CDKN2A/B* DEL [CN=0]<br>*ETV6* DEL<br>*BTG1* Ex2 DEL	*ETV6::RUNX1*			96	2 years	NA
1	8	20447	*ETV6::RUNX1*	4	F	46,XX[10]	*ETV6::RUNX1* [77%]		*ETV6::RUNX1*			98	2 years	NA
1	9	24553	*ETV6::RUNX1*	3	M	46,XY,add(12)(p11.2)[11]	*ETV6::RUNX1* [95%]		*ETV6::RUNX1*			96	3 years	NA
1	10	20755	*ETV6::RUNX1*	4	M	46,XY	*RUNX1* -r [71%]	*ETV6* DEL	*ETV6::RUNX1*			90	Remission	NA
1	11	25647	Near haploidy	13	F	53~54,XX,+X,+X,+14,+14,+18,+18,+21,+21[cp5]		PAR1 GAIN [CN=4]	Near haploidy			Unknown	Week 5	<0.05%
1	12	12202	Near haploidy	4	M	26<1n>,XY,+18,+21[5]/52,idemx2[3]		PAR1 GAIN	Near haploidy			99	Remission	NA
1	13	27162	High Hyperdipoidy	4	M	53~54,XY,+X[4],+4[3],+6[4],+10[2],+18[3],+21[3],+21[3],inc[cp4]	GAIN (CN=4): *RUNX1* [95%]	*CDKN2A/B* GAIN<br>PAR1 GAIN	High Hyperdiploidy			Unknown	Week 5	<0.05%
1	14	27809	High Hyperdipoidy	7	F	56,XX,+X,+4,+5,+6,+10,add(12)(p1?),+14,+17,+18,+21,+21[10]	GAIN: CEP4 [76%], CEP10 [76%], CEP17 [76%], CEP18 [77%]; GAIN (CN=4): *RUNX1* [100%]	PAR1 GAIN<br>*EBF1* GAIN	High Hyperdiploidy			Unknown	Week 5	<0.05%
1	15	28593	High Hyperdipoidy	3	M	46,XY[20]	GAIN (CN=3): CEP6 [72%], CEP7 [67%], CEP10 [43%], *IGH* [77%], CEP17 [67%], CEPX [82%]; GAIN (CN=4): *RUNX1* [73%]	-	High Hyperdiploidy			Unknown	Day 28	1%
1	16	28957	High Hyperdipoidy	5	M	55,XY,+X,+4,+6,add(9)(p2),add(9)(q3),+10,+14,+17,+18,+21,+21[8]	GAIN: IGH [92%]; GAIN (CN=4): *RUNX1* [91%]	*CDKN2A/B* DEL [CN=0]	High Hyperdiploidy			Unknown	Week 5	<0.05%
1	17	28959	High Hyperdipoidy	2	F	55,XX,+X,+6,+10,+14,+17,+18,+21,+21,+mar[2]	GAIN (CN=4): *RUNX1* [95%]	PAR1 GAIN	High Hyperdiploidy			Unknown	Week 5	<0.05%
1	18	29079	High Hyperdipoidy	18	M	54,XY,+X,+10,+12,+14,+17,+21,+21,+21[4]	GAIN: *HLF* [83%]; GAIN (CN=5): *RUNX1* [93%]	PAR1 GAIN<br>*ETV6* GAIN<br>*BTG1* GAIN	High Hyperdiploidy			Unknown	Week 5	<0.05%
1	19	28627	High Hyperdipoidy	1	M	55~56,XY,+X,+6,+8,+10,+14,+15,+17,+18,+21,+21,+mar[cp5]	GAIN (CN=4): *RUNX1* [27%]; GAIN (CN=5): *RUNX1* [12%]		High Hyperdiploidy			Unknown	Week 5	<0.05%
1	20	10740	High Hyperdipoidy	15	F	54-56,XX,+X,+4,+6,+8,+10,+11,+14,-16,i(17)(q10),+18,+21,+21,+1-2mar[cp7]	GAIN: *KMT2A* [79%], *IGH* [74%], *RUNX1* [10%], *CRLF2* [78%]; GAIN (CN=4): *RUNX1* [48%]; GAIN (CN=5): *RUNX1* [21%]	PAR1 GAIN	High Hyperdiploidy			99	Remission	NA
1	21	23110	High Hyperdipoidy	2	M	54,XY,+X,+6,+10,+14,t(14;14)(q11.2;q32),+17,+18,+21,+21[10]	GAIN: *IGH* [87%], *CRLF2* [85%]; GAIN (CN=4): *RUNX1* [88%]	PAR1 GAIN	High Hyperdiploidy			98	Week 11	NA
1	22	24284	Low hypodiploid	13	M	36<1n>,XY,+1,t(1;8)(p1;p1),+5,+6,+8,+9,+10,?add(10)(p1),+11,+14,?add(15)(p1),+16,+18,+19,-+21,+22 (karyotype)	LOSS: *ETV6* [73%]		Low hypodiploidy			76	Remission	NA
1	23	22340	iAMP21-ALL	10	F	46,XX,ider(21)?inv dup(21)(q1q2)[9]	AMPLIFICATION: *RUNX1* [81%]	Chr21 GENE GAIN [CN=4]<br>*BTG1* Ex2 DEL	iAMP21-ALL			100	Maintenance	NA
1	24	19578	iAMP21-ALL	11	M	48,XY,+X,?t(6;20)(p1;q1),?t(7;9)(p1;p2),i(9)(q10),+12,der(21)dup(21)(q?)[9]	AMPLIFICATION: *RUNX1* [50%]	*IKZF1* DEL<br>*CDKN2A/B* DEL [CN=0]<br>*PAX5* DEL<br>ETV6 Ex2 DEL	iAMP21-ALL			90	Follow-up	NA
1	25	20724	iAMP21-ALL	3	M	46,XY,inc[9]	AMPLIFICATION: *RUNX1* [41%]	Chr21 GENE Gain [CN=4]<br>*ERG* Ex4-12 DEL	*DUX4* -r	iAMP21-ALL	*DUX4* -r	96	Day 28	<0.05%
1	26	24390	High Hyperdiploid	3	M	51,XY,+X,+11,+14,+21,+21c[7]/47,XY,+21c[3]	GAIN: *KMT2A* [29%], *RUNX1* [58%], *IGH* [29%]; GAIN (CN=4] *RUNX1* [42%]; *IGH* -r [40%]; *CRLF2* -r [22%]	-	High Hyperdiploidy	*CRLF2* -r		88	Remission	NA
1	27	20951	*ETV6::RUNX1*	5	F	46,XX[20]	*ETV6::RUNX1* [65%]; *IGH* -r [60%]; *CDK6-r* [70%]	*PAX5* Ex1-6 DEL	*ETV6::RUNX1*			90	Remission	NA
1	28	26772	*KMT2A* -r	17	F	46,XX,ins(4;11)(q21;q13q23)[7]	*KMT2A* -r [82%]		*KMT2A* -r			Unknown	Week 5	No result
1	29	21318	*KMT2A* -r	14	M	46,XY,t(4;11)(q21;q23)[3]/46,idem,del(6)(q21)[4]	*KMT2A* -r [91%]		*KMT2A* -r			96	Week 49	NA
1	30	21940	*KMT2A* -r	21	F	46,XX,t(4;11)(q21;q23),del(9)(q1q3)[20]/50,XX,t(4;11)(q21;q23),+6,+13,-15,+22,+2mar[3]	*KMT2A* -r [52%]; *IGH* -r [13%]	*CDKN2A/B* DEL	*KMT2A* -r			95	Day 28	5.70%
1	31	23003	*KMT2A* -r	1	M	46,XY,t(4;11)(q21;q23)[10]		*IKZF1* Ex2-7 DEL	*KMT2A* -r			85	Week 49	NA
1	32	22368	*KMT2A* -r	16	M	Failed	*KMT2A* -r [70%]	*IKZF1* Ex2-7 DEL	*KMT2A* -r			99	Week 23	NA
1	33	25379	*KMT2A* -r	41	F	46,XX,t(4;11)(q21;q23)[10]	*KMT2A* -r [92%]	*CDKN2A/B* DEL [CN=0]	*KMT2A* -r			92	Remission	NA
1	34	21708	Near haploidy	6	M	26<1n>,XY,+14,+21[8]/52,idemx2[2]	LOSS: *BCR* [95%], *ABL1* [95%], *KMT2A* [95%], *RUNX1* [89%], *TCF3* [94%]	PAR1 GAIN [CN=4]	Near haploidy			98	Remission	NA
1	35	7653	Near haploidy	15	M	28<1n>,X,+Y,+8,+10,+18,+21[10]	LOSS: *BCR* [89%], *ABL1* [89%], IGH [89%], *KMT2A* [91%], *CDKN2A/B* [94%], *ETV6* [96%]		Near haploidy			96	Remission	NA
1	36	27354	*TCF3::PBX1*	8	M	46,XY,der(19)t(1;19)(q23;p13.3)[6]			*TCF3::PBX1*			Unknown	Week 5	<0.05%
1	37	27397	*TCF3::PBX1*	14	M	46,XY,der(19)t(1;19)(q23;p13.3),inc[2]	*TCF3* -r [91%]	*CDKN2A/B* DEL<br>*PAX5* DEL	*TCF3::PBX1*			Unknown	Week 5	<0.05%
1	38	27304	*TCF3::PBX1*	15	M	46,XY,der(19)t(1;19)(q23;p13.3)[9]	*TCF3* -r [95%]	-	*TCF3::PBX1*			Unknown	Unknown	NA
Cohort 2														
2	39	8451	Other	7	M	42~45,XY,-9,-13,+mar[cp6]	LOSS: *ABL1* [32%], *CDKN2A/B* [52%]	*CDKN2A/B* DEL [CN=0]<br>*PAX5* DEL<br>*RB1* DEL	*MEF2D-* r		*MEF2D-* r	100	Post D1	NA
2	40	8878	Other	8	F	42,X,-X,-3,add(4)(p1),add(8)(q2),del(12)(p1),-14,-16,-19,+mar[1]	LOSS: *ETV6* [66%]	*ETV6* DEL	*ZNF384* -r		*ZNF384* -r	70	EOT	NA
2	41	9051	Other	15	M	46,XY[40]	*MYC* -r [16%]	-	*IKZF1* N159Y		*IKZF1* N159Y	85	EOT	NA
2	42	9469	Other	11	M	46,XY[20]	*IGH* -r [33%]	*IKZF1* Ex4-7 DEL<br>*CDKN2B* Ex2 DEL<br>*ERG* Ex5-6 DEL	*DUX4* -r			71	Day 28	<0.05%
2	43	9850	Failed	10	M	46,XY[12]		*CDKN2A/B* DEL<br>*CDKN2A* Ex2 DEL [CN=0]<br>*IKZF1* Ex1-7 DEL	ABL-class	*MEF2D* -r		90	EOT	NA
2	44	10062	Other	3	M	Failed	GAIN: *RUNX1* [51%]	*IKZF1* Ex4-7 DEL<br>*CDKN2A/B* DEL [CN=0]<br>*PAX5* DEL	*PAX5* alt		*PAX5* alt	70	EOT	NA
2	45	10184	Other	9	M	46,XY[30]	*ZNF384* -r [12%]	*BTG1* Ex2 DEL	*ZNF384* -r		*ZNF384* -r	85	EOT	NA
2	46	10186	Other	9	F	47,XX,+8[3]		-	*DUX4* -r			89	Maintenance	NA
2	47	10310	Other	12	M	46,XY[20]		-	*DUX4* -r		*DUX4* -r	90	EOT	NA
2	48	10442	Other	12	M	46,XY[3]	GAIN: *RUNX1* [87%], *PDGFRB* [84%]; LOSS: *ETV6* [87%]	Chr21 GENE GAIN [CN=5]<br>*IKZF1* DEL<br>*ETV6* DEL<br>PAX5 Ex1-6 GAIN	iAMP21-ALL		iAMP21-ALL	93	Maintenance	NA
2	49	10656	Other	3	M	45,XY,der(7)t(7;9)(q?11.2;q?12),-9,der(20)t(7;20)(q?11.2;q?11.2)[5]/45,XY,idem,del(13)(q?12q?14)[6]	LOSS: *JAK2* [69%], *PAX5* [56%]	*PAX5* DEL<br>*CDKN2A/B* DEL<br>*IKZF1* DEL	*PAX* 5alt			99	EOT	NA

2	50	10868	Other	2	F	48,XX,+X,dic(9;20)(p11~13;q11.21),+10,?del(15)(q2?2),+21[7]	GAIN: *RUNX1* [45%]; DELETION: 9p13.2 [96%], 20q11.21 [96%]	*PAX5* DEL<br>*CDKN2A* DEL [CN=0]<br>*CDKN2B* DEL<br>Chr21 GENE GAIN<br>PAR1 GAIN	*Other*	*CNTNAP3B* abnorm	Other	80	EOT	NA
2	51	10876	Other	9	M	46,XY[20]		*CDKN2A/B* DEL [CN=0]	*DUX4*-r		*DUX4*-r	85	EOT	NA
2	52	10925	Other	9	F	46,XX[20]		PAR1 DEL	*DUX4*-r			90	EOT	NA
2	53	10927	Other	5	M	46,XY[26]	GAIN: *BCR* [29%], *RUNX1* [19%], *CRLF2* [17%], CEP6 [30%]	Chr21 GENE GAIN<br>PAR1 GAIN<br>*ETV6* Ex2-5 DEL	*ETV6::RUNX1*-like			90	EOT	NA
2	54	11053	Other	16	F	Failed		Chr21 GENE GAIN	*DUX4*-r			Unknown	EOT	NA
2	55	11060	Other	14	M	46,XY[20]	GAIN: *KMT2A* [22%]	*EBF1* GAIN<br>*ETV6* GAIN<br>Chr21 GENE GAIN<br>PAR1 GAIN	Low hypodiploidy			99	EOT	NA
2	56	11062	Other	6	M	46,XY,der(2)t(2;8)(?p21;?q13),-8,-8,-13,-14,add(14)(q32),add(19)(p13.3),+4mar[4]			*BCL2/MYC*			89	PRED1	NA
2	57	11148	Failed	10	F	46,XX[16]		-	*DUX4*-r			Unknown	EOT	NA
2	58	11149	Failed	3	F	Failed	LOSS: *ETV6* [18%]	*ETV6* DEL	Other	*SPOP* abnormality		61	PREDI-1	NA
2	59	11178	Failed	15	M	46,XY	LOSS: *ETV6* [70%]	*ETV6* DEL<br>*ETV6* Ex2 DEL [CN=0]	*DUX4*-r			Unknown	EOT	NA
2	60	11440	Failed	5	M	Failed	*TCF3*-r [25%]; GAIN: *ABL2* [65%]	-	*TCF3::PBX1*		*TCF3::PBX1*	92	PREDI-1	NA
2	61	11553	Failed	13	F	Failed	LOSS: *PAX5* [83%]; GAIN: *ABL2* [78%]	*CDKN2A/B* DEL<br>*PAX5* DEL	*TCF3::PBX1*			95	EOT	NA
2	62	11556	Other	4	F	47,XX,+21[4]	GAIN: *RUNX1* [23%]	*ERG* Ex5-11 DEL<br>*IKZF1* Ex4-7 DEL<br>*RUNX1* GAIN	*DUX4*-r			Unknown	Remission	NA
2	63	11672	Other	8	M	46,XY,?add(3)(q2?),?del(5)(p?),?del(6)(q?),inc[5]		*ERG* Ex4-7 DEL<br>*IKZF1* Ex4-7 DEL	*DUX4*-r		*DUX4*-r	100	PREDI-1	NA
2	64	11734	Other	8	F	47,XX,+21c[20]	GAIN: *RUNX1* [91%]; *CRLF2*-r [31%], *P2RY8*-r [64%]	Chr21 GENE GAIN<br>PAR1 DEL<br>*ETV6* Ex1 GAIN	*CRLF2*-r			99	PRED1	NA
2	65	11811	Failed	4	F	46,XX[10]	LOSS: *CDKN2A/B* [9%]	*CDKN2A/B* DEL	*DUX4*-r			96	EOT	NA
2	66	11832	Other	2	F	45,XX,dic(9;16)(p11;p11),add(15)(q2?6)[9]	LOSS: *CDKN2A/B* [90%], *JAK2* [73%], *PAX5* [74%], *NTRK3* [67%]	*CDKN2A/B* DEL [CN=0]<br>*PAX5* Ex8-10 DEL	*PAX5* alt			Unknown	Maintenance	NA
2	67	11886	Other	1	M	46,XY,r(7)(p22q11.2),der(9)t(7;9)(q11.2;p13)[12]	LOSS: *JAK2* [68%], *PAX5* [58%], *IKZF1* [11%]	*CDKN2A/B* DEL [CN=0]<br>*ETV6* Ex1 DEL	*PAX5* alt		*PAX5* alt	97	EOT	NA
2	68	11957	Other	6	M	46,XY[20]		*ERG* Ex5-10 DEL	*DUX4*-r			98	EOT	NA
2	69	11959	Other	19	M	46,XY[20]		*EBF1* Ex16 DEL	ABL-class			99	EOT	NA
2	70	12083	Other	15	M	46,XY[17]	GAIN : *RUNX1* [27%]; LOSS: *PAX5* [17%]	*ERG* Ex5-9 DEL	*DUX4*-r			96	EOT	NA
2	71	12118	Other	4	F	46,XX[18]		*ERG* Ex4-12 DEL	*DUX4*-r			91	EOT	NA
2	72	12134	Other	13	M	47,XY,+X[13]		PAR1 GAIN<br>*CDKN2A/B* DEL [CN=0]<br>*ERG* Ex5-9 DEL	*DUX4*-r		*DUX4*-r	95	Maintenance	NA
2	73	12334	Other	15	M	46,XY[20]		*ERG* Ex5-9 DEL	*DUX4*-r			96	EOT	NA
2	74	12335	Other	7	M	46,XY[20]	*IGH*-r [89%]; *IGF2*-r[83%]	-	*DUX4*-r			95	Follow-up	NA
2	75	12356	Other	5	M	46,XY,i(21)(q10)[2]	GAIN: *RUNX1* [58%]	Chr21 GENE GAIN	*DUX4*-r	iAMP21-ALL	*DUX4*-r	81	EOT	NA
2	76	12373	Other	4	M	46,XY[11]	GAIN: *RUNX1* [59%], *CRLF2* [61%], *IGH* [74%]	*CDKN2A/B* DEL [CN=0]<br>Chr21 GENE GAIN [CN=4]<br>PAR1 GAIN	High Hyperdiploidy		High Hyperdiploidy	95	EOT	NA
2	77	12460	Failed	12	M	Failed		*ETV6* Ex1 DEL	*DUX4*-r			90	EOT	NA
2	78	12522	Other	11	M	46,XY,t(2;4)(q35;q21),del(9)(p21),inv(9)(p24q11)[2]/47,idem,+der(9)del(9)(p13)del(9)(q13)[3]		*CDKN2A/B* DEL<br>*PAX5* DEL	*PAX5* alt		*PAX5* alt	91	PREDI-1	NA
2	79	12641	Other	19	M	46,XY,del(6)(q13q2?7),der(9)t(9;9)(p2;q3),add(19)(p13)[14]		*CDKN2A/B* DEL<br>*PAX5* DEL	*TCF3::PBX1*		*TCF3::PBX1*	99	EOT	NA
2	80	12729	Other	1	F	42~46,XX,del(1)(q3q4),add(16)(p13),+2mar,inc[cp16]		-	*ETV6::RUNX1*-like		*ETV6::RUNX1*-like	98	PREDI-1	NA
2	81	12816	Other	4	M	46,XY[20]		*ERG* Ex5-11 DEL	*DUX4*-r		*DUX4*-r	94	EOT	NA
2	82	12820	Other	1	M	46,XY[20]		*ERG* Ex5-9 DEL	*DUX4*-r			98	EOT	NA
2	83	12908	Other	18	M	46,XY,t(8;17)(?q10;?q10),del(9)(p11)[8]	LOSS: *PAX5* [86%], *JAK2* [83%], *CDKN2A/B* [13%]	*PAX5* DEL<br>*CDKN2A/B* DEL [CN=0]	*PAX5* P80R		*PAX5* P80R	96	NA	NA
2	84	12910	Other	1	M	46,XY,del(9)(p12)[10]	LOSS: *CDKN2A/B* [90%]	*CDKN2A/B* DEL [CN=0]<br>*PAX5* Ex8-10 DEL	*PAX5* alt		*PAX5* alt	95	EOT	NA
2	85	12911	Other	15	M	46,XY[20]	LOSS: *ETV6* [82%]	*ETV6* DEL	*MEF2D*-r		*MEF2D*-r	99	EOT	NA
2	86	19730	Other	1	F	46,XX[20]		-	*TCF3::PBX1*			81	EOT	NA
2	87	19732	Other	1	M	45,XY,-7,der(9)t(7;9)(p15;p22),t(11;12)(q13;q24),add(19)(q13.4),+mar[10]		*CDKN2A* DEL [CN=0]<br>*CDKN2B* DEL<br>*IKZF1* Ex2-7 DEL<br>PAX5 DEL	*PAX5* alt		*PAX5* alt	99	EOT	NA
2	88	19827	Other	6	M	46,XY[20]		*ERG* Ex5-11 DEL	*DUX4*-r			100	Maintenance	NA
2	89	20035	Other	14	M	46,XY,t(5;9)(q33.3;q23.3)c		*ERG* Ex5-9 DEL<br>*IKZF1* Ex2-7 DEL	*DUX4*-r		*DUX4*-r	100	EOT	NA
2	90	20161	Other	15	M	46,XY,+8,-20[17]/49,idem,+6,+12,+20[3]	LOSS: *CDKN2A/B* [91%]	*CDKN2A/B* DEL [CN=0]	*PAX5* alt			98	Maintenance	NA
2	91	20315	Failed	3	F	Failed	GAIN: *ABL2* [42%]		*TCF3::PBX1*	*PAX5* alt		5	EOT	NA
2	92	20317	Other	13	M	46,XY[20]		*CDKN2A/B* DEL [CN=0]	*ZEB2/CEBP*		*ZEB2/CEBPE*	80	Maintenance	NA
2	93	20323	Other	19	M	46,XY[10]	LOSS: *ETV6* [20%]	*ETV6* Ex2-8 DEL	*ZNF384*-r		*ZNF384*-r	70	Maintenance	NA
2	94	20515	Other	2	F	46,XX[20]			*DUX4*-r		*DUX4*-r	60	EOT	NA
2	95	20628	Failed	16	F	Failed	*PDGFRB*-r [89%]; LOSS: *PAX5* [55%]	*IKZF1* Ex2-3 DEL<br>*CDKN2A/B* DEL [CN=0]<br>*PAX5* DEL<br>BTG1 DEL	ABL-class		Ph-like	95	EOT	NA
2	96	20653	Failed	14	F	46,XX[16]	LOSS: *CDKN2A/B* [12%]	*CDKN2A/B* DEL [CN=0]	*ZNF384*-r			100	EOT	NA
2	97	20683	Other	8	M	46,XY[20]			*DUX4*-r		*DUX4*-r	92	Maintenance	NA
2	98	20696	Failed	16	F	Failed	GAIN: *CRLF2* [15%]	*CDKN2A/B* DEL	*DUX4*-r		*DUX4*-r	90	EOT	NA
2	99	20706	Other	3	M	49,XY,+X,del(9)(p22),+11,der(?18)t(17;18)(q11.2;p11.2),+21[8]	GAIN: *RUNX1* [79%], *CRLF2* [38%]	*CDKN2A/B* DEL<br>*PAX5* DEL	*PAX5* alt		*PAX5* alt	40	EOT	NA
2	100	20716	Other	5	M	46,XY[20]			*DUX4*-r		*DUX4*-r	98	EOT	NA
2	101	20720	Other	16	M	46,XY,add(4)(p1?)[6]		*ERG* Ex5-9 DEL	*DUX4*-r			98	Unknown	NA
2	102	20756	Other	14	M	46,XY,del(9)(p21)[8]	LOSS: *CDKN2A/B* [92%], *PAX5* [90%]		*PAX5* P80R			Unknown	EOT	NA
2	103	20764	Other	7	M	46,XY,add(13)(p1),i(21)(q10)[4]/47,idem,+i(21)(q10)[6]	GAIN: *RUNX1* [80%]	*ETV6* Ex2-5 DEL<br>Chr21 GENE GAIN [CN=5]	*ETV6::RUNX1*-like			100	EOT	NA
2	104	20874	Other	16	M	46,XY,i(9)(q10)[8]/46,XY,del(9)(p10)[2]	LOSS: *PAX5* [28%], *CDKN2A/B* [20%]; GAIN: *BCR* [42%]	*PAX5* DEL<br>*CDKN2A/B* DEL<br>*CDKN2A* Ex4 DEL [CN=0]	*PAX5* P80R		*PAX5* P80R	100	Maintenance	NA
2	105	21198	Other	3	M	46,XY[20]	LOSS: *ABL2* [81%]	*ETV6* Ex2-3 DEL [CN=0]<br>*ETV6* Ex5-8 DEL	*ETV6::RUNX1*-like		*ETV6::RUNX1*-like	98	EOT	NA
2	106	21230	Failed	21	M	46,XY[13]		*ERG* Ex5-12 DEL<br>*IKZF1* Ex4-7 DEL	*DUX4*-r			100	PREDI-1	NA
2	107	21321	Other	3	M	47,XY,del(6)(q21),+10,add(12)(p13),del(12)(q1?5),add(22)(q13)[4]		*PAX5* Ex2-10 DEL<br>*ETV6* Ex1-3 DEL<br>PAR1 DEL	*ETV6::RUNX1*-like	*CRLF2*-r	*ETV6::RUNX1*-like	83	Maintenance	NA

2	108	21322	Other	2	M	46,XY,-2,der(10)?t(2;10)(q2?1;q2?2),+der(14)[3]			*DUX4*-r			95	Maintenance	NA
2	109	21342	Other	4	M	47,Y,add(X)(q2?8),+21c/47,XY,+21c[5]	GAIN: *RUNX1* [100%]; *CRLF2*-r [59%], *P2RY8*-r [57%]	Chr21 GENE GAIN<br>PAR1 DEL	*CRLF2*-r		Ph-like	95	PREDI-1	NA
2	110	21424	Other	4	M	46,XY,del(9)(p22),inc[4]			Other	*MAPK10*-r		30	Maintenance	NA
2	111	21437	Failed	11	M	46,XY[16]			*DUX4*-r			95	Maintenance	NA
2	112	21466	Other	6	F	44,XX,-7,dic(7;11)(q22;p13),dic(9;15)(p11;p11),add(11)(p14),-20,der(?)(?;1)(?;q1),+mar[12]	LOSS: *JAK2* [37%], *PAX5* [34%]; GAIN: *MEF2D* [34%]	*PAX5* DEL<br>*CDKN2A/B* DEL<br>*IKZF1* DEL	*ETV6::RUNX1*-like	Ph-like	Other	55	Maintenance	NA
2	113	21487	Other	2	F	48,XX,+8,+21[2]/47,idem,-8[3]/48,idem,ins(1;?)(p2;?)[5]/48,idem,add(15)(q2)[2]	GAIN: *RUNX1* [81%]	Chr21 GENE GAIN	*IKZF1* N159Y	*PAX5* alt		Unknown	EOT	NA
2	114	21532	Other	13	F	46,XX,del(9)(p13p22)[6]		*BTG1* GAIN<br>*EBF1* GAIN<br>*ETV6* GAIN<br>PAX5 DEL<br>CDKN2A/B DEL [CN=0]	*NUTM1*-r			94	PREDI-1	NA
2	115	21568	Failed	7	F	46,XX[4]		*IKZF1* Ex4-7 DEL	*DUX4*-r		*DUX4*-r	96	Maintenance	NA
2	116	21689	Other	9	M	47,XY,t(17;21)(q22;q22),+der(21)t(17;21)[10]	GAIN: *RUNX1* [58%]	Chr21 GENE GAIN	*DUX4*-r			95	Maintenance	NA
2	117	21693	Other	18	F	Failed	GAIN: *ABL1* [62%]; LOSS: *ETV6* [71%]	*CDKN2A/B* DEL [CN=0]<br>*PAX5* DEL<br>*ETV6* DEL	*PAX5* alt		*PAX5* alt	Unknown	Maintenance	NA
2	118	21694	Other	13	M	46,XY[9]	GAIN: *RUNX1* [4%]		*IKZF1* N159Y			90	Follow-up	NA
2	119	21795	Other	8	F	46,XX[20]	GAIN: *RUNX1* [58%], *CRLF2* [21%]	Chr21 GENE GAIN [CN=4]	iAMP21-ALL		iAMP21-ALL	69	Maintenance	NA
2	120	21800	Other	4	M	44,X,-Y,del(6)(q21q23),-7,add(9)(p2?),der(12),t(12;?13)(p1?3;p11),-13,[cp9]	LOSS: *ETV6* [51%], *JAK2* [65%], *ZNF384* [10%]	*ETV6* DEL<br>*CDKN2A/B* DEL [CN=0]<br>PAX5 Ex7-10 DEL	*PAX5* alt			85	PREDI-1	NA
2	121	21869	Other	3	M	47,XY,+21c[21]	GAIN: *RUNX1* [100%]	Chr21 GENE GAIN<br>*CDKN2A/B* DEL<br>*ETV6* Ex1 DEL<br>PAX5 Ex2-8 DEL<br>PAR1 DEL	*CRLF2*-r		Ph-like	95	Maintenance	NA
2	122	22006	Other	21	M	46,XY[20]	LOSS: *CDKN2A/B* [22%]		*DUX4*-r		*DUX4*-r	99	Unknown	NA
2	123	22037	Other	2	F	46,XX[20]		*ERG* Ex5-11 DEL	*DUX4*-r		*DUX4*-r	89	EOT	NA
2	124	22045	Other	2	M	46,XY,?del(17)(p?)[5]			*DUX4*-r		*DUX4*-r	96	Maintenance	NA
2	125	22046	Other	3	M	47,XY,+21c[20]	GAIN (CN=4): *RUNX1* [50%]	Chr21 GENE GAIN [CN=4]<br>Chr13 GENE DEL	*ZEB2/CEBP*		Other	99	Maintenance	NA
2	126	22065	Other	11	M	46,XY[20]		*CDKN2B* DEL	*DUX4*-r			99	Maintenance	NA
2	127	22094	Other	10	F	46,XX[20]	*ZNF384*-r [18%]; LOSS: *ETV6* [51%]	*ETV6* DEL	*ZNF384*-r			92	EOT	NA
2	128	22104	Other	1	M	46,XY[20]	*PAX5*-r [60%], *JAK2*-r [47%]		*JAK2*-r			97	PREDI-1	NA
2	129	22150	Other	19	M	43~45,XY,?dic(7;9)(p1;p1),+1~2mar[cp11]	LOSS: *CDKN2A/B* [90%], *PAX5* [90%], *JAK2* [93%]	*CDKN2A/B* DEL [CN=0]<br>*PAX5* DEL<br>*IKZF1* DEL	*PAX5* P80R		*PAX5* P80R	90	Maintenance	NA
2	130	22153	Other	4	F	46,XX[20]		*CDKN2A/B* DEL	*PAX5* alt			65	EOT	NA
2	131	22188	Other	2	F	46,XX,-12,+mar[1]/46,idem,?del(9)(?q1?q3)[2]			Other	*CNTNAP3B* abnorm	Other	95	Day 28	No result
2	132	22224	Other	18	M	Failed	*IGH*-r [66%]; LOSS: *RUNX1* [99%]	*ETV6* DEL	*DUX4*-r			Unknown	Follow-up	NA
2	133	22245	Other	12	F	46,XX,inc[16]	-		*TCF3::PBX1*			97	EOT	NA
2	134	22327	Other	2	M	Failed	GAIN: CEP4 [85%]; GAIN (CN=4): *RUNX1* [86%]	Chr21 GENE GAIN [CN=4]	High Hyperdiploidy		High Hyperdiploidy	99	Maintenance	NA
2	135	22346	Other	4	M	46,XY[20]		*ERG* Ex6 DEL	*DUX4*-r			90	Maintenance	NA
2	136	22352	Other	10	M	46,XY[22]		*IKZF1* Ex2-3 DEL	*PAX5* P80R		*PAX5* P80R	99	NA	NA
2	137	22354	Other	14	F	46,XX,add(3)(p25)[7]		*CDKN2A* DEL<br>*CDKN2B* DEL [CN=0]	*DUX4*-r			90	Maintenance	NA
2	138	22355	Other	6	F	46,XX,t(8;14)(q11;q32)[8]	*IGH*-r [100%]; *CRLF2*- r [9%]; *CEBPD*-r [77%]		*DUX4*-r	*ZEB2/CEBP*		90	Day 28	<0.05%
2	139	22367	Failed	2	F	Failed	GAIN: *RUNX1* [63%]		*ETV6::RUNX1*- like			99	EOT	NA
2	140	22387	Other	10	F	46,XX[20]		*CDKN2A/B* DEL [CN=0]<br>*ERG* Ex5-9 DEL	*DUX4*-r		*DUX4*-r	97	EOT	NA
2	141	22405	Other	2	M	46,XY[20]		*ERG* Ex5-9 DEL	*DUX4*-r			99	Unknown	NA
2	142	22416	Failed	2	M	Failed	GAIN: *RUNX1* [83%], *IGH* [61%], *ETV6* [23%]	*PAX5* Ex2-10 DEL<br>*CDKN2A/B* DEL<br>Chr21 GENE GAIN [CN=4]	High Hyperdiploidy			90	Maintenance	NA
2	143	22417	Other	15	M	46,XY[20]	*IGH*- r [74%]	*IKZF1* Ex4-7 DEL<br>*CDKN2A/B* DEL<br>*ERG* Ex5-9 DEL	*DUX4*-r			97	Remission	NA
2	144	22418	Other	3	F	46,X,-X,t(3;11)(q25;q13),-7,-20,+3mar[4]/45,idem,del(12)(p11.2),-mar[4]	LOSS: *ETV6* [10%]	PAR1 DEL<br>*IKZF1* DEL	*JAK2*-r		Ph-like	90	Maintenance	NA
2	145	22466	Other	2	M	44~45,XY,der(1;12)(p10;q10),?ins(7;?)(p13p15;?),+mar[cp3]	LOSS: *ETV6* [74%], *TCF3* [24%]; GAIN: *KMT2A* [10%]	*ETV6* DEL<br>*IKZF1* DEL<br>*RB1* DEL	*ETV6::RUNX1*-like		*ETV6::RUNX1*-like	91	Week 11	NA
2	146	22572	Other	3	F	46,XX[20]	-	*CDKN2A/B* DEL	*IGH::IL3*		Ph-like	16	EOT	NA
2	147	22584	Other	1	M	ND	LOSS: *ABL1* [81%], *JAK2* [83%], *PAX5* [50%]	*CDKN2A/B* DEL [CN=0]<br>*PAX5* Ex6-10 DEL	*PAX5* alt		*PAX5* alt	90	PREDI-1	NA
2	148	22621	Other	3	F	46,XX,?del(12)(p13)[11]	*ZNF384*-r [57%]; *TCF3*-r [68%]; LOSS: *ETV6* [75%]	*ETV6* DEL	*ZNF384*-r		*ZNF384*-r	88	EOT	NA
2	149	22660	Other	8	F	46,XX,der(12)ins(12;9)(p13;q34q34)[8]	GAIN: *BCR* [73%]	*IKZF1* Ex2-7 DEL<br>*BTG1* Ex2 DEL<br>*CDKN2A/B* DEL [CN=0]	ABL-class			90	PRED1	NA
2	150	22689	Other	7	F	46,XX[20]	*TCF3*-r [77%]; GAIN: *ABL2* [23%]; LOSS: *KMT2A* [20%], *PAX5* [12%]	*CDKN2A* DEL	*TCF3::PBX1*		*TCF3::PBX1*	Unknown	EOT	NA
2	151	22716	Other	2	M	42~47,XY,inc[10]	GAIN: CEP6 [56%], CEP17 [63%], CEP18 [56%], CEPX [55%]	Chr21 GENE GAIN	High Hyperdiploidy		High Hyperdiploidy	95	Maintenance	NA
2	152	22804	Failed	16	M	46,XY[10]			*DUX4*-r		*DUX4*-r	Unknown	Maintenance	NA
2	153	22897	Other	4	F	47,XX,+21,inc[1]	GAIN: *RUNX1* [31%]		*DUX4*-r			95	Maintenance	NA
2	154	22918	Other	12	M	46,XY,add(9)(p?)[4]/46,idem,-7,+mar[3]	LOSS: *PAX5* [81%], *JAK2* [75%]	*CDKN2A/B* DEL<br>*PAX5* DEL	*DUX4*-r		*DUX4*-r	99	Maintenance	NA
2	155	22922	Other	6	M	48,XY,+21c,+21[8]/47,XY,+21c[2]	GAIN: *RUNX1* [84%]	Chr21 GENE GAIN [CN=4]	*BCL2/MYC*			89	Maintenance	NA
2	156	22964	Other	9	M	46,XY,t(7;9)(q11;p13)[6]/45,idem,-8,der(16)t(8;16)(q1;p1)[5]	*PAX5*-r [76%]	*CDKN2A/B* DEL [CN=0]<br>*IKZF1* Ex4-7 DEL	*PAX5* alt		*PAX5* alt	85	PREDI-1	NA
2	157	22972	Other	4	M	46,XY,del(9)(p?21p23)[30]	LOSS: *PAX5* [86%]		*PAX5* alt			87	Maintenance	NA
2	158	22980	Other	1	M	46,XY[20]			Other		*NUTM1*-r	56	Maintenance	NA
2	159	23063	Other	1	F	46,XX,add(2)(q31),add(5)(q35),-9,add(11)(p11.2),add(13)(q14),der(19)t(11;19)(p11.2;p13.1),+mar[cp6]		*CDKN2A/B* DEL	*PAX5* alt			100	Maintenance	NA
2	160	23074	Other	14	M	46,XY[20]		*ETV6* DEL	*DUX4*-r			Unknown	Maintenance	NA
2	161	23075	Other	21	F	46,XX[20]		*PAX5* Ex2-10 DEL<br>*BTG1* Ex2 DEL [CN=0]	*HLF*-r			76	Day 28	27.80%
2	162	23078	Failed	9	M	46,XY[12]	LOSS: *CDKN2A/B* [25%]	*CDKN2A* DEL<br>*ERG* Ex4-7 DEL<br>*IKZF1* Ex4-7 DEL	*DUX4*-r			92	Maintenance	NA
2	163	23081	Other	4	F	47,XX,+X,-9,del(20)(q11q13),+mar[8]/46,XX,add(9)(p13),+add(9)(p13),add(12)(p11),-20,-20[3]	LOSS: *ABL1* (49%), *CDKN2A/B* [46%], *ETV6* [26%]	*CDKN2A* DEL [CN=0]<br>*CDKN2B* DEL<br>*PAX5* Ex1-6 GAIN<br>PAX5 Ex8-10 DEL	*PAX5* alt			95	Maintenance	NA
2	164	23114	Other	7	F	46,XX[20]		*CDKN2B* DEL<br>*IKZF1* Ex2-3 DEL<br>*ERG* Ex4-12 DEL	*DUX4*-r			88	Maintenance	NA

2	165	23132	Other	22	M	46,XY[20]		*CDKN2A/B* DEL	*DUX4*-r	*PAX5* alt	*DUX4*-r	95	Maintenance	NA
2	166	23176	Failed	15	F	Failed	LOSS: *ETV6* [98%], *CDKN2A/B* [96%], *PAX5* [85%], *IKZF1* [89%]	*PAX5* DEL<br>*IKZF1* DEL<br>ETV6 DEL	*PAX5* alt		*PAX5* alt	Unknown	PREDI-1	NA
2	167	23273	Other	2	M	45,XY,der(7)t(7;11)(p22;q11),add(9)(p13),-11[10]	LOSS: *PAX5* [69%]	*CDKN2A/B* DEL	*PAX5* alt			67	Maintenance	NA
2	168	23321	Other	2	M	45,XY,-7,add(11)(q?13),add(12)(p1?1)[5]	LOSS: *ETV6* [89%], *CRLF2* [65%]		*ETV6::RUNX1*-like			97	Maintenance	NA
2	169	23333	Other	10	F	45,XX,+1,add(1)(p1),add(1)(p1),-4,-9,-20,+mar[16]	GAIN: *RUNX1* [20%}, *MEF2D* [83%]; LOSS: *JAK2* [92%], *PAX5* [66%]		*PAX5* alt		*PAX5* alt	96	Maintenance	NA
2	170	23343	Other	1	F	46,XX[20]			*TCF3::PBX1*			95	Maintenance	NA
2	171	23353	Other	1	F	46,XX,add(3)(q24),add(7)(p13),add(12)(p13)[cp6]/47,idem,+X[cp4]		*IKZF1* DEL<br>*ETV6* Ex5-8 DEL	*JAK2*-r		Ph-like	92	Maintenance	NA
2	172	23383	Other	7	M	47,XY,der(21),+?22[16]	GAIN: *RUNX1* [96%]	Chr21 GENE GAIN [CN=3-5]	iAMP21-ALL		iAMP21-ALL	86	Maintenance	NA
2	173	23445	Failed	6	M	46,XY[17]		*IKZF1* Ex4-7 DEL	*DUX4*-r		*DUX4*-r	99	PREDI-1	NA
2	174	23481	Other	18	M	46,XY[20]		*IKZF1* Ex4-8 DEL	*ZNF384*-r			85	Maintenance	NA
2	175	23507	Other	7	F	46,XX,add(2)(p12),add(3)(p22)[4]		*IKZF1* Ex4-7 DEL	*DUX4*-r			90	Maintenance	NA
2	176	23515	Other	2	F	[illegible]-12,add(12)(p1),der(15)t(12;15)(q1;q1),+mar[13]		*IKZF1* DEL<br>*ETV6* DEL	*ETV6::RUNX1*-like			63	Maintenance	NA
2	177	23533	Other	11	M	47,XY,+12[15]	GAIN: *ETV6* [30%]	*CDKN2A/B* DEL	*DUX4*-r			99	PREDI-1	NA
2	178	23560	Other	11	M	47,XXY[20]	GAIN: *RUNX1* [81%], *PDGFRB* [79%]; *PAX5*-r [23%]	*IKZF1* Ex5-8 DEL<br>*BTG1* Ex2<br>*CDKN2A/B* DEL [CN=0]<br>Chr21 GENE GAIN	*PAX5* alt			85	Maintenance	NA
2	179	23650	Other	9	F	50,XX,+X,del(6)(q1?q2?),add(9)(p1?3),+10,+21,+21[5]	GAIN (CN=4): *RUNX1* [82%]; GAIN: *CRLF2* [62%]; LOSS: *PAX5* [68%], *JAK2* [70%], *CDKN2A/B* [73%]	Chr21 GENE GAIN [CN=4]<br>*PAX5* DEL<br>*CDKN2A/B* DEL [CN=0]	*PAX5* alt		*PAX5* alt	90	Maintenance	NA
2	180	23673	Other	2	F	45~46,XX,-1,+mar,inc[cp3]	LOSS (CN=0): *ETV6* [81%]	*ETV6* DEL [CN=0]<br>*IKZF1* DEL	*ETV6::RUNX1*-like		*ETV6::RUNX1*-like	96	PREDI-1	NA
2	181	23678	Other	17	F	46,XX[5]	GAIN: *RUNX1* [59%], *BCR* [59%], *IGH* [62%], *MEF2D* [44%]	Chr21 GENE GAIN	Other	*TCF3*-r, chromosomal gain		90	PREDI-1	NA
2	182	23769	Other	3	M	46,XY[20]			*DUX4*-r			88	PREDI-1	NA
2	183	23813	Other	16	M	Failed	GAIN: *RUNX1* [83%]	Chr21 GENE GAIN<br>PAR1 GAIN<br>*CDKN2A/B* DEL [CN=0]	*ZEB2/CEBP*			90	Maintenance	NA
2	184	23842	Other	18	M	46,XY,del(9)(p2?1)[10]	LOSS: *PAX5* [86%]	*CDKN2A* Ex5 DEL [CN=0]<br>*CDKN2A/B* Ex2 DEL<br>*IKZF1* Ex4-7 DEL<br>PAX5 DEL	*DUX4*-r			99	Week 15	NA
2	185	23863	Other	8	F	46,XX[20]		*IKZF1* Ex4-7 DEL	*DUX4*-r			95	PREDI-1	NA
2	186	23881	Other	3	M	46,XY,?add(2)(q3?5),add(3)(p1?3),del(5)(q31q35),del(9)(p2?3),del(11)(q1?3q2?2),add(19)(p13)[10]		*CDKN2A/B* DEL [CN=0]<br>*PAX5* Ex2-5 GAIN [CN=5]	*PAX5* alt		*PAX5* alt	90	PREDI-1	NA
2	187	23913	Other	5	M	49,XY,+X,del(4)(p15),add(9)(p?13),i(9)(q10),+21,+21[13]	GAIN: *RUNX1* [63%], *ABL1* [38%], *CRLF2* [49%]; LOSS: *JAK2* [44%], *PAX5* [38%]		*PAX5* alt			80	PREDI-1	NA
2	188	23919	Other	3	F	46,XX[20]		*IKZF1* Ex4-7 DEL<br>*PAX5* Ex1-5 DEL<br>*PAR1* DEL	*CRLF2*-r		Other	95	Maintenance	NA
2	189	23922	Other	4	M	47,XY,+X,del(4)(q?),add(11)(p?),del(12)(p1?)[6]/47,XY,+X[2]		PAR1 GAIN<br>*ETV6* DEL	*ETV6::RUNX1*		*ETV6::RUNX1*	90	Maintenance	NA
2	190	23936	Failed	7	F	Failed	LOSS: *JAK2* [75%], *PAX5* [19%]	*CDKN2A/B* Ex2 DEL<br>*IKZF1* Ex4-7 DEL<br>PAX5 DEL	*PAX5* alt		*PAX5* alt	Unknown	PREDI-1	NA
2	191	24041	Other	1	M	46,XY[20]	LOSS: *JAK2* [26%]	*CDKN2A/B* DEL	*PAX5* alt			70	PREDI-1	NA
2	192	24079	Other	13	F	45~46,XX,add(1)(q2?5)[cp5]	LOSS: *ETV6* [54%], *KMT2A* [10%]; GAIN: *ABL1* [10%}	*CDKN2A/B* DEL<br>*ETV6* DEL<br>*RB1* DEL	*MEF2D*-r		*MEF2D*-r	92	Maintenance	NA
2	193	24082	Other	22	F	46,XX[20]		*CDKN2B* DEL	*ZNF384*-r			89	Week 11	NA
2	194	24100	Other	15	M	47,XY,+5,del(9)(p?13p?22)[10]	GAIN: *CSF1R* (78%), *PDGFRB* [83%]	*CDKN2A/B* DEL [CN=0]<br>*EBF1* GAIN	*PAX5* alt		PAX5alt	Unknown	Maintenance	NA
2	195	24232	Other	2	F	46,XX[20]		*ETV6* DEL<br>*PAX5* Ex1-7 DEL<br>*BTG1* Ex2 DEL	*ETV6::RUNX1*-like			97	Maintenance	NA
2	196	24262	Other	5	M	46,XY,del(6)(q21q25)[5]			*PAX5* alt		*PAX5* alt	65	Maintenance	NA
2	197	24332	Other	2	M	46,XY,t(1;15)(q?13;q?22),?del(9)(p?),inc[7]		*CDKN2A/B* DEL [CN=0]	*PAX5* alt		*PAX5* alt	Unknown	PREDI-1	NA
2	198	24338	Other	11	M	47~48,XY,add(3)(p25),-6,del(6)(p22),i(9)(q10),-14,-15,-16,+mar1x2,+mar2,+mar3,+mar4,+mar5[cp8]	GAIN: *BCR* [82%]	*CDKN2A/B* DEL [CN=0]<br>*PAX5* DEL	*IGH::ID4*		*PAX5* alt	95	PREDI-1	NA
2	199	24371	Other	3	M	46,XY[20]	GAIN: *RUNX1* [22%], CEP4 [22%]	Chr21 GENE GAIN	High Hyperdiploidy		High Hyperdiploidy	97	PREDI-1	NA
2	200	24391	Other	14	F	46,XX[20]			*DUX4*-r			99	Maintenance	NA
2	201	24419	Other	17	M	43~46,XY,add(1)(q4),+21[cp9]	GAIN: *RUNX1* [42%], *ABL2* [21%]	Chr21 GENE GAIN	*ZEB2/CEBP*			Unknown	PREDI-1	NA
2	202	24423	Other	12	F	48,XX,+8,+12,add(14)(q32)[18]	GAIN: *ETV6* [93%]	*ETV6* GAIN	*DUX4*-r			94	Day 28	<0.05%
2	203	24456	Other	8	M	46,XY,add(6)(q25-27),add(7)(q?22),add(14)(q32),-17,+mar[6]/46,XY,inv(11)(p11.2-13q23)[5]			*ZNF384*-r		*ZNF384*-r	91	Week 11	NA
2	204	24493	Other	16	M	46,XY,?add(3)(q27),del(13)(q11),t(9;?)(q?;?),del(13)(q?),inc[cp8]	LOSS: *ABL1* [35%]		*MEF2D*-r			65	PRED1	NA
2	205	24566	Failed	14	M	46,XY[10]		*ETV6* DEL<br>*CDKN2A/B* DEL<br>*RB1* Ex14-26 DEL	*ZNF384*-r			98	PRED1	NA
2	206	24601	Other	22	F	46,XX[20]			*ZNF384*-r		*ZNF384*-r	84	PREDI-1	NA
2	207	24607	Other	10	F	46,XX[20]			*ZNF384*-r		*ZNF384*-r	85	Week 11	NA
2	208	24669	Other	1	M	46,XY[20]			Other	*NOTCH2* deletion	Other	93	PREDI-1	NA
2	209	24680	Other	4	M	48,XY,+X,+21c[6]/47,XY,+21c[9]	GAIN: *RUNX1* [100%]	Chr21 GENE GAIN<br>PAR1 GAIN<br>PAR1 DEL	*CRLF2*-r			95	Week 15	NA
2	210	24848	Other	7	M	47,XY,+21c[20]	GAIN: *RUNX1* [100%}	Chr21 GENE GAIN<br>*IKZF1* Ex4-8 DEL	Other	*SH2B3* abnormality		98	Unknown	NA

References
1. Genotype-Specific Minimal Residual Disease Interpretation Improves Stratification in Pediatric Acute Lymphoblastic Leukemia. J Clin Oncol. 2018;36(1):34-43.

Supplementary Table 2: Validation of cytogenetic abnormalities characteristic of established B-ALL subtypes. The patient unique identifier, timepoint germline sample taken and leukemic blast count in germline samples taken on or before end of induction therapy EOI) (weeks 5) are provided. Cytogenetic subgroup and WGS subgroups are provided. Details of individual genetic abnormalities identified by karyotype or FISH are listed. SV or CNA evidence of the variant is categorised as follows: [A] somatic SV/CNA reported by Manta/Canvas and passed filtering procedures. [B] Somatic SV/CNA reported by Manta/Canvas and failed filtering procedures. [C] SV identified through discordantly mapped paired-reads in the bam file. [D] Germline SV/CNV that were reported by Manta/Canvas and passed filtering procedures. [E] No evidence of germline or somatic SV/CNA in the aligned reads. Validation of chromosome ploidy, CNA and/or SV information reported by Manta/Canvas. For the validation of gene fusions, evidence of an SVs reported by Manta was required; CNA information provides additional evidence suggestive of an unbalanced rearrangement. Subgroup-defining genetic abnormalities were not successfully detected in five patients, predominantly as a result of leukemic blast counts (MRD) in the germline sample. DRAGEN T-only analysis was performed on all patients (n=38); subtype defining genetic abnormalities were identified in 37 cases.

Unique ID	Germline sample	Leukemic blast count in EOI samples (%)	Cytogenetic subgroup	WGS subgroup	Other WGS subgroups	Genetic abnormality (method of detection)	Type of genetic abno	SV evidence	CNA evidence	Validated (NSv6)	Comments	Validated (DRAGEN T-only)
28956	Week 5	No result	*BCR::ABL1*	*BCR::ABL1*	High Hyperdiploidy	*BCR::ABL1* (FISH)	Gene fusion	[A] *BCR* Ex1 - *ABL1* Ex2-11<br>[A] *ABL1* Ex1 - *BCR* Ex2-22	[A] GAIN:chr9:130736033-138198851	Yes		Yes
22722	Day 28	>31%	*BCR::ABL1*	*BCR::ABL1*		*BCR::ABL1* (FISH)	Gene fusion	[C] **BCR* Ex1-6 - *ABL1* Ex2-11<br>[C] Downstream of *ABL1* - *BCR* Ex7-22	[A] GAIN:chr9:130764400-130888581	No	Poor mapping quality<br>Complex rearrangement<br>MRD in germline sample	No
10194	Week 11	NA	*BCR::ABL1*	*BCR::ABL1*		*BCR::ABL1* (FISH)	Gene fusion	[A] *BCR* Ex1-13 - *ABL1* Ex2-11<br>[A] *ABL1* Ex1 - *BCR* Ex14-22		Yes		Yes
26681	Week 5	60%	ABL-class	ABL-class		*EBF1::PDGFRB* (FISH/MLPA)	Gene fusion	[C] *EBF1* Ex1-15 - *PDGFRB* Ex11-23	[A,D] LOSS:chr5:150125858-150775683, chr5:158514022-158702617	No	MRD in germline sample	Yes
27442	Week 5	No result	ABL-class	ABL-class		*EBF1::PDGFRB* (FISH/MLPA)	Gene fusion	[C] *EBF1* Ex1-14 - *PDGFRB* Ex11-23	[A] LOSS:chr5:158196464-158703304	No	MRD in germline sample	Yes
28982	Day 30	90%	ABL-class	ABL-class		*EBF1::PDGFRB* (FISH/MLPA)	Gene fusion	[D] *EBF1* Ex1-14 - *PDGFRB* Ex11-23		No	MRD in germline sample	Yes
21190	2 years	NA	*ETV6::RUNX1*	*ETV6::RUNX1*		*ETV6::RUNX1* (FISH)	Gene fusion	[A] *ETV6* Ex1-5 - *RUNX1* Ex2-8		Yes		Yes
20447	2 years	NA	*ETV6::RUNX1*	*ETV6::RUNX1*		*ETV6::RUNX1* (FISH)	Gene fusion	[A] *ETV6* Ex1-5 - *RUNX1* Ex2-8<br>[A] *RUNX1* Ex1 - *ETV6* Ex6-8		Yes		Yes
24553	3 years	NA	*ETV6::RUNX1*	*ETV6::RUNX1*		*ETV6::RUNX1* (FISH)	Gene fusion	[A] *ETV6* Ex1-5 - *RUNX1* Ex2-8		Yes		Yes
20755	Remission	NA	*ETV6::RUNX1*	*ETV6::RUNX1*		*ETV6::RUNX1* (FISH)	Gene fusion	[A] *ETV6* Ex1-5 - *RUNX1* Ex2-8		Yes		Yes
20951	Remission	NA	*ETV6::RUNX1*	*ETV6::RUNX1*		*ETV6::RUNX1* (FISH)	Gene fusion	[A] *ETV6* Ex1-5 - *RUNX1* Ex2-8<br>[A] *RUNX1* Ex1 - *ETV6* Ex6-8		Yes		Yes
27162	Week 5	<0.05%	High Hyperdiploidy	High Hyperdiploidy		53~54,XY,+X[4],+4[3],+6[4],+10[2],+18[3],+21[3],+21[3],inc[cp4] (karyotype)	Chromosome ploidy		[A] GAIN:chr4,6,10,14,17,18,21,21,X	Yes		Yes
27809	Week 5	<0.05%	High Hyperdiploidy	High Hyperdiploidy		56,XX,+X,+4,+5,+6,+10,add(12)(p1?),+14,+17,+18,+21,+21 (karyotype)	Chromosome ploidy		[A] GAIN:chr4,5,6,10,14,17,18,21,21,X<br>[A] CNN-LOH:chr20	Yes		Yes
28593	Day 28	1%	High Hyperdiploidy	High Hyperdiploidy		GAIN (CN=3): CEP6, CEP7, CEP10, IGH, CEP17, CEPX; GAIN (CN=4): RUNX1 (FISH)	Chromosome ploidy		[A] GAIN:chr4,6,7,14,17,18,21,21,X<br>[A] GAIN:chr10:48241-39279665,chr10:42088531-85798273,chr10:116607013-123503437	Yes		Yes
28957	Week 5	<0.05%	High Hyperdiploidy	High Hyperdiploidy		55,XY,+X,+4,+6,add(9)(p2),add(9)(q3),+10,+14,+17,+18,+21,+21 (karyotype)	Chromosome ploidy		[A] GAIN:chr4,6,10,14,17,18,21,21,X	Yes		Yes
28959	Week 5	<0.05%	High Hyperdiploidy	High Hyperdiploidy		55,XX,+X,+6,+10,+14,+17,+18,+21,+21,+mar (karyoptype)	Chromosome ploidy		[A] GAIN:chr6,10,14,17,18,21,21,X,X<br>[A] CNN-LOH:chr4	Yes		Yes
29079	Week 5	<0.05%	High Hyperdiploidy	High Hyperdiploidy		54,XY,+X,+10,+12,+14,+17,+21,+21,+21 (karyotype)	Chromosome ploidy		[A] GAIN:chr10,12,14,17,21,21,21,X<br>[A] CNN-LOH:chr5,16	Yes		Yes
28627	Week 5	<0.05%	High Hyperdiploidy	High Hyperdiploidy		55~56,XY,+X,+6,+8,+10,+14,+15,+17,+18,+21,+21,+mar (karyotype)	Chromosome ploidy		[A] GAIN:chr6,8,10,14,15,17,18,21,21,X	Yes		Yes
10740	Remission	NA	High Hyperdiploidy	High Hyperdiploidy		54-56,XX,+X,+4,+6,+8,+10,+?11,+14,-16,i(17)(q10),+18,+21,+21,+1-2mar (karyotype)	Chromosome ploidy		[A] GAIN:chr6,8,10,11,14,17q,18,21,21,X<br>[A] GAIN:chr4:10001-39425111,chr4:51814822-112919757,chr4:112919758-190112180	Yes		Yes
23110	Week 11	NA	High Hyperdiploidy	High Hyperdiploidy		54,XY,+X,+6,+10,+14,t(14;14)(q11.2;q32),+17,+18,+21,+21 (karyotype)	Chromosome ploidy		[A] GAIN:chr6,10,14,17,18,21,21,X	Yes		Yes
24390	Remission	NA	High Hyperdiploidy	High Hyperdiploidy	*CRLF2*-r	+11,+14,+21,+21c,+X	Chromosome ploidy		[A] GAIN:chr11,14,21,21,21c,X	Yes		Yes
24390	Remission	NA	High Hyperdiploidy	High Hyperdiploidy	*CRLF2*-r	*CRLF2*-r	Gene fusion	[A] *P2RY8* Ex1 - *CRLF2* promoter		Yes		Yes
22340	Maintenanc	NA	iAMP21-ALL	iAMP21-ALL		*RUNX*1 (FISH)	Chromosome ploidy	[A] Chromothripsis:chromosome 21	[A] Chromothripsis:chromosome 21	Yes		Yes
19578	Follow-up	NA	iAMP21-ALL	iAMP21-ALL		*RUNX*1 (FISH)	Chromosome ploidy	[A] Chromothripsis:chromosome 21	[A] Chromothripsis:chromosome 21	Yes		Yes
20724	Day 28	<0.05%	iAMP21-ALL	*DUX4*-r	iAMP21-ALL	*RUNX1* (FISH)	Chromosome ploidy	[A] Chromothripsis:chromosome 21	[A] Chromothripsis:chromosome 21	Yes		Yes
24284	Remission	NA	Low hypodiploidy	Low hypodiploidy		36<1n>,XY,+1,t(1;8)(p1;p1),+5,+6,+8,+9,+10,?add(10)(p1),+11,+14,?add(15)(p1),+16,+18,+19,-+21,+22 (karyotype)	Chromosome ploidy		[A] LOSS:chr2,3,4,7p,12,13,15,16,17,20	Yes		Yes
26772	Week 5	No result	*KMT2A*-r	*KMT2A*-r		*KMT2A*-r (FISH)	Gene fusion	[A] *KMT2A* Ex1-9 - *AFF1* Ex5-21<br>[A] *AFF1* Ex1-4 - *SLCO2B1* - *KMT2A* Ex11-36		Yes		Yes
21318	Week 49	NA	*KMT2A*-r	*KMT2A*-r		*KMT2A*-r (FISH)	Gene fusion	[A] *KMT2A* Ex1-8 - *AFF1* Ex6-21	[A] LOSS:chr11:118482329-118749762	Yes		Yes
21940	Day 28	5.70%	*KMT2A*-r	*KMT2A*-r		*KMT2A*-r (FISH)	Gene fusion	[A] *KMT2A* Ex1-9 - *AFF1* Ex5-21<br>[A] *AFF1* Ex1-4 - *KMT2A* Ex10-36		Yes		Yes
23003	Week 49	NA	*KMT2A*-r	*KMT2A*-r		*KMT2A*-r (karyotype)	Gene fusion	[A] *KMT2A* Ex1-9 - *AFF1* Ex5-21<br>[A] *AFF1* Ex1-4 - *KMT2A* Ex10-36		Yes		Yes

22368	Week 23	NA	*KMT2A* -r	*KMT2A* -r		*KMT2A* -r (FISH)	Gene fusion	[A] *KMT2A* Ex1-7 - *AFF1* Ex8-21<br>[A] *DCTN6* Ex3-7 - *KMT2A* Ex9-36		Yes		Yes
25379	Remission	NA	*KMT2A* -r	*KMT2A* -r		*KMT2A* -r (FISH)	Gene fusion	[A] *KMT2A* Ex1-8 - *AFF1* Ex5-21<br>[A] *AFF1* Ex1-4 - *KMT2A* Ex9-36		Yes		Yes
25647	Week 5	<0.05%	Near haploidy	Near haploidy		53~54,XX,+X,+X,+14,+14,+18,+18,+21,<br>+21(karyotype)	Chromosome ploidy		[A] CNN-LOH:chr1-13,15-17,19,20,22<br>[A] GAIN:chr14,14,18,18,21,21,X,X	Yes		Yes
12202	Remission	NA	Near haploidy	Near haploidy		26<1n>,XY,+18,+21[5]/52,idemx2[3][10]<br>(karyotype)	Chromosome ploidy		[A] CNN-LOH: chr1-17,19,20,22<br>[A] GAIN:chr18,18,21,21,X,Y	Yes		Yes
21708	Remission	NA	Near haploidy	Near haploidy		26<1n>,XY,+14,+21 (karyotype)	Chromosome ploidy		[A] CNN-LOH:chr1-13,15-20,22<br>[A] GAIN:chr14,14,21,21,X	Yes		Yes
7653	Remission	NA	Near haploidy	Near haploidy		28<1n>X,+Y,+8,+10,+18,+21<br>(karyotype)	Chromosome ploidy		[A] CNN-LOH:chr1-7,9,11-17,19,20,22<br>[A] GAIN:chr8,8,10,10,18,18,21,21,X	Yes		Yes
27354	Week 5	<0.05%	*TCF3::PBX1*	*TCF3::PBX1*		t(1;19) (karyotype)	Gene fusion	[A] *TCF3* Ex1-14 - *PBX1* Ex3-9<br>[A] *PBX1* Ex1-2 - *TCF3* Ex2-17	[A] GAIN:chr1:164764564-248526769<br>[A] LOSS:chr19:245713-1616213	Yes		Yes
27397	Week 5	<0.05%	*TCF3::PBX1*	*TCF3::PBX1*		t(1;19) (karyotype/FISH)	Gene fusion	[A] *TCF3* Ex1-14 - *PBX1* Ex3-9	[A] GAIN:chr1:164717488-196744932<br>[A] CNN-LOH:chr19:245407-24330125	Yes		Yes
27304	Unknown	NA	*TCF3::PBX1*	*TCF3::PBX1*		t(1;19) (karyotype/FISH)	Gene fusion	[A] *TCF3* Ex1-14 - *PBX1* Ex3-9	[A] GAIN:chr1:164750170-196747875<br>[A] LOSS:chr19:246622-1617873	Yes		Yes

Supplementary Table 3: Subtype breakdown of individual patients by WGS and WTS. WTS subtypes are defined, as previously described. Patients harbouring genetic abnormalities that characterised two distinct subtypes are highlighted in orange. *Patient 20724 was from cohort 1, this case had genetic abnormalities characteristic of iAMP21-ALL and *DUX4*-r. Nine patients lacked subtype-defining genetic abnormalities (termed 'other' and highlighted in green). However, most other casess (n=8) harboured genetic abnormalities that were clonal or recurrent in this cohort or affected genes that had been previously associated with ALL. Ph-like definition in this table indicates abnormalities that drive the unique GEP of the subtype (Ph-like) that is defined by WTS.

**Unique ID**	**WGS subtype**	**Other WGS subtypes**	**RNA-seq subtype**
20628	ABL-class		Ph-like
9850	ABL-class	*MEF2D*-r	N/A
11959	ABL-class		N/A
22660	ABL-class		N/A
22922	*BCL2/MYC*		N/A
11062	*BCL2/MYC*		N/A
21342	*CRLF2*-r		Ph-like
21869	*CRLF2*-r		Ph-like
11734	*CRLF2*-r		N/A
24680	*CRLF2*-r		N/A
23919	*CRLF2*-r		Other
22418	*JAK2*-r		Ph-like
10310	*DUX4*-r		*DUX4*-r
10876	*DUX4*-r		*DUX4*-r
11672	*DUX4*-r		*DUX4*-r
12134	*DUX4*-r		*DUX4*-r
12356	*DUX4*-r	iAMP21-ALL	*DUX4*-r
12816	*DUX4*-r		*DUX4*-r
20035	*DUX4*-r		*DUX4*-r
20515	*DUX4*-r		*DUX4*-r
20683	*DUX4*-r		*DUX4*-r
20696	*DUX4*-r		*DUX4*-r
20716	*DUX4*-r		*DUX4*-r
20724*	*DUX4*-r	iAMP21-ALL	*DUX4*-r
21568	*DUX4*-r		*DUX4*-r
22006	*DUX4*-r		*DUX4*-r
22037	*DUX4*-r		*DUX4*-r
22045	*DUX4*-r		*DUX4*-r
22387	*DUX4*-r		*DUX4*-r
22804	*DUX4*-r		*DUX4*-r
22918	*DUX4*-r		*DUX4*-r
23132	*DUX4*-r	*PAX5* alt	*DUX4*-r
23445	*DUX4*-r		*DUX4*-r
9469	*DUX4*-r		N/A
10186	*DUX4*-r		N/A
10925	*DUX4*-r		N/A
11053	*DUX4*-r		N/A
11148	*DUX4*-r		N/A
11178	*DUX4*-r		N/A
11556	*DUX4*-r		N/A
11811	*DUX4*-r		N/A
11957	*DUX4*-r		N/A
12083	*DUX4*-r		N/A
12118	*DUX4*-r		N/A
12334	*DUX4*-r		N/A

12460	*DUX4*-r		N/A
12820	*DUX4*-r		N/A
19827	*DUX4*-r		N/A
20720	*DUX4*-r		N/A
21230	*DUX4*-r		N/A
21322	*DUX4*-r		N/A
21437	*DUX4*-r		N/A
21689	*DUX4*-r		N/A
22065	*DUX4*-r		N/A
22224	*DUX4*-r		N/A
22346	*DUX4*-r		N/A
22354	*DUX4*-r		N/A
22355	*DUX4*-r	*ZEB2/CEBP*	N/A
22405	*DUX4*-r		N/A
22417	*DUX4*-r		N/A
22897	*DUX4*-r		N/A
23074	*DUX4*-r		N/A
23078	*DUX4*-r		N/A
23114	*DUX4*-r		N/A
23507	*DUX4*-r		N/A
23533	*DUX4*-r		N/A
23769	*DUX4*-r		N/A
23842	*DUX4*-r		N/A
23863	*DUX4*-r		N/A
24391	*DUX4*-r		N/A
24423	*DUX4*-r		N/A
12335	*DUX4*-r		N/A
23922	*ETV6::RUNX1*		*ETV6::RUNX1*
12729	*ETV6::RUNX1*-like		*ETV6::RUNX1*-like
21198	*ETV6::RUNX1*-like		*ETV6::RUNX1*-like
21321	*ETV6::RUNX1*-like	*CRLF2*-r	*ETV6::RUNX1*-like
22466	*ETV6::RUNX1*-like		*ETV6::RUNX1*-like
23673	*ETV6::RUNX1*-like		*ETV6::RUNX1*-like
21466	*ETV6::RUNX1*-like	Ph-like	Other
10927	*ETV6::RUNX1*-like		N/A
22367	*ETV6::RUNX1*-like		N/A
23321	*ETV6::RUNX1*-like		N/A
23515	*ETV6::RUNX1*-like		N/A
24232	*ETV6::RUNX1*-like		N/A
20764	*ETV6::RUNX1*-like		N/A
12373	High Hyperdiploidy		High Hyperdiploidy
22327	High Hyperdiploidy		High Hyperdiploidy
22716	High Hyperdiploidy		High Hyperdiploidy
24371	High Hyperdiploidy		High Hyperdiploidy
22416	High Hyperdiploidy		N/A
23075	*HLF*-r		N/A
10442	iAMP21-ALL		iAMP21-ALL
21795	iAMP21-ALL		iAMP21-ALL
23383	iAMP21-ALL		iAMP21-ALL
24338	*IGH::ID4*		*PAX*5alt
22572	*IGH::IL3*		Ph-like
9051	*IKZF1* N159Y		*IKZF1* N159Y
21487	*IKZF1* N159Y	*PAX*5alt	N/A
21694	*IKZF1* N159Y		N/A
23353	*JAK2*-r		Ph-like

22104	*JAK2*-r		N/A
11060	Low hypodiploidy		N/A
8451	*MEF2D*-r		*MEF2D*-r
12911	*MEF2D*-r		*MEF2D*-r
24079	*MEF2D*-r		*MEF2D*-r
24493	*MEF2D*-r		N/A
21532	*NUTM1*-r		N/A
22980	Other		*NUTM1*-r
10868	Other	*CNTNAP3B* abnormality, chromosomal gain	Other
22188	Other	*CNTNAP3B* abnormality, *MAPKAP1*-r	Other
24669	Other	*NOTCH2* deletion	Other
24848	Other	*SH2B3* abnormality	N/A
21424	Other	*MAPK10*-r	N/A
23678	Other	*TCF3*-r, chromosomal gain	N/A
11149	Other	*SPOP* abnormality	N/A
12908	*PAX5* P80R		*PAX5* P80R
20874	*PAX5* P80R		*PAX5* P80R
22150	*PAX5* P80R		*PAX5* P80R
22352	*PAX5* P80R		*PAX5* P80R
20756	*PAX5* P80R		N/A
23650	*PAX5* alt		*PAX* 5alt
20706	*PAX* 5alt		*PAX* 5alt
12522	*PAX* 5alt		*PAX* 5alt
21693	*PAX* 5alt		*PAX* 5alt
23913	*PAX* 5alt		N/A
10062	*PAX* 5alt		*PAX* 5alt
11886	*PAX* 5alt		*PAX* 5alt
12910	*PAX* 5alt		*PAX* 5alt
19732	*PAX* 5alt		*PAX* 5alt
22584	*PAX* 5alt		*PAX* 5alt
22964	*PAX* 5alt		*PAX* 5alt
23176	*PAX* 5alt		*PAX* 5alt
23333	*PAX* 5alt		*PAX* 5alt
23881	*PAX* 5alt		*PAX* 5alt
23936	*PAX* 5alt		*PAX* 5alt
24262	*PAX* 5alt		*PAX* 5alt
24332	*PAX* 5alt		*PAX* 5alt
24100	*PAX* 5alt		*PAX* 5alt
10656	*PAX* 5alt		N/A
11832	*PAX* 5alt		N/A
20161	*PAX* 5alt		N/A
21800	*PAX* 5alt		N/A
23063	*PAX* 5alt		N/A
23081	*PAX* 5alt		N/A
23273	*PAX* 5alt		N/A
23560	*PAX* 5alt		N/A
24041	*PAX* 5alt		N/A
22153	*PAX* 5alt		N/A
22972	*PAX* 5alt		N/A
11440	*TCF3::PBX1*		*TCF3::PBX1*
12641	*TCF3::PBX1*		*TCF3::PBX1*
22689	*TCF3::PBX1*		*TCF3::PBX1*
11553	*TCF3::PBX1*		N/A
19730	*TCF3::PBX1*		N/A
20315	*TCF3::PBX1*	*PAX* 5alt	N/A

22245	*TCF3::PBX1*		N/A
23343	*TCF3::PBX1*		N/A
22046	*ZEB2/CEBP*		Other
20317	*ZEB2/CEBP*		*ZEB2/CEBPE*
23813	*ZEB2/CEBP*		N/A
24419	*ZEB2/CEBP*		N/A
8878	*ZNF384*-r		*ZNF384*-r
10184	*ZNF384*-r		*ZNF384*-r
20323	*ZNF384*-r		*ZNF384*-r
22621	*ZNF384*-r		*ZNF384*-r
24456	*ZNF384*-r		*ZNF384*-r
24601	*ZNF384*-r		*ZNF384*-r
24607	*ZNF384*-r		*ZNF384*-r
20653	*ZNF384*-r		N/A
22094	*ZNF384*-r		N/A
23481	*ZNF384*-r		N/A
24082	*ZNF384*-r		N/A
24566	*ZNF384*-r		N/A

References

1. Jeha S, Choi J, Roberts KG, et al. Clinical significance of novel subtypes of acute lymphoblastic leukemia in the context of minimal residual disease-directed therapy. Blood Cancer Discovery. 2021.

Supplementary Table 4: Summary of subtype-defining genetic abnormalities per case. SV details include deletion (DEL), inversion (INV), duplication (DUP) and translocation (BND) with the corresponding breakpoints. CNA are described as loss or gain with the start and end position provided; CN is indicated in square brackets, i.e. [CN=0]. Details of SNV are provided for subtype-defining mutations; VAF - variant allele frequency which is equivalent to abnormal read count divided by total read count for the genomic position. For patients with RNA-seq data (n=85), the key results are provided. Ph-like definition in this table indicates abnormalities that drive the unique GEP of the subtype (Ph-like) that is defined by WTS. Constitutional chromosome 21 gain was detected in all seven Down Syndrome patients (detailed in column D).

Unique ID	WGS subtype 1	Other WGS subtype	Subtype::defining WGS abnormality	Additional details (SV,CNA, SNV)	RNA-seq subgroup	tSNE	PAM	Mutation RNA-seq	Primary fusion RNA-seq	Other fusions - RNA-seq
20628	ABL-class		*ZMYND8::PDGFRB*	BND:chr20:47212716-chr5:150131405	Ph-like	Ph	Ph		*ZMYND8--PDGFRB*	
9850	ABL-class	*MEF2D*-r	*MEF2D::CSF1R*	BND:chr1:156476762-chr5:150066262						
11959	ABL-class		*EBF1::PDGFRB*	DEL:chr5:150128556-158700755						
22660	ABL-class		*ETV6::ABL1*	BND:chr12:11882548-chr9:130706938						
22922	*BCL2/MYC*		Constitutional chr21 gain (+21c)	GAIN:chr21						
22922	*BCL2/MYC*		*BCL6::LPP*	DEL:chr3:187,744,492-188,240,597						
22918	*DUX4*-r		*IGH::DUX4*	*	*DUX4*-r	*DUX4*-r	*DUX4*-r		*IGH--DUX4;DUX4--IGH*	*IGH--LRRK1/LRRK1--IGH*
22918	*DUX4*-r		*ERG* Ex4 INV	INV:chr21:38,439,463-38,493,942	*DUX4*-r	*DUX4*-r	*DUX4*-r		*IGH--DUX4;DUX4--IGH*	*IGH--LRRK1/LRRK1--IGH*
10310	*DUX4*-r		*IGH::DUX4*	*	*DUX4*-r	*DUX4*-r	*DUX4*-r		*IGH--DUX4;DUX4--IGH*	
11672	*DUX4*-r		*IGH::DUX4*	*	*DUX4*-r	*DUX4*-r	*DUX4*-r		*IGH--DUX4;DUX4--IGH*	*PAX5--ZCCH7*
11672	*DUX4*-r		*ERG* Ex4-7 DEL	DEL:chr21:38,401,815-38,504,076	*DUX4*-r	*DUX4*-r	*DUX4*-r		*IGH--DUX4;DUX4--IGH*	*PAX5--ZCCH7*
20696	*DUX4*-r		*IGH::DUX4*	*	*DUX4*-r	*DUX4*-r	*DUX4*-r		*IGH--DUX4;DUX4--IGH*	*PAX5--ZCCH7*
22045	*DUX4*-r		*IGH::DUX4*	*	*DUX4*-r	*DUX4*-r	*DUX4*-r		None Detected	
22804	*DUX4*-r		*IGH::DUX4*	*	*DUX4*-r	*DUX4*-r	*DUX4*-r		*DUX4--IGH;IGH--DUX4*	
22804	*DUX4*-r		*ERG* mutation	frameshift_variant; p.(Ala192GlyfsTer27); VAF=0.17	*DUX4*-r	*DUX4*-r	*DUX4*-r		*DUX4--IGH;IGH--DUX4*	
22804	*DUX4*-r		*ERG* Ex5-11 DEL	DEL:chr21:38,389,967-38,439,565	*DUX4*-r	*DUX4*-r	*DUX4*-r		*DUX4--IGH;IGH--DUX4*	
20035	*DUX4*-r		*IGH::DUX4*	*	*DUX4*-r	*DUX4*-r	*DUX4*-r		None Detected	*CHST2--SLC9A9-AS2;AC018450--SLC9A9-AS2*
20035	*DUX4*-r		*ERG* Ex5-9 DEL	DEL:chr21:38,391,967-38,439,569	*DUX4*-r	*DUX4*-r	*DUX4*-r		None Detected	*CHST2--SLC9A9-AS2;AC018450--SLC9A9-AS2*
20683	*DUX4*-r		*IGH::DUX4*	*	*DUX4*-r	*DUX4*-r	*DUX4*-r		*DUX4--IGH;IGH--DUX4*	
20683	*DUX4*-r		*ERG* Ex1 DEL	DEL:chr21:38,494,811-38,504,063	*DUX4*-r	*DUX4*-r	*DUX4*-r		*DUX4--IGH;IGH--DUX4*	
20683	*DUX4*-r		*ERG* Ex5-9 DEL	DEL:chr21:38,391,967-38,439,565	*DUX4*-r	*DUX4*-r	*DUX4*-r		*DUX4--IGH;IGH--DUX4*	
23132	*DUX4*-r	*PAX5* alt	*IGH::DUX4*	*	*DUX4*-r	*DUX4*-r	*DUX4*-r		*DUX4--IGH;IGH--DUX4*	
23132	*DUX4*-r	*PAX5* alt	*PAX5* Ex4-5 DUP	DUP:chr9:37,002,158-37,007,199	*DUX4*-r	*DUX4*-r	*DUX4*-r		*DUX4--IGH;IGH--DUX4*	
23132	*DUX4*-r	*PAX5* alt	*PAX5* mutation	missense_variant; p.(Val26Gly); sift:deleterious; polyphen:probably damaging; VAF=0.19	*DUX4*-r	*DUX4*-r	*DUX4*-r		*DUX4--IGH;IGH--DUX4*	
20716	*DUX4*-r		*IGH::DUX4*	*	*DUX4*-r	*DUX4*-r	*DUX4*-r		*DUX4--IGH*	
23445	*DUX4*-r		*DUX4L8::MYB*	BND:chr4:190068732-chr6:135194461	*DUX4*-r	*DUX4*-r	*DUX4*-r		None Detected	*PAX5--ZCCHC7*
10876	*DUX4*-r		*IGH::DUX4*	*	*DUX4*-r	*DUX4*-r	*DUX4*-r		None Detected	*MAFF--TPTEP2--CSNK1E*
22006	*DUX4*-r		*IGH::DUX4*	*	*DUX4*-r	*DUX4*-r	*DUX4*-r		None Detected	*ERG-* rearrangement?*
22006	*DUX4*-r		*ERG* mutation	frameshift_variant; p.(Leu372ValfsTer12); VAF=0.08	*DUX4*-r	*DUX4*-r	*DUX4*-r		None Detected	*ERG-* rearrangement?*
22006	*DUX4*-r		*ERG* Ex5-12 DEL	DEL:chr21:38,382,226-38,439,567	*DUX4*-r	*DUX4*-r	*DUX4*-r		None Detected	*ERG-* rearrangement?*
21568	*DUX4*-r		*IGH::DUX4*	*	*DUX4*-r	*DUX4*-r	*DUX4*-r		None Detected	*ERG-*rearrangement?;PAX5--ZCCHC7*
21568	*DUX4*-r		*ERG* Ex5-11 DEL	DEL:chr21:38,389,976-38,439,567	*DUX4*-r	*DUX4*-r	*DUX4*-r		None Detected	*ERG-*rearrangement?;PAX5--ZCCHC7*
12356	*DUX4*-r	iAMP21-ALL	*IGH::DUX4*	*	*DUX4*-r	*DUX4*-r	*DUX4*-r		*IGH--DUX4;DUX4--IGH*	
12356	*DUX4*-r	iAMP21-ALL	*ERG* Ex5-9 DEL	DEL:chr21:38,391,972-38,439,569	*DUX4*-r	*DUX4*-r	*DUX4*-r		*IGH--DUX4;DUX4--IGH*	
12356	*DUX4*-r	iAMP21-ALL	*ERG* Ex5-11 DEL	DEL:chr21:38,390,527-38,439,567	*DUX4*-r	*DUX4*-r	*DUX4*-r		*IGH--DUX4;DUX4--IGH*	
12356	*DUX4*-r	iAMP21-ALL	iAMP21-ALL	Chromothripsis:chr21	*DUX4*-r	*DUX4*-r	*DUX4*-r		*IGH--DUX4;DUX4--IGH*	
22387	*DUX4*-r		*IGH::DUX4*	*	*DUX4*-r	*DUX4*-r	*DUX4*-r		*DUX4--IGH;IGH--DUX4*	
22387	*DUX4*-r		*ERG* Ex5-9 DEL	DEL:chr21:38,391,971-38,439,566	*DUX4*-r	*DUX4*-r	*DUX4*-r		*DUX4--IGH;IGH--DUX4*	
12816	*DUX4*-r		*IGH::DUX4*	*	*DUX4*-r	*DUX4*-r	*DUX4*-r		*DUX4--IGH;IGH--DUX4*	*PAX5--ZCCH7*
12816	*DUX4*-r		*ERG* Ex5-11 DEL	DEL:chr21:38,390,498-38,439,569	*DUX4*-r	*DUX4*-r	*DUX4*-r		*DUX4--IGH;IGH--DUX4*	*PAX5--ZCCH7*
20724	*DUX4*-r	iAMP21-ALL	*IGH::DUX4*	*	*DUX4*-r	*DUX4*-r	*DUX4*-r		*DUX4--IGH;IGH--DUX4*	
20724	*DUX4*-r	iAMP21-ALL	*ERG* Ex4-12 DEL	DEL:chr21:38,377,143-38,498,777	*DUX4*-r	*DUX4*-r	*DUX4*-r		*DUX4--IGH;IGH--DUX4*	
20724	*DUX4*-r	iAMP21-ALL	iAMP21-ALL	Chromothripsis:chr21	*DUX4*-r	*DUX4*-r	*DUX4*-r		*DUX4--IGH;IGH--DUX4*	
12134	*DUX4*-r		*IGH::DUX4*	*	*DUX4*-r	*DUX4*-r	*DUX4*-r		None Detected	*CHST2--SLC9A9-AS2?*
12134	*DUX4*-r		*ERG* Ex5-9 DEL	DEL:chr21:38,391,972-38,439,567	*DUX4*-r	*DUX4*-r	*DUX4*-r		None Detected	*CHST2--SLC9A9-AS2?*
22037	*DUX4*-r		*IGH::DUX4*	*	*DUX4*-r	*DUX4*-r	*DUX4*-r		*IGH--DUX4;DUX4--IGH*	
22037	*DUX4*-r		*ERG* Ex5-11 DEL	DEL:chr21:38,389,561-38,439,569	*DUX4*-r	*DUX4*-r	*DUX4*-r		*IGH--DUX4;DUX4--IGH*	
20515	*DUX4*-r		*IGH::DUX4*	*	*DUX4*-r	*DUX4*-r	*DUX4*-r		*IGH--DUX4;DUX4--IGH*	
20515	*DUX4*-r		*ERG* Ex4 INV	INV:chr21:38,439,465-38,504,075	*DUX4*-r	*DUX4*-r	*DUX4*-r		*IGH--DUX4;DUX4--IGH*	
9469	*DUX4*-r		*IGH::DUX4*	*						
9469	*DUX4*-r		*ERG* Ex5-9 DEL	DEL:chr21:38,391,955-38,439,565						
10186	*DUX4*-r		*IGH::DUX4*	*						
10186	*DUX4*-r		*ERG* mutation	frameshift_variant; p.(Val134ArgfsTer81); VAF=0.12						
10925	*DUX4*-r		*IGH::DUX4*	*						
11053	*DUX4*-r		*IGH::DUX4*	*						
11148	*DUX4*-r		*DUX4::DNTT*	BND:chr4:190092353-chr10:96305952						
11178	*DUX4*-r		*IGH::DUX4*	*						
11556	*DUX4*-r		*IGH::DUX4*	*						
11556	*DUX4*-r		*ERG* Ex5-11 DEL	DEL:chr21:38,390,503-38,439,569						
11811	*DUX4*-r		*IGH::DUX4*	*						
11811	*DUX4*-r		*ERG* Ex5-11 DEL	DEL:chr21:38,390,503-38,439,569						
11957	*DUX4*-r		*IGH::DUX4*	*						
11957	*DUX4*-r		*ERG* Ex5-9 DEL	DEL:chr21:38,391,957-38,439,570						
12083	*DUX4*-r		*IGH::DUX4*	*						
12083	*DUX4*-r		*ERG* Ex5-9 DEL	DEL:chr21:38,391,960-38,439,567						
12118	*DUX4*-r		*IGH::DUX4*	*						
12118	*DUX4*-r		*ERG* Ex4-12 DEL	DEL:chr21:38,367,542-38,504,075						
12334	*DUX4*-r		*IGH::DUX4*	*						
12334	*DUX4*-r		*ERG* Ex5-9 DEL	DEL:chr21:38,391,972-38,439,564						

12460	*DUX4* -r		*IGH::DUX4*	*						
12460	*DUX4* -r		*ERG* Ex4 DEL	DEL:chr21:38,426,202-38,504,076						
12820	*DUX4* -r		*IGH::DUX4*	*						
12820	*DUX4* -r		*ERG* Ex5-9 DEL	DEL:chr21:38,391,967-38,439,567						
19827	*DUX4* -r		*IGH::DUX4*	*						
19827	*DUX4* -r		*ERG* Ex5-11 DEL	DEL:chr21:38,389,542-38,439,583						
20720	*DUX4* -r		*IGH::DUX4*	*						
20720	*DUX4* -r		*ERG* Ex5-9 DEL	DEL:chr21:38,391,965-38,439,573						
21230	*DUX4* -r		*IGH::DUX4*	*						
21230	*DUX4* -r		*ERG* Ex5-12 DEL	DEL:chr21:38,382,224-38,439,567						
21322	*DUX4* -r		*IGH::DUX4*	*						
21322	*DUX4* -r		*ERG* mutation	missense_variant; p.(Phe323Ser); sift:deleterious; polyphen:probably damaging; VAF=0.39						
21437	*DUX4* -r		*IGH::DUX4*	*						
21437	*DUX4* -r		*ERG* Ex1 DEL	DEL:chr21:38,474,417-38,504,072						
21689	*DUX4* -r		*IGH::DUX4*	*						
21689	*DUX4* -r		*ERG::UBE2Z*	BND:chr21:38478083-chr17:48919148						
21689	*DUX4* -r		*UBE2Z::ERG*	BND:chr17:48919168-chr21:38477889						
22065	*DUX4* -r		*IGH::DUX4*	*						
22065	*DUX4* -r		*ERG* mutation	frameshift_variant; p.(Leu297PhefsTer12); VAF=0.45						
22346	*DUX4* -r		*IGH::DUX4*	*						
22346	*DUX4* -r		*ERG* Ex6 DEL	DEL:chr21:38,403,200-38,405,200						
22354	*DUX4* -r		*IGH::DUX4*	*						
22355	*DUX4* -r	*ZEB2/CEBP*	*ERG* mutation	missense_variant; p.(Asp370Ala), VAF=0.46						
22355	*DUX4* -r	*ZEB2/CEBP*	*IGH::SPIDR/CEPBD*	BND:chr14:105864258-chr8:47708775						
22405	*DUX4* -r		*IGH::DUX4*	*						
22405	*DUX4* -r		*ERG* Ex5-9 DEL	DEL:chr21:38,391,890-38,439,573						
22417	*DUX4* -r		*IGH::DUX4*	*						
22417	*DUX4* -r		*ERG* Ex5-9 DEL	DEL:chr21:38,391,947-38,439,569						
22897	*DUX4* -r		*IGH::DUX4*	*						
22897	*DUX4* -r		*ERG* mutation	inframe_insertion; p.(His400delinsLeuTyr); VAF=0.4						
22897	*DUX4* -r		*ERG* Ex5-11 DEL	DEL:chr21:38,389,560-38,439,565						
23074	*DUX4* -r		*IGH::DUX4*	*						
23074	*DUX4* -r		*ERG* Ex6-7 DEL	DEL:chr21:38,402,149-38,405,213						
23078	*DUX4* -r		*IGH::DUX4*	*						
23078	*DUX4* -r		*ERG* Ex4-7 DEL	DEL:chr21:38,401,808-38,493,945						
23114	*DUX4* -r		*IGH::DUX4*	*						
23114	*DUX4* -r		*ERG* Ex4-12 DEL	DEL:chr21:38,367,534-38,504,098						
23507	*DUX4* -r		*IGH::DUX4*	*						
23533	*DUX4* -r		*IGH::DUX4*	*						
23769	*DUX4* -r		*IGH::DUX4*	*						
23769	*DUX4* -r		*ERG* Ex1 DEL	DEL:chr21:38,447,146-38,504,074						
23842	*DUX4* -r		*IGH::DUX4*	*						
23863	*DUX4* -r		*IGH::DUX4*	*						
23863	*DUX4* -r		*ERG* mutation	missense_variant; p.(Lys387Arg); sift:deleterious; polyphen:probably damaging; VAF=0.1						
24391	*DUX4* -r		*IGH::DUX4*	*						
24391	*DUX4* -r		*ERG* Ex4 INV	INV:chr21:38,426,206-38,497,889						
24423	*DUX4* -r		*IGH::DUX4*	*						
22224	*DUX4* -r		*IGH::DUX4*	*						
12335	*DUX4*- r		*IGH::DUX4*	*						
23922	*ETV6::RUNX1*		*ETV6::RUNX1*	BND:chr12:11876166-chr21:34945301	*ETV6::RUNX1*	*ETV6::RUNX1*	*ETV6::RUNX1*		*ETV6--RUNX1*	
12729	*ETV6::RUNX1* -like		*EWSR1::ETV6*	BND:chr22:29298046-chr12:11799904	*ETV6::RUNX1* -like	*ETV6::RUNX1*	*ETV6::RUNX1*		*EWSR1--ETV6;ETV6--FCAMR*	*PAX5--ZCCH7*
23673	*ETV6::RUNX1* -like		*ETV6* LOSS [CN=0]	LOSS:chr12:9056507-12333719	*ETV6::RUNX1* -like	*ETV6::RUNX1*	*ETV6::RUNX1*		None Detected	*PAX5--ASXL1*
23673	*ETV6::RUNX1* -like		*FRS2::IKZF1*	BND:chr12:69574559-chr7:50404662	*ETV6::RUNX1* -like	*ETV6::RUNX1*	*ETV6::RUNX1*		None Detected	*PAX5--ASXL1*
23673	*ETV6::RUNX1* -like		APOBEC mutational signature	MutSig2,13=44.71%	*ETV6::RUNX1* -like	*ETV6::RUNX1*	*ETV6::RUNX1*		None Detected	*PAX5--ASXL1*
21198	*ETV6::RUNX1* -like		*ETV6::USP7*	BND:chr12:11866197-chr16:8961483	*ETV6::RUNX1* -like	*ETV6::RUNX1*	*ETV6::RUNX1*		*CREBBP--LHX4*	
21198	*ETV6::RUNX1* -like		*ETV6* DEL [CN=0]	DEL:chr12:11,710,849-11,977,201	*ETV6::RUNX1* -like	*ETV6::RUNX1*	*ETV6::RUNX1*		*CREBBP--LHX4*	
21198	*ETV6::RUNX1* -like		APOBEC mutational signature	MutSig2,13=56.27%	*ETV6::RUNX1* -like	*ETV6::RUNX1*	*ETV6::RUNX1*		*CREBBP--LHX4*	
22466	*ETV6::RUNX1* -like		*ETV6* DEL	DEL:chr12:11,666,778-11,731,179	*ETV6::RUNX1* -like	*ETV6::RUNX1*	*ETV6::RUNX1*		None Detected	*SHANK3--SBF1;TIAM2--ARID1B;MACF1--MYO18B*
22466	*ETV6::RUNX1* -like		*ETV6* Ex2-4 DEL	DEL:chr12:11,734,075-11,864,384	*ETV6::RUNX1* -like	*ETV6::RUNX1*	*ETV6::RUNX1*		None Detected	*SHANK3--SBF1;TIAM2--ARID1B;MACF1--MYO18B*
22466	*ETV6::RUNX1* -like		*ETV6* LOSS [CN=0]	LOSS:chr12:11666309-11865931	*ETV6::RUNX1* -like	*ETV6::RUNX1*	*ETV6::RUNX1*		None Detected	*SHANK3--SBF1;TIAM2--ARID1B;MACF1--MYO18B*
22466	*ETV6::RUNX1* -like		*IKZF1* LOSS [CN=1]	LOSS:chr7:37234706-50527437	*ETV6::RUNX1* -like	*ETV6::RUNX1*	*ETV6::RUNX1*		None Detected	*SHANK3--SBF1;TIAM2--ARID1B;MACF1--MYO18B*
22466	*ETV6::RUNX1* -like		APOBEC mutational signature	MutSig2,13=46.31%	*ETV6::RUNX1* -like	*ETV6::RUNX1*	*ETV6::RUNX1*		None Detected	*SHANK3--SBF1;TIAM2--ARID1B;MACF1--MYO18B*
21321	*ETV6::RUNX1* -like	*CRLF2* -r	*ETV6::SESN1*	BND:chr12:11802277-chr6:109093867	*ETV6::RUNX1* -like	*ETV6::RUNX1*	*ETV6::RUNX1*		*P2RY8--CRLF2;ETV6--SESN1*	
21321	*ETV6::RUNX1* -like	*CRLF2* -r	*NAP1L1::ETV6*	INV:chr12:11,849,227-76,042,479	*ETV6::RUNX1* -like	*ETV6::RUNX1*	*ETV6::RUNX1*		*P2RY8--CRLF2;ETV6--SESN1*	
21321	*ETV6::RUNX1* -like	*CRLF2* -r	*ETV6* Ex1 DEL	DEL:chr12:11,580,572-11,814,534	*ETV6::RUNX1* -like	*ETV6::RUNX1*	*ETV6::RUNX1*		*P2RY8--CRLF2;ETV6--SESN1*	
21321	*ETV6::RUNX1* -like	*CRLF2* -r	*IKZF1* Ex1-3 INV	INV:chr7:18,351,379-50,342,284	*ETV6::RUNX1* -like	*ETV6::RUNX1*	*ETV6::RUNX1*		*P2RY8--CRLF2;ETV6--SESN1*	
21321	*ETV6::RUNX1* -like	*CRLF2* -r	*IKZF1* Ex1-3 DEL	DEL:chr7:35,699,305-50,342,361	*ETV6::RUNX1* -like	*ETV6::RUNX1*	*ETV6::RUNX1*		*P2RY8--CRLF2;ETV6--SESN1*	
21321	*ETV6::RUNX1* -like	*CRLF2* -r	*P2RY8::CRLF2*	INV:chrX:1,231,926-1,467,109	*ETV6::RUNX1* -like	*ETV6::RUNX1*	*ETV6::RUNX1*		*P2RY8--CRLF2;ETV6--SESN1*	

21321	ETV6::RUNX1 -like	CRLF2 -r	APOBEC mutational signature	MutSig2,13=57.88%	ETV6::RUNX1 -like	ETV6::RUNX1	ETV6::RUNX1		P2RY8--CRLF2;ETV6--SESN1	
21466	ETV6::RUNX1 -like	Ph-like	PHF20::IKZF1	BND:chr20:35852623-chr7:50369225	Other	Ph	High Hyperdiploid		ELF1--FOX01	FOX01--ELF
21466	ETV6::RUNX1 -like	Ph-like	IKZF1 DEL	DEL:chr7:92600325-50369214	Other	Ph	High Hyperdiploid		ELF1--FOX01	FOX01--ELF
21466	ETV6::RUNX1 -like	Ph-like	ETV6 mutation	frameshift_variant; p.(Met43ArgfsTer20), VAF=0.5	Other	Ph	High Hyperdiploid		ELF1--FOX01	FOX01--ELF
21466	ETV6::RUNX1 -like	Ph-like	ELF1::FOX01	DEL:chr13:40,624,420-40,962,971	Other	Ph	High Hyperdiploid		ELF1--FOX01	FOX01--ELF
10927	ETV6::RUNX1 -like		ETV6 LOSS [CN=0]	LOSS:chr12:11680769-11874782						
10927	ETV6::RUNX1 -like		APOBEC mutational signature	MutSig2,13=75.94%						
22367	ETV6::RUNX1 -like		CLIC4::ETV6	BND:chr1:24846798-chr12:11730068						
22367	ETV6::RUNX1 -like		DDX43P2::ETV6	BND:chr7:49033325-chr12:11730283						
22367	ETV6::RUNX1 -like		ZBTB44::ETV6	BND:chr11:130213995-chr12:11769736						
22367	ETV6::RUNX1 -like		MRPL42P4::ETV6	BND:chr7:46932269-chr12:11769738						
22367	ETV6::RUNX1 -like		IKZF1::SCYL3	BND:chr7:50369324-chr1:169855273						
22367	ETV6::RUNX1 -like		IKZF1 Ex4-8 INV	INV:chr7:50,369,127-50,432,127						
22367	ETV6::RUNX1 -like		APOBEC mutational signature	MutSig2,13=64.31%						
23321	ETV6::RUNX1 -like		ETV6::LINC00106	BND:chr12:11758825-chrX:1395565						
23321	ETV6::RUNX1 -like		CD99::ETV6	BND:chrX:2743556-chr12:11775883						
23321	ETV6::RUNX1 -like		IKZF1 LOSS [CN=1]	LOSS:chr7:36441606-55142869						
23321	ETV6::RUNX1 -like		APOBEC mutational signature	MutSig2,13=66.4%						
23515	ETV6::RUNX1 -like		EMILIN2::ETV6	BND:chr12:11765118-chr18:2914846						
23515	ETV6::RUNX1 -like		ETV6::SLC39A13	BND:chr11:47407756-chr12:11765265						
23515	ETV6::RUNX1 -like		IKZF1 LOSS [CN=1]	LOSS:chr7:38362849-53547121						
23515	ETV6::RUNX1 -like		APOBEC mutational signature	MutSig2,13=80.95%						
24232	ETV6::RUNX1 -like		ETV6::TARBP1	BND:chr12:11760224-chr1:234462441						
24232	ETV6::RUNX1 -like		IKZF1 LOSS [CN=1]	LOSS:chr7:45136141-52637623						
24232	ETV6::RUNX1 -like		APOBEC mutational signature	MutSig2,13=7.32%						
20764	ETV6::RUNX1 -like		ETV6 LOSS [CN=0]	DEL:chr12:11,707,929-11,880,177; DEL:chr12:11,694,074-11,870,550						
20764	ETV6::RUNX1 -like		APOBEC mutational signature	MutSig2,13=5.49%						
12373	High Hyperdiploidy		Chromosomal gain	GAIN:chr6,14,21,21,X	High Hyperdiploid	High Hyperdiploid	High Hyperdiploid		None Detected	
24371	High Hyperdiploidy		Chromosomal gain	GAIN:chr4,14,21,21,X	High Hyperdiploid	Ph			ELF1--FOX01	RUNX1 --RCAN1
22327	High Hyperdiploidy		Chromosomal gain	GAIN:chr4,6,14,17,21,21,X	High Hyperdiploid	High Hyperdiploid	High Hyperdiploid		ZNF346--NSD1	
22716	High Hyperdiploidy		Chromosomal gain	GAIN:chr6,9,10,14,17,21,21,X; CNN-LOH:chr22	High Hyperdiploid	High Hyperdiploid	High Hyperdiploid		None Detected	
22416	High Hyperdiploidy		Chromosomal gain	GAIN:chr4,6,7,8,10,12,14,14,17,18,21,21,X						
23075	HLF -r		TCF3::HLF	BND:chr19:1619331-chr17:55315945						
23383	iAMP21-ALL		iAMP21-ALL	Chromothripsis:chr21	iAMP21-ALL	iAMP21-ALL	High Hyperdiploid		ETV6--ITPR2	
10442	iAMP21-ALL		iAMP21-ALL	Abnormal chr21 [CN=5]	iAMP21-ALL	iAMP21-ALL	High Hyperdiploid		None Detected	PAX5--ZCCHC7
21795	iAMP21-ALL		iAMP21-ALL	Chromothripsis:chr21	iAMP21-ALL	High Hyperdiploid	High Hyperdiploid		None Detected**	
22340	iAMP21-ALL		iAMP21-ALL	Chromothripsis:chr21	iAMP21-ALL	iAMP21	High Hyperdiploid		None Detected	
9051	IKZF1 N159Y		IKZF1 Ex5 DUP	DUP:chr7:50,382,361-50,382,856	IKZF1 N159Y	IKZF1 N159Y	BCL2/MYC	IKZF1 N159Y	INP5D--DGKD	
9051	IKZF1 N159Y		IKZF1 mutation	missense_variant; p.(Asn159Tyr); VAF=0.32	IKZF1 N159Y	IKZF1 N159Y	BCL2/MYC	IKZF1 N159Y	INP5D--DGKD	
21487	IKZF1 N159Y	PAX5 alt	IKZF1 Ex5 DUP	DUP:chr7:50,382,257-50,382,810						
21487	IKZF1 N159Y	PAX5 alt	IKZF1 mutation	missense_variant; p.(Asn159Tyr); VAF=0.55						
21487	IKZF1 N159Y	PAX5 alt	PAX5 mutation	missense_variant; p.(Arg38His); VAF=0.11						
21694	IKZF1 N159Y		IKZF1 Ex5 DUP	DUP:chr7:50,381,660-50,386,282						
21694	IKZF1 N159Y		IKZF1 mutation	missense_variant; p.(Asn159Tyr); VAF=0.29						
11060	Low hypodiploidy		Chromosomal ploidy	GAIN:chr1,2,4,5,6,6,8,8,10,11,11,12,13,14,16,17,18,21,21,22,22; CNN-LOH:chr3,7,9,15						
8451	MEF2D -r		MEF2D::BCL9	INV:chr1:147,621,484-156,479,565	MEF2D -r	MEF2D -r	MEF2D -r		MEF2D--BCL9	
12911	MEF2D -r		MEF2D::BCL9	INV:chr1:147,620,960-156,477,651	MEF2D -r	MEF2D -r	MEF2D -r		MEF2D--BCL9	IGLL1--MYO18B;PAX5--ZCCH7
24079	MEF2D -r		MEF2D::SS18	BND:chr1:156,465,667-chr18:26,058,652	MEF2D -r	MEF2D -r	MEF2D -r		MEF2D--SS18	*RBFOX2?;PAX5--ZCCH7
24493	MEF2D -r		MEF2D::BCL9	INV:chr1:147,622,992-156,478,009						
21532	NUTM1 -r		KAT6A::NUTM1	BND:chr8:41,960,975-chr15:34,354,440						
10868	Other	CNTNAP3B abnormality, chromosomal gain	CNTNAP3B-C20orf203	BND:chr9:41967673-chr20:32665686	Other	iAMP21-ALL	High Hyperdiploid		None Detected	
10868	Other	CNTNAP3B abnormality, chromosomal gain	Chromosomal gain	GAIN:chr10,21,X	Other	iAMP21-ALL	High Hyperdiploid		None Detected	
23650	PAX5 alt		PAX5 LOSS [CN=1]	LOSS:chr9:112,753-40,846,154	PAX5 alt	Ph	PAX5 alt		None Detected	HIST1H2BD--TSC22D3
23650	PAX5 alt		CDKN2A/B DEL [CN=0]	DEL:chr9:21,969,000-22,008,352	PAX5 alt	Ph	PAX5 alt		None Detected	HIST1H2BD--TSC22D3
22980	Other				NUTM1				ZNF618--NUTM1	PAX5--ZCCH7
22188	Other	CNTNAP3B abnormality, MAPKAP1 -r	CNTNAP3B mutation	missense_variant; p.(Gly520Ala); VAF=0.24	Other				None Detected	ADAM17--BCL11A
22188	Other	CNTNAP3B abnormality, MAPKAP1 -r	RRAGB::MAPKAP1	BND:chrX: 55748971-chr9:125458893	Other				None Detected	ADAM17--BCL11A
24669	Other	NOTCH2 deletion	NOTCH2 deletion [CN=1]	LOSS:chr1:119983209-121429772	Other				None Detected	
24848	Other	SH2B3 abnormality	Constitutional chr21 gain (+21c)	GAIN:chr21						
24848	Other	SH2B3 abnormality	SH2B3 mutation	inframe_insertion; p.(Val289_Gly290insPro); VAF=0.46						
21424	Other	MAPK10 -r	UBA6_AS1::MAPK10	INV:chr4:67888668-86574638						
11062	BCL2/MYC		UBA6_AS1::MAPK10	INV:chr4:67888668-86574638						
11062	BCL2/MYC		IGH::CASC11/MYC	BND:chr14:105745489-chr8:127539083						
11149	Other	SPOP abnormality	SPOP mutation	inframe_insertion; p.(Asp130delinsGluGlyLysIleLeuSerAspAla), VAF=0.49; missense_variant; p.(Asp130Asn); , VAF=0.43						
23678	Other	TCF3 -r, chromosomal gain	TCF3::MEF2B	DUP:chr19:1,617,188-19,161,095						
23678	Other	TCF3 -r, chromosomal gain	TCF3::ZNF30	DUP:chr19:1,651,554-34,936,180						
23678	Other	TCF3 -r, chromosomal gain	CEBPA::TCF3	DEL:chr19:1,650,826-33,274,300						
23678	Other	TCF3 -r, chromosomal gain	Chromosomal gain	GAIN:chr8,21,22						
22150	PAX5 P80R		PAX5 mutation	missense_variant; p.(Pro80Arg), VAF=0.97	PAX5 P80R	PAX5 P80R	PAX5 P80R	PAX5 P80R	None Detected	
12908	PAX5 P80R		PAX5 mutation	missense_variant; p.(Pro80Arg), VAF=0.65	PAX5 P80R	PAX5 P80R	PAX5 P80R	PAX5 P80R	None Detected	TPM4--KLF2
22352	PAX5 P80R		PAX5 mutation	missense_variant; p.(Pro80Arg), VAF=0.47	PAX5 P80R	PAX5 P80R	PAX5 P80R	PAX5 P80R	None Detected	PAX5--ZCCH7
20874	PAX5 P80R		PAX5 mutation	missense_variant; p.(Pro80Arg), VAF=0.92	PAX5 P80R	PAX5 P80R	PAX5 P80R	PAX5 P80R	None Detected	IGH--CRADD

20756	PAX5 P80R		PAX5 mutation	missense_variant; p.(Pro80Arg); VAF=0.92						
24262	PAX5 alt		PAX5 mutation	frameshift_variant; p.(Lys94ArgfsTer66), VAF=0.33<br>(29/87 reads)	PAX5 alt	PAX5 alt	Low hypodiploid		None Detected	
20706	PAX5 alt		PAX5 LOSS [CN=1]	LOSS:chr9:27812750-38773965	PAX5 alt	PAX5 alt	PAX5 alt		None Detected	
20706	PAX5 alt		CDKN2A/B LOSS [CN=0]	LOSS:chr9:21952924-22218768	PAX5 alt	PAX5 alt	PAX5 alt		None Detected	
20706	PAX5 alt		MTAP INV	INV:chr9:21,122,905-21,827,697	PAX5 alt	PAX5 alt	PAX5 alt		None Detected	
10062	PAX5 alt		PAX5 intragenic DEL [CN=0]	DEL:chr9:37,025,241-37,030,754	PAX5 alt	PAX5 alt	PAX5 alt		P2RY8--CRLF2	
12522	PAX5 alt		MTAP::MAPK10	BND:chr9:21837463-chr4:86335647	PAX5 alt	PAX5 alt	PAX5 alt		None Detected	MTAP--MAPK10;AL359922.1--MAPK10
12522	PAX5 alt		CDKN2A/B LOSS [CN=0]	LOSS:chr9:21840014-28626546	PAX5 alt	PAX5 alt	PAX5 alt		None Detected	MTAP--MAPK10;AL359922.1--MAPK10
12522	PAX5 alt		PAX5 LOSS [CN=1]	LOSS:chr9:28983585-37421440	PAX5 alt	PAX5 alt	PAX5 alt		None Detected	MTAP--MAPK10;AL359922.1--MAPK10
12910	PAX5 alt		PAX5::FBRSL1	BND:chr9:36951367-chr12:132565042	PAX5 alt	PAX5 alt	PAX5 alt		PAX5--FBRSL1	FBRSL1--RBP5
19732	PAX5 alt		PAX5 Ex9 DEL [CN=0]	DEL:chr9:36841033-36853879	PAX5 alt	PAX5 alt	PAX5 alt		None Detected	
23176	PAX5 alt		PAX5 mutation	splice_acceptor_variant; c.1013-1G>A, VAF=0.66	PAX5 alt	PAX5 alt	PAX5 alt		None Detected	PAX5--ZCCHC7
23333	PAX5 alt		PAX5::NOL4L	BND:chr9:36994004-chr20:32452327	PAX5 alt	PAX5 alt	PAX5 alt		PAX5--NOL4L	WARS2--COL16A1
23936	PAX5 alt		PAX5 mutation	splice_donor_variant; c.1099+1G>C, VAF=0.72	PAX5 alt	PAX5 alt	PAX5 alt		BCR--ABL	
23881	PAX5 alt		PAX5 Ex2-5 DUP	INV:chr9:36,995,339-37,026,089	PAX5 alt	PAX5 alt	PAX5 alt		None Detected	*--PTMA;ELF1--LINC00571
24100	PAX5 alt		PAX5 Ex5 INDEL	g.37002672_37002677delinsTTGGTGAAGGGAGGGAGGGAAGGGAA<br>GGAAGGGAAGGAAGGGAGGAAGGAAGGGAGGGAAGGAAGGAAG<br>GAAGGAAGGAAGGAAGGAAGGAAGGAAGGGAAGGAAGGGAGGG<br>AAGGAAGGGAAGGAAGGGAGGGAGGGAAGGGAAGGAAGGGAGG<br>GAAGGAAGGGAAGGAAGGGAGGGAAGGAAGGAAGGAAGGAAGG<br>GAGGGAAGGGAAGGAAGGAAGGGAAGGAAGGGAGGAAGGAAGG<br>GAGGGAAGGGAGGGAATCCT; VAF=0.62	PAX5 alt	PAX5 alt	PAX5 alt		None Detected	
21693	PAX5 alt		PAX5 LOSS [CN=1]	LOSS:chr9:22151782-38773616	PAX5 alt	PAX5 alt	PAX5 alt		None Detected	RBM34--TARBP1
21693	PAX5 alt		CDKN2A/B LOSS [CN=0]	LOSS:chr9:20,102,121-22,151,781	PAX5 alt	PAX5 alt	PAX5 alt		None Detected	RBM34--TARBP1
21693	PAX5 alt		MTAP LOSS [CN=0]	LOSS:chr9:20,102,121-22,151,781	PAX5 alt	PAX5 alt	PAX5 alt		None Detected	RBM34--TARBP1
24332	PAX5 alt		PAX5::STOML1	BND:chr9:36934456-chr15:73964987	PAX5 alt	PAX5 alt	PAX5 alt		PML--PAX5	PAX5--PML;PAX5--ZCCH7
22584	PAX5 alt		PAX5::ZNF521	BND:chr9:36986465-chr18:25332478	PAX5 alt	PAX5 alt	PAX5 alt		ZNF521--PAX5;PAX5--AUTS2	PAX5--ZNF521
11886	PAX5 alt		PAX5::ELN	BND:chr9:36905811-chr7:74029556	PAX5 alt	PAX5 alt	PAX5 alt		PAX5--ELN	PAX5--ZCCH7
22964	PAX5 alt		PAX5::AUTS2	BND:chr9:36961411-chr7:70487638	PAX5 alt	PAX5 alt	PAX5 alt		PAX5--AUTS2	AUTS2--PAX5
24338	IGH::ID4		IGH::ID4	BND:chr14:105863861-chr6:19978527	PAX5 alt	PAX5 alt	PAX5 alt		None Detected	
23913	PAX5 alt		PAX5 LOSS [CN=1]	LOSS:chr9:22198831-39011843						
23913	PAX5 alt		CDKN2A/B LOSS [CN=0]	LOSS:chr9:21309260-22198830						
23913	PAX5 alt		MTAP LOSS [CN=0]	LOSS:chr9:21309260-22198830						
10656	PAX5 alt		PAX5 Ex8 mutation	splice_region_variant; c.911-6C>T; VAF=0.81						
11832	PAX5 alt		PAX5::FBRS	BND:chr9:36961882-chr16:30665970						
20161	PAX5 alt		PAX5 mutation	missense_variant; p.(Gly76Glu); VAF=0.43						
20161	PAX5 alt		PAX5 mutation	missense_variant; p.(Arg38His); VAF=0.33						
21800	PAX5 alt		PAX5::AUTS2	BND:chr9:36927948-chr7:70352475						
22972	PAX5 alt		PAX5 LOSS [CN=1]	LOSS:chr9:25933079-36878357						
22972	PAX5 alt		PAX5 Ex6-8 LOSS [CN=0]	LOSS:chr9:36878358-36990568						
22972	PAX5 alt		CDKN2A/B LOSS [CN=1]	LOSS:chr9:20366936-25928943						
22972	PAX5 alt		MTAP LOSS [CN=1]	LOSS:chr9:20366936-25928943						
23063	PAX5 alt		PAX5::DACH1	BND:chr9:36906250-chr13:71585902						
23081	PAX5 alt		PAX5::NOL4L	BND:chr9:36961956 -chr20:32456159						
23273	PAX5 alt		PAX5::CPSF7	BND:chr9:36947562-chr11:61428220						
23560	PAX5 alt		PAX5::EBF1::AUTS2	BND:chr9:36950384-chr5:158887706-chr5:158876971-chr7:70184619						
23560	PAX5 alt		IGL::PAX5	BND:chr22:22202132-chr9:36950650						
24041	PAX5 alt		PAX5::AUTS2	BND:chr9:36951866-chr7:70495108						
22153	PAX5 alt		PAX5 Ex5 INSERTION	g.37002675insCGAGGAAGGAAGGGAGGGAGGGAAGGAAGGGAGG<br>GAAGGAAGGAAGGGAGGGAAGGAAGGAAGGGAAGGAAGGAAGG<br>GAGGGAAGGAAGGGAGGGAGGGAAGGAAGGGAGGGAGGGAAGG<br>AAGGGAGGGAAGGAAGTAAGGAAGGGAAGGAAGGAAGGGAAGG<br>GAGGGAAGGAAGGAAGGGAAGGGAGGGAGGTAAGGAAGGAAGG<br>AAGGGAAGGAAGGAAGGGAAGGGAGGGAGGGAAGGAAGGGAGG<br>A; VAF=0.82						
21869	CRLF2-r		Constitutional chr21 gain (+21c)	GAIN:chr21	Ph-like	Ph	Ph		P2RY8--CRLF2	PAX5--ZCCH7
21869	CRLF2-r		P2RY8::CRLF2	DEL:chrX:1,216,130-1,536,516	Ph-like	Ph	Ph		P2RY8--CRLF2	PAX5--ZCCH7
21342	CRLF2-r		Constitutional chr21 gain (+21c)	GAIN:chr21	Ph-like	Ph			P2RY8--CRLF2	
21342	CRLF2-r		P2RY8::CRLF2	DEL:chrX:1,216,120-1,536,523	Ph-like	Ph			P2RY8--CRLF2	
22572	IGH::IL3		IGH::IL3	BND:chr14:105863255-chr5:132059721	Ph-like	Ph			None Detected	ZCCHC7--PAX5
22418	JAK2-r		PAX5::JAK2	INV:chr9:5081416-37004391	Ph-like	Ph	Ph		PAX5--JAK2	JAK2--PAX5
23353	JAK2-r		ETV6::JAK2	BND:chr12:11860782-chr9:5078604	Ph-like	Ph	Ph		ETV6--JAK2	
23919	CRLF2-r		P2RY8::CRLF2	DEL:chrX:1,216,129-1,536,517	Other	High Hyperdiploid	High Hyperdiploid		P2RY8--CRLF2	
11734	CRLF2-r		Constitutional chr21 gain (+21c)	GAIN:chr21						
11734	CRLF2-r		P2RY8::CRLF2	DEL:chrX:1,216,132-1,536,516						
24680	CRLF2-r		Constitutional chr21 gain (+21c)	GAIN:chr21						
24680	CRLF2-r		P2RY8::CRLF2	DEL:chrX:1216184-1536598						
22104	JAK2-r		PAX5::JAK2	INV:chr9:5081218-36995317						
11440	TCF3::PBX1		TCF3::PBX1	BND:chr19:1617928-chr1:164784673	TCF3::PBX1	TCF3::PBX1	TCF3::PBX1		TCF3--PBX1	PAX5--ZCCH7
12641	TCF3::PBX1		TCF3::PBX1	BND:chr19:1617927-chr1:164786364	TCF3::PBX1	TCF3::PBX1	TCF3::PBX1		TCF3--PBX1	
22689	TCF3::PBX1		TCF3::PBX1	BND:chr19:1617933-chr1:164687106	TCF3::PBX1	TCF3::PBX1	TCF3::PBX1		TCF3--PBX1	
11553	TCF3::PBX1		TCF3::PBX1	BND:chr19:1617933-chr1:164701489						
19730	TCF3::PBX1		TCF3::PBX1	BND:chr19:1617933-chr1:164699328						
20315	TCF3::PBX1	PAX5 alt	TCF3::PBX1	BND:chr19:1617934-chr1:164689619						
20315	TCF3::PBX1	PAX5 alt	PAX5 mutation	frameshift_variant; p.(Leu119AlafsTer8), vaf=0.25						

22245	*TCF3::PBX1*		*TCF3::PBX1*	BND:chr19:1617929-chr1:164713346						
23343	*TCF3::PBX1*		*TCF3::PBX1*	BND:chr19:1617929-chr1:164720634						
22046	*ZEB2/CEBP*		Constitutional chr21 gain (+21c)	GAIN:chr21	Other	High Hyperdiploid	High Hyperdiploid		None Detected	
22046	*ZEB2/CEBP*		*HMGB1::CEBPB*	BND:chr13:30,460,107-chr20:50,237,566	Other	High Hyperdiploid	High Hyperdiploid		None Detected	
22046	*ZEB2/CEBP*		*ZEB2* mutation	missense_variant; p.(His1063Arg); VAF=0.06	Other	High Hyperdiploid	High Hyperdiploid		None Detected	
20317	*ZEB2/CEBP*		*IGH::CEBPE*	INV:chr14:23119054-106592676	*ZEB2/CEBPE*	*ZEB2/CEBPE*		*ZEB2* H1038R	None Detected	*PAX5--ZCCH7*
20317	*ZEB2/CEBP*		*ZEB2* mutation	missense_variant; p.(His1063Arg); VAF=0.48	*ZEB2/CEBPE*	*ZEB2/CEBPE*		*ZEB2* H1038R	None Detected	*PAX5--ZCCH7*
23813	*ZEB2/CEBP*		*IGH::CEBPA*	BND:chr14:105894514-chr19:33300778						
24419	*ZEB2/CEBP*		*ZEB2* mutation	missense_variant; p.(His1063Arg); VAF=0.34						
24419	*ZEB2/CEBP*		*CEBPB::LINC01058*	BND:chr13:30420256-chr20:50235637						
8878	*ZNF384*-r		*CREBBP::ZNF384*	BND:chr16:3774769-chr12:6688782	*ZNF384*-r	*ZNF384*-r	*ZNF384*-r		*CREBBP--ZNF384*	
10184	*ZNF384*-r		*EP300::ZNF384*	BND:chr22:41135294-chr12:6682158	*ZNF384*-r	*ZNF384*-r	*ZNF384*-r		*EP300--ZNF384*	
20323	*ZNF384*-r		*SMARCA2::ZNF384*	BND:chr9:2040119-chr12:6687288	*ZNF384*-r	*ZNF384*-r	*ZNF384*-r		*SMARCA2-ZNF384*	
22621	*ZNF384*-r		*TCF3::ZNF384*	BND:chr19:1613518-chr12:6677720	*ZNF384*-r	*ZNF384*-r	*ZNF384*-r		*TCF3--ZNF384*	
24601	*ZNF384*-r		*EP300::ZNF384*	BND:chr22:41133427-chr12:6686311	*ZNF384*-r	*ZNF384*-r	*ZNF384*-r		*EP300--ZNF384*	*ZNF384--EP300*
24456	*ZNF384*-r		*EP300::ZNF384*	BND:chr22:41132302-chr12:6684892	*ZNF384*-r	*ZNF384*-r	*ZNF384*-r		*EP300--ZNF384*	*ZNF384--EP300*
24607	*ZNF384*-r		*EP300::ZNF384*	BND:chr22:41134567-chr12:6688073	*ZNF384*-r	*ZNF384*-r	*ZNF384*-r		*EP300--ZNF384*	*ZNF384--EP300*
20653	*ZNF384*-r		*EP300::ZNF384*	BND:chr22:41133284-chr12:6681125						
22094	*ZNF384*-r		*EP300::ZNF384*	BND:chr22:41134983-chr12:6684161						
23481	*ZNF384*-r		*EP300::ZNF384*	BND:chr22:41134172-chr12:6680434						
24082	*ZNF384*-r		*EP300::ZNF384*	BND:chr22:41135337-chr12:6680643						
24566	*ZNF384*-r		*TCF3::ZNF384*	BND:chr19:1620565-chr12:6682757						

Supplementary Table 5: Type of genetic abnormalities that characterised *PAX5* alt and *ETV6::RUNX1* -like cases. *Expanded subtype-defining genetic abnormalities identified in this study included: a) large insertion (≤250bp) within exon 5 of *PAX5* and b) *PAX5* loss [CN=1] with *CDKN2A* and *CDKN2B* loss [CN=0], often with biallelic *MTAP* abnormalities, that lack other defining features. **One patient (21198) harboured a fusion gene involving *ETV6* and biallelic loss of *ETV6* .

	PAX5 alt		*ETV6::RUNX1* -like	
	PAX5 abnormality (occurrence in cohort)	Patient ID	*ETV6* abnormality (occurrence in cohort)	Patient ID
Fusion gene	13	12910, 23333, 24332, 22584, 22964, 11886, 11832, 21800, 23081, 23063, 23273, 23560, 24041	7**	12729, 21198, 21321, 22367, 23321, 23515, 24232
Biallelic loss	3	10062, 19732, 22972	5**	23673, 21198, 22466, 10927, 20764
Internal Tandem Duplication (ITD)	1	23881		
Mutation	5	24262, 23176, 23936, 10656, 20161	1	21466
Insertion*	2	24100, 22153		
Monoallelic loss*	5	20706, 12522, 21693, 23913, 23650		
Total	**29**		**12**	

Supplementary Table 6: *IGH::DUX4* detection. Diagnostic (n=213) and matched germline (n=211) samples of 213 patients were screened for reads that spanned the *IGH* and *DUX4* loci. Spanning reads per billion (SRPB) are shown. Samples with >10 SRPB were defined as *IGH::DUX4*. Two patients harboured *DUX4* rearrangements that involved other fusion gene partners (*MYB* (23445) and *DNTT* (11148)); these samples had SRPB values of 0. The highest SRPB value in diagnostic samples that represented other (non-*IGH::DUX4*) ALL subtypes and the matched germline samples was 4.6 and 9.6, respectively. Ph-like definition in this table indicates abnormalities that drive the unique GEP of the subtype (Ph-like) that is defined by WTS.

Unique ID	Disease stage	ALL subtype	Other ALL subtypes	Spanning reads per billion (SRPB)	*IGH::DUX4* status (for Figure 5a)
12460	Diagnosis	*DUX4*-r		157.3082367	*IGH::DUX4*
23132	Diagnosis	*DUX4*-r	*PAX5* alt	143.7011191	*IGH::DUX4*
23507	Diagnosis	*DUX4*-r		139.5738719	*IGH::DUX4*
22804	Diagnosis	*DUX4*-r		131.3747682	*IGH::DUX4*
23863	Diagnosis	*DUX4*-r		122.3523983	*IGH::DUX4*
21322	Diagnosis	*DUX4*-r		115.7722424	*IGH::DUX4*
20683	Diagnosis	*DUX4*-r		113.4308455	*IGH::DUX4*
22065	Diagnosis	*DUX4*-r		113.1879062	*IGH::DUX4*
11178	Diagnosis	*DUX4*-r		109.1383894	*IGH::DUX4*
22897	Diagnosis	*DUX4*-r		107.2037481	*IGH::DUX4*
23114	Diagnosis	*DUX4*-r		102.0786235	*IGH::DUX4*
23769	Diagnosis	*DUX4*-r		98.80622289	*IGH::DUX4*
20720	Diagnosis	*DUX4*-r		97.22633894	*IGH::DUX4*
21230	Diagnosis	*DUX4*-r		96.51749613	*IGH::DUX4*
20696	Diagnosis	*DUX4*-r		95.24807145	*IGH::DUX4*
12816	Diagnosis	*DUX4*-r		92.74703227	*IGH::DUX4*
11556	Diagnosis	*DUX4*-r		91.18711473	*IGH::DUX4*
20515	Diagnosis	*DUX4*-r		89.57184104	*IGH::DUX4*
11672	Diagnosis	*DUX4*-r		89.43864724	*IGH::DUX4*
12083	Diagnosis	*DUX4*-r		88.7773509	*IGH::DUX4*
24391	Diagnosis	*DUX4*-r		88.18365899	*IGH::DUX4*
23842	Diagnosis	*DUX4*-r		86.70897938	*IGH::DUX4*
23533	Diagnosis	*DUX4*-r		86.24334745	*IGH::DUX4*
11811	Diagnosis	*DUX4*-r		83.79125844	*IGH::DUX4*
10186	Diagnosis	*DUX4*-r		79.72168457	*IGH::DUX4*
22045	Diagnosis	*DUX4*-r		78.84478994	*IGH::DUX4*
12118	Diagnosis	*DUX4*-r		78.80381941	*IGH::DUX4*
24423	Diagnosis	*DUX4*-r		74.10904474	*IGH::DUX4*
22354	Diagnosis	*DUX4*-r		73.33127486	*IGH::DUX4*
23078	Diagnosis	*DUX4*-r		72.73590261	*IGH::DUX4*
10925	Diagnosis	*DUX4*-r		72.59659059	*IGH::DUX4*
9469	Diagnosis	*DUX4*-r		72.45510816	*IGH::DUX4*
22405	Diagnosis	*DUX4*-r		67.83820355	*IGH::DUX4*
11053	Diagnosis	*DUX4*-r		64.92917018	*IGH::DUX4*
20716	Diagnosis	*DUX4*-r		58.71424704	*IGH::DUX4*
21437	Diagnosis	*DUX4*-r		57.8718901	*IGH::DUX4*
22346	Diagnosis	*DUX4*-r		55.46807985	*IGH::DUX4*
12134	Diagnosis	*DUX4*-r		54.11592437	*IGH::DUX4*
22918	Diagnosis	*DUX4*-r		51.75976253	*IGH::DUX4*
22006	Diagnosis	*DUX4*-r		51.58389428	*IGH::DUX4*
20724	Diagnosis	*DUX4*-r	iAMP21-ALL	51.20961296	*IGH::DUX4*
21689	Diagnosis	*DUX4*-r		47.69411227	*IGH::DUX4*
19827	Diagnosis	*DUX4*-r		45.84606575	*IGH::DUX4*
12820	Diagnosis	*DUX4*-r		44.63497616	*IGH::DUX4*
12356	Diagnosis	*DUX4*-r	iAMP21-ALL	43.78234601	*IGH::DUX4*
22387	Diagnosis	*DUX4*-r		39.92367375	*IGH::DUX4*
20035	Diagnosis	*DUX4*-r		38.80968692	*IGH::DUX4*
10876	Diagnosis	*DUX4*-r		38.39523879	*IGH::DUX4*
22037	Diagnosis	*DUX4*-r		37.01774906	*IGH::DUX4*
21568	Diagnosis	*DUX4*-r		35.8466294	*IGH::DUX4*
23074	Diagnosis	*DUX4*-r		34.12032073	*IGH::DUX4*
22417	Diagnosis	*DUX4*-r		34.02693243	*IGH::DUX4*
11957	Diagnosis	*DUX4*-r		33.96189142	*IGH::DUX4*
12334	Diagnosis	*DUX4*-r		32.74079291	*IGH::DUX4*
22224	Diagnosis	*DUX4*-r		17.32242875	*IGH::DUX4*
12335	Diagnosis	*DUX4*-r		13.96115528	*IGH::DUX4*
10310	Diagnosis	*DUX4*-r		11.04669621	*IGH::DUX4*
22418	Diagnosis	*JAK2*-r		4.582775114	Other
28627	Diagnosis	High Hyperdiploidy		4.421670495	Other
20756	Diagnosis	*PAX5* P80R		4.023154148	Other
11886	Diagnosis	*PAX5* alt		3.828213869	Other
27809	Diagnosis	High Hyperdiploidy		3.648299152	Other
22716	Diagnosis	High Hyperdiploidy		3.64458	Other
21795	Diagnosis	iAMP21-ALL		3.4793183	Other
23333	Diagnosis	*PAX5* alt		3.459461893	Other
22340	Diagnosis	iAMP21-ALL		3.3339267	Other
22466	Diagnosis	*ETV6-RUNX1*-like		3.261168593	Other
23003	Diagnosis	*KMT2A*-r		2.748345523	Other
27304	Diagnosis	*TCF3::PBX1*		2.694998638	Other
22153	Diagnosis	*PAX5* alt		2.559666783	Other
24390	Diagnosis	High Hyperdiploidy	*CRLF2*-r	2.467859316	Other
19732	Diagnosis	*PAX5* alt		2.446903569	Other
22352	Diagnosis	*PAX5* P80R		2.428692717	Other
9051	Diagnosis	*IKZF1* N159Y		2.42808051	Other
10184	Diagnosis	*ZNF384*-r		2.423395857	Other
10062	Diagnosis	*PAX5* alt		2.394014406	Other
22972	Diagnosis	*PAX5* alt		2.355714112	Other
23081	Diagnosis	*PAX5* alt		2.285266948	Other
28593	Diagnosis	High Hyperdiploidy		2.22167894	Other
28957	Diagnosis	High Hyperdiploidy		2.202978337	Other
21190	Diagnosis	*ETV6::RUNX1*		2.189155584	Other
23343	Diagnosis	*TCF3::PBX1*		2.185190114	Other
22922	Diagnosis	*BCL2/MYC*		2.183438129	Other
24371	Diagnosis	High Hyperdiploidy		2.182268318	Other
27354	Diagnosis	*TCF3::PBX1*		2.102422218	Other
25647	Diagnosis	Near haploidy		2.085459665	Other
27397	Diagnosis	*TCF3::PBX1*		2.070799583	Other
23110	Diagnosis	High Hyperdiploidy		1.752746827	Other
26681	Diagnosis	*ABL*-class		1.589294471	Other
29079	Diagnosis	High Hyperdiploidy		1.566750635	Other

24553	Diagnosis	*ETV6::RUNX1*		1.511158772	Other
23383	Diagnosis	iAMP21-ALL		1.364136166	Other
12910	Diagnosis	*PAX5* alt		1.250645155	Other
20874	Diagnosis	*PAX5* P80R		1.212072078	Other
11832	Diagnosis	*PAX5* alt		1.21185682	Other
21198	Diagnosis	*ETV6::RUNX1* -like		1.210592872	Other
22689	Diagnosis	*TCF3::PBX1*		1.202966584	Other
10656	Diagnosis	*PAX5* alt		1.196548854	Other
22584	Diagnosis	*PAX5* alt		1.191363535	Other
21466	Diagnosis	*ETV6::RUNX1* -like	Ph-like	1.171189201	Other
22964	Diagnosis	*PAX5* alt		1.167204513	Other
28959	Diagnosis	High Hyperdiploidy		1.158686831	Other
22722	Diagnosis	*BCR::ABL1*		1.146663615	Other
22572	Diagnosis	*IGH::IL3*		1.139723984	Other
23075	Diagnosis	*HLF* -r		1.137562385	Other
24848	Diagnosis	Other	*SH2B3* abnormality	1.120389734	Other
21318	Diagnosis	*KMT2A* -r		1.119293402	Other
24232	Diagnosis	*ETV6::RUNX1* -like		1.117367143	Other
23063	Diagnosis	*PAX5* alt		1.114983904	Other
23176	Diagnosis	*PAX5* alt		1.105335337	Other
23321	Diagnosis	*ETV6::RUNX1* -like		1.102301201	Other
22368	Diagnosis	*KMT2A* -r		1.096586971	Other
23273	Diagnosis	*PAX5* alt		1.095875208	Other
11060	Diagnosis	Low hypodiploidy		1.093486865	Other
22046	Diagnosis	*ZEB2/CEBP*		1.08836336	Other
24493	Diagnosis	*MEF2D* -r		1.08467127	Other
22327	Diagnosis	High Hyperdiploidy		1.083916541	Other
23353	Diagnosis	*JAK2* -r		1.083476372	Other
20323	Diagnosis	*ZNF384* -r		1.077892684	Other
24607	Diagnosis	*ZNF384* -r		1.071837094	Other
21424	Diagnosis	Other	*MAPK10* -r	1.068537097	Other
28982	Diagnosis	*ABL-* class		1.067598885	Other
27442	Diagnosis	*ABL-* class		1.066644429	Other
21532	Diagnosis	*NUTM1* -r		1.062617284	Other
22150	Diagnosis	*PAX5* P80R		1.056863238	Other
21693	Diagnosis	*PAX5* alt		1.048205183	Other
20317	Diagnosis	*ZEB2/CEBP*		1.044378326	Other
28956	Diagnosis	*BCR::ABL1*	High Hyperdiploidy	1.039865957	Other
27162	Diagnosis	High Hyperdiploidy		1.034799995	Other
19578	Diagnosis	iAMP21-ALL		0.855632657	Other
11553	Diagnosis	*TCF3::PBX1*		0.815723035	Other
25379	Diagnosis	*KMT2A* -r		0.808925039	Other
21940	Diagnosis	*KMT2A* -r		0.558055021	Other
20755	Diagnosis	*ETV6::RUNX1*		0	Other
22355	Diagnosis	*DUX4* -r	*ZEB2/CEBP*	0	Other
11959	Diagnosis	ABL-class		0	Other
12729	Diagnosis	*ETV6::RUNX1* -like		0	Other
19730	Diagnosis	*TCF3::PBX1*		0	Other
21487	Diagnosis	*IKZF1* N159Y	*PAX5* alt	0	Other
21694	Diagnosis	*IKZF1* N159Y		0	Other
22104	Diagnosis	*JAK2* -r		0	Other
22245	Diagnosis	*TCF3::PBX1*		0	Other
22980	Diagnosis	Other		0	Other
21321	Diagnosis	*ETV6::RUNX1* -like	*CRLF2* -r	0	Other
20161	Diagnosis	*PAX5* alt		0	Other
24419	Diagnosis	*ZEB2/CEBP*		0	Other
22621	Diagnosis	*ZNF384* -r		0	Other
12522	Diagnosis	*PAX5* alt		0	Other
20764	Diagnosis	*ETV6:RUNX1* -like		0	Other
22094	Diagnosis	*ZNF384* -r		0	Other
9850	Diagnosis	ABL-class	*MEF2D* -r	0	Other
23481	Diagnosis	*ZNF384* -r		0	Other
23813	Diagnosis	*ZEB2/CEBP*		0	Other
11148	Diagnosis	*DUX4* -r		0	Other
20706	Diagnosis	*PAX5* alt		0	Other
23515	Diagnosis	*ETV6::RUNX1* -like		0	Other
23881	Diagnosis	*PAX5* alt		0	Other
11149	Diagnosis	Other	*SPOP* abnormality	0	Other
23913	Diagnosis	*PAX5* alt		0	Other
11440	Diagnosis	*TCF3::PBX1*		0	Other
23560	Diagnosis	*PAX5* alt		0	Other
23919	Diagnosis	*CRLF2* -r		0	Other
22416	Diagnosis	High Hyperdiploidy		0	Other
23650	Diagnosis	*PAX5* alt		0	Other
23936	Diagnosis	*PAX5* alt		0	Other
23673	Diagnosis	*ETV6::RUNX1* -like		0	Other
24041	Diagnosis	*PAX5* alt		0	Other
21800	Diagnosis	*PAX5* alt		0	Other
23445	Diagnosis	*DUX4* -r		0	Other
23678	Diagnosis	Other	*TCF3* -r, chromosomal gain	0	Other
24079	Diagnosis	*MEF2D* -r		0	Other
26772	Diagnosis	*KMT2A* -r		0	Other
24680	Diagnosis	*CRLF2* -r		0	Other
12908	Diagnosis	*PAX5* p.P80R		0	Other
12911	Diagnosis	*MEF2D* -r		0	Other
24082	Diagnosis	*ZNF384* -r		0	Other
20315	Diagnosis	*TCF3::PBX1*	*PAX5* alt	0	Other
10442	Diagnosis	iAMP21-ALL		0	Other
22188	Diagnosis	Other	*CNTNAP3B* abnormality, *MAPKAP1* -r	0	Other
24456	Diagnosis	*ZNF384* -r		0	Other
10927	Diagnosis	*ETV6::RUNX1* -like		0	Other
11062	Diagnosis	*BCL2/MYC*		0	Other
22660	Diagnosis	ABL-class		0	Other
24100	Diagnosis	*PAX5* alt		0	Other
24566	Diagnosis	*ZNF384* -r		0	Other
20628	Diagnosis	ABL-class		0	Other
24601	Diagnosis	*ZNF384* -r		0	Other

24262	Diagnosis	*PAX5* alt		0	Other
24669	Diagnosis	Other	*NOTCH2* deletion	0	Other
8451	Diagnosis	*MEF2D* -r		0	Other
12641	Diagnosis	*TCF3::PBX1*		0	Other
23922	Diagnosis	*ETV6::RUNX1*		0	Other
24332	Diagnosis	*PAX5* alt		0	Other
12373	Diagnosis	High Hyperdiploidy		0	Other
22367	Diagnosis	*ETV6::RUNX1* -like		0	Other
21869	Diagnosis	*CRLF2* -r		0	Other
24338	Diagnosis	*IGH::ID4*		0	Other
10868	Diagnosis	Other	*CNTNAP3B* abnormality, chromosomal gain	0	Other
21342	Diagnosis	*CRLF2* -r		0	Other
8878	Diagnosis	*ZNF384* -r		0	Other
20653	Diagnosis	*ZNF384* -r		0	Other
11734	Diagnosis	*CRLF2* -r		0	Other
20447	Diagnosis	*ETV6::RUNX1*		0	Other
20951	Diagnosis	*ETV6::RUNX1*		0	Other
10740	Diagnosis	High Hyperdiploidy		0	Other
10194	Diagnosis	*BCR::ABL1*		0	Other
21708	Diagnosis	Near haploidy		0	Other
7653	Diagnosis	Near haploidy		0	Other
24284	Diagnosis	Low hypodiploidy		0	Other
12202	Diagnosis	Near haploidy		0	Other
19732	Germline			9.609084521	Germline
24419	Germline			5.148393325	Germline
21322	Germline			5.095756604	Germline
22621	Germline			4.929102403	Germline
11886	Germline			4.90298418	Germline
22972	Germline			4.845098319	Germline
22964	Germline			4.643281619	Germline
23769	Germline			4.628836738	Germline
12820	Germline			4.589223408	Germline
26681	Germline			4.275603165	Germline
28593	Germline			3.470147211	Germline
9469	Germline			3.415351249	Germline
23003	Germline			3.319718592	Germline
24390	Germline			3.311850014	Germline
24553	Germline			3.161975966	Germline
12522	Germline			2.478541807	Germline
23333	Germline			2.457615702	Germline
23383	Germline			2.431042353	Germline
20161	Germline			2.408325471	Germline
9051	Germline			2.398747806	Germline
22046	Germline			2.391577042	Germline
23176	Germline			2.351520227	Germline
20764	Germline			2.350764711	Germline
21795	Germline			2.346292171	Germline
9850	Germline			2.325097353	Germline
21532	Germline			2.312117136	Germline
12134	Germline			2.308665854	Germline
22006	Germline			2.303942388	Germline
11832	Germline			2.295039216	Germline
22722	Germline			2.265144988	Germline
23913	Germline			2.258603353	Germline
20724	Germline			2.256294729	Germline
10656	Germline			2.229737916	Germline
27162	Germline			2.211805413	Germline
27809	Germline			2.211310508	Germline
23321	Germline			2.209546979	Germline
27442	Germline			2.193431068	Germline
21437	Germline			2.176210237	Germline
10442	Germline			2.171603873	Germline
11062	Germline			2.159751501	Germline
11440	Germline			2.158943493	Germline
23842	Germline			2.13684745	Germline
22417	Germline			2.099779751	Germline
24232	Germline			2.097814318	Germline
29079	Germline			2.094646969	Germline
28956	Germline			2.07690125	Germline
21940	Germline			2.039517036	Germline
22224	Germline			1.682946538	Germline
19578	Germline			1.672482819	Germline
20515	Germline			1.629332978	Germline
20951	Germline			1.370521328	Germline
28957	Germline			1.236871391	Germline
28627	Germline			1.145556299	Germline
27354	Germline			1.119714605	Germline
20447	Germline			1.111528038	Germline
28982	Germline			1.083171155	Germline
21190	Germline			1.077650396	Germline
22368	Germline			1.030736512	Germline
22340	Germline			0.898896752	Germline
23110	Germline			0.857317751	Germline
24284	Germline			0.851975963	Germline
22355	Germline			0.844857742	Germline
25379	Germline			0.836254909	Germline
7653	Germline			0.832765446	Germline
10194	Germline			0.708281816	Germline
12335	Germline			0.532095757	Germline
10310	Germline			0	Germline
11957	Germline			0	Germline
11959	Germline			0	Germline
12118	Germline			0	Germline
12334	Germline			0	Germline
12356	Germline			0	Germline
12729	Germline			0	Germline
19730	Germline			0	Germline

20683	Germline			0	Germline
20720	Germline			0	Germline
21487	Germline			0	Germline
21689	Germline			0	Germline
21694	Germline			0	Germline
22037	Germline			0	Germline
22104	Germline			0	Germline
22245	Germline			0	Germline
22346	Germline			0	Germline
22405	Germline			0	Germline
22980	Germline			0	Germline
20755	Germline			0	Germline
27397	Germline			0	Germline
27304	Germline			0	Germline
26772	Germline			0	Germline
28959	Germline			0	Germline
25647	Germline			0	Germline
22045	Germline			0	Germline
22918	Germline			0	Germline
23481	Germline			0	Germline
10925	Germline			0	Germline
20317	Germline			0	Germline
22922	Germline			0	Germline
23507	Germline			0	Germline
23813	Germline			0	Germline
11148	Germline			0	Germline
20706	Germline			0	Germline
22065	Germline			0	Germline
23515	Germline			0	Germline
23881	Germline			0	Germline
11149	Germline			0	Germline
21230	Germline			0	Germline
22150	Germline			0	Germline
23273	Germline			0	Germline
23533	Germline			0	Germline
21424	Germline			0	Germline
22354	Germline			0	Germline
23560	Germline			0	Germline
23919	Germline			0	Germline
11556	Germline			0	Germline
22416	Germline			0	Germline
23343	Germline			0	Germline
23650	Germline			0	Germline
23936	Germline			0	Germline
21693	Germline			0	Germline
22804	Germline			0	Germline
23353	Germline			0	Germline
23673	Germline			0	Germline
24041	Germline			0	Germline
11672	Germline			0	Germline
21800	Germline			0	Germline
22897	Germline			0	Germline
23445	Germline			0	Germline
23678	Germline			0	Germline
24079	Germline			0	Germline
12910	Germline			0	Germline
20874	Germline			0	Germline
22153	Germline			0	Germline
22716	Germline			0	Germline
23863	Germline			0	Germline
10062	Germline			0	Germline
21198	Germline			0	Germline
11053	Germline			0	Germline
10184	Germline			0	Germline
21321	Germline			0	Germline
22418	Germline			0	Germline
23074	Germline			0	Germline
11178	Germline			0	Germline
20035	Germline			0	Germline
23081	Germline			0	Germline
11553	Germline			0	Germline
22572	Germline			0	Germline
20716	Germline			0	Germline
21466	Germline			0	Germline
22584	Germline			0	Germline
20756	Germline			0	Germline
12816	Germline			0	Germline
22094	Germline			0	Germline
22689	Germline			0	Germline
12460	Germline			0	Germline
23075	Germline			0	Germline
24848	Germline			0	Germline
24680	Germline			0	Germline
12911	Germline			0	Germline
24082	Germline			0	Germline
10186	Germline			0	Germline
20315	Germline			0	Germline
24391	Germline			0	Germline
24423	Germline			0	Germline
22188	Germline			0	Germline
24456	Germline			0	Germline
10927	Germline			0	Germline
22466	Germline			0	Germline
22660	Germline			0	Germline
24607	Germline			0	Germline
24100	Germline			0	Germline
24493	Germline			0	Germline

8878	Germline			0	Germline
20653	Germline			0	Germline
24566	Germline			0	Germline
11060	Germline			0	Germline
11811	Germline			0	Germline
20628	Germline			0	Germline
11734	Germline			0	Germline
24601	Germline			0	Germline
20696	Germline			0	Germline
12641	Germline			0	Germline
23114	Germline			0	Germline
24262	Germline			0	Germline
24669	Germline			0	Germline
8451	Germline			0	Germline
23063	Germline			0	Germline
23922	Germline			0	Germline
21568	Germline			0	Germline
10876	Germline			0	Germline
22387	Germline			0	Germline
24332	Germline			0	Germline
12373	Germline			0	Germline
22367	Germline			0	Germline
19827	Germline			0	Germline
21869	Germline			0	Germline
24338	Germline			0	Germline
20323	Germline			0	Germline
23078	Germline			0	Germline
10868	Germline			0	Germline
21342	Germline			0	Germline
24371	Germline			0	Germline
23132	Germline			0	Germline
12083	Germline			0	Germline
22327	Germline			0	Germline
21318	Germline			0	Germline
10740	Germline			0	Germline
21708	Germline			0	Germline
12202	Germline			0	Germline

Supplementary Table 7: *IGH::DUX4* breakpoint mapping. Contig/scaffold ID - contigs (termed C#) and scaffolds were generated from the de novo alignment of paired-reads that mapped to the *IGH* or *DUX4* locus. Each row contains new information for a contig or scaffold. In most cases, each row represents a new contig/scaffold per row but there are some examples with two rows per contig/scaffold as it maps to two independent positions within the *IGH* or *DUX4* locus i.e. LP2000156-DNA_A01 in which scaffold 1 has two alignments to the *IGH* locus (chr14:105863102-105863261 (309-471bp within scaffold 1) and chr14:105862866-105862956 (615-705bp within scaffold 1)). Number of different regions within one contig/scaffold that align to the *IGH* locus on chr14 are given. The length and section (in bp) of the contig/scaffold that maps to the *IGH* locus are given. The start and end position of the genomic co-ordinates within the *IGH* locus to which the contig/scaffold align to. The genomic sequence described on the forward or reverse strand and the accuracy (%) of the alignment. Alignment information for the *DUX4* locus on chr4. A range of potential start and end positions are provided as the contig/scaffold often mapped to multiple locations with the repeat array of *DUX4* gene copies. Similarly, some contigs/scaffolds mapped with the same degree of accuracy to the *DUX4* homologs on chr4 and chr10. Alignment information for the *DUX4* locus on chr10 is given.

Unique ID	Scaffold / contig ID	*IGH* breakpoint count	Chr	Chr14 - query start	Chr14 - query end	Chr14 - orientation	Chr14 - % Identity	Chr14: start-end position of contig	Chr4 - *DUX4* breakpoint range	Chr4 - orientation	Chr4 - *DUX4* breakpoint count	Chr4 - % identity	Chr4: start-end position of contig	Chr10 - DUX4 breakpoint range	Chr10 - orientation	Chr10 - *DUX4* breakpoint count	Chr10 - % identity	Chr10: start-end position of contig
10186	C1	1	14	105863990	105864256	-	99.625	484-750bp						chr10:133664641-133761094	+	14	100	1-438bp
10186	C3	1	14	105862856	105863258	-	98.759	469-871bp						chr10:133666811-133756681	-	7	100	1-464bp
10310	scaffold1	1	14	105863993	105864196	+	99.51	1-204bp	chr4:190067376-190173416	+	7	100	368-584bp	chr10:133666571-133756194	+	7	100	368-584bp
10876	C1	1	14	106349284	106349854	-	96.497	5-575bp						chr10:133666362-133756191	+	7	100	583-1005bp
10925	C1	1	14	105863947	105864252	-	99.02	353-658bp						chr10:133665949-133759016	-	12	100	1-351bp
10925	C3	1	14	106606116	106606439	+	100	547-870bp						chr10:133664573-133761099	+	14	100	1-511bp
11053	C1	1	14	105863952	105864247	-	98.986	285-580bp	chr4:190067345-190173425	-	7	100	1-257bp	chr10:133666540-133756203	-	7	100	1-257bp
11053	C3	1	14	106001535	106002042	-	100	1-508bp	chr4:190071452-190174416	-	8	100	514-936bp	chr10:133670670-133760077	-	8	100	514-936bp
11178	C1	1	14	105862839	105863255	+	98.801	1-417bp	chr4:190068667-190174934	-	8	99.767	425-853bp					
11178	C3	1	14	105904685	105905187	+	100	482-924bp	chr4:190067695-190091226	-	6	99.784	1-462bp					
11556	C1	1	14	105864209	105864616	-	100	478-885bp						chr10:133666047-133759238	-	12	100	1-475bp
11556	C3	1	14	106657728	106658198	+	100	477-947bp						chr10:133664646-133761120	+	14	98.911	1-459bp
11672	C3	1	14	105905723	105906247	-	99.429	1-524bp						chr10:133665982-133759009	-	12	100	524-834bp
11672	C5	1	14	105860602	105861042	-	100	499-839bp						chr10:133667552-133754148	+	2	100	1-500bp
11811	scaffold1	1	14	105862878	105863207	-	99.697	358-687bp	chr4:190022550-190173889	-	8	99.462	1-186bp					
11811	C29								chr4:190070929-190087675	-	3	98.905	1-274bp					
11811	C33	1	14	106592676	106593183	+	100	508-1015bp	chr4:190068695-190175028	+	8	99.798	1-495bp					
11957	scaffold1	1	14	105862834	105863251	-	98.804	335-752bp	chr4:190067868-190090739	-	8	99.5	1-200bp					
11957	C15	1	14	106762094	106762120	+	100	446-472bp	chr4:190082664-190092959	+	3	100	1-403bp					
12083	C1	1	14	105863965	105864255	-	98.969	298-588bp						chr10:133666842-133756530	-	7	100	1-282bp
12083	C3	1	14	106657728	106658224	-	99.396	1-497bp						chr10:133664573-133761079	-	14	100	517-1007bp
12118	C1	1	14	105864325	105864633	-	100	14-322bp										
12118	C3								chr4:190065418-190065797	-	1	99.737	1-379bp					
12118	C5	1	14	106107973	106108382	-	100	1-410bp	chr4:190070329-190173324	+	8	100	415-877bp					
12134	C9								chr4:190068612-190091936	+	8	99.608	1-255bp					
12134	C11	1	14	105883925	105884361	+	99.542	489-925bp	chr4:190080062-190083828	-	2	100	1-473bp					
12334	C5	1	14	106657723	106658066	+	100	520-863bp	chr4:190068710-190175030	+	8	99.585	1-482bp					
12356	C9								chr4:190067695-190067847	-	1	99.346	1-153bp					
12356	C11								chr4:190065385-190065605	+	1	100	5-225bp					
12356	C15	1	14	105863991	105864256	+	98.872	1-266bp	chr4:190067524-190173391	+	7	100	286-329bp	chr10:133666719-133759479	+	12	100	286-329bp
12356	C17	1	14	106592695	106592825	+	100	296-426bp	chr4:190068884-190174988	+	8	100	1-266bp					
12460	C7	2	14	105862293	105862675	-	100	528-910bp	chr4:190073798-190090667	-	5	99.746	1-393bp	chr10:133666409-133756208	-	7	99.746	1-393bp
12460	C7	2	14	106359678	106359790	+	99.115	311-510bp	chr4:190073798-190090667	-	5	99.746	1-393bp	chr10:133666409-133756208	-	7	99.746	1-393bp
12460	C9	1	14	106359192	106359659	+	100	1-468bp						chr10:133664558-133761052	-	14	99.374	547-1025bp
12816	C3	1	14	105863449	105863855	+	99.754	1-407bp	chr4:190067393-190173457	+	7	99.585	432-672bp	chr10:133666588-133756235	+	7	99.585	432-672bp
12816	C5	1	14	106657737	106658279	-	100	1-543bp						chr10:133664526-133761072	-	14	100	555-1085bp
12820	C1								chr4:190065374-190065585	-	1	99.057	1-210bp					
12820	C3	1	14	105863936	105864247	+	99.038	1-312bp	chr4:190072759-190092942	+	7	99.757	315-725bp					
12820	C5	2	14	106810442	106810929	-	100	1-488bp	chr4:190082109-190082377	-	1	100	530-789bp	chr10:133664832-133761116	-	14	100	530-798bp
12820	C5	2	14	105886037	105886061	-	100	490-514bp	chr4:190082109-190082377	-	1	100	530-789bp	chr10:133664832-133761116	-	14	100	530-798bp
19827	C5	1	14	105862544	105863095	-	99.819	286-837bp	chr4:190067372-190173463	-	7	100	1-268bp	chr10:133666567-133756241	-	7	100	1-268bp
20035	scaffold1	1	14	105864427	105864634	+	100	1-208bp	chr4:190069031-190175007	-	9	100	296-433bp	chr10:133664931-133761084	-	14	100	296-433bp
20035	scaffold2	1	14	105862931	105863295	+	98.63	1-365bp	chr4:190068671-190174878	-	8	99.729	442-810bp					
20515	scaffold1	1	14	105863190	105863601	+	99.029	1-412bp	chr4:190022776-190022266	-	1	99.413	569-1079bp					
20515	C17	1	14	105905386	105905988	-	99.834	1-603bp	chr4:190174202-190174589	-	1	99.742	603-990bp	chr10:133671265-133760285	-	10	99.742	603-990bp
20683	C1	1	14	105861075	105861490	+	99.76	1-416bp	chr4:190080831-190081242	+	1	100	412-823bp					
20683	C3	1	14	105861481	105861942	-	99.784	1-462bp	chr4:190065382-190174519	-	2	100	461-865bp	chr10:133671195-133760602	-	10	100	461-865bp
20696	C3	1	14	105923189	105923535	-	99.712	408-754bp	chr4:190070991-190081267	-	2	100	1-391bp					
20696	C5	2	14	105861966	105862081	+	100	1-116bp	chr4:190071748-190091951	-	7	99.536	411-841bp					
20696	C5	2	14	105862071	105862354	-	100	124-407bp	chr4:190071748-190091951	-	7	99.536	411-841bp					
20716	C1	1	14	105861788	105862254	+	100	1-467bp	chr4:190174685-190174192	-	1	99.798	466-959bp	chr10:133670868-133760768	-	10	99.798	466-959bp
20720	C1	1	14	105863808	105864256	-	100	503-951bp						chr10:133633337-133632836	-	1	100	1-502bp

20724	C1	1	14	105863430	105863862	-	99.769	471-903bp						chr10:133666896-133760095	-	12	97.773	1-492bp
21230	C5	1	14	105862646	105862964	-	100	57-375bp										
21230	C7								chr4:190174336-190174713	+	1	100	1-378bp	chr10:133667713-133760796	+	12	100	1-378bp
21230	C9	1	14	105893572	105894094	-	99.809	1-523bp	chr4:190071054-190081454	+	2	100	518-1032bp					
21322	scaffold1	1	14	106268624	106268899	+	99.638	333-608bp	chr4:190068633-190174472	+	9	100	1-189bp	chr10:133664539-133760743	+	14	100	1-189bp
21322	C47	1	14	105904520	105904837	-	100	1-318bp	chr4:190065813-190174983	-	10	97.5	320-359bp	chr10:133665005-133761060	-	14	97.5	320-359bp
21437	C1								chr4:190065754-190065485	-	1	99.63	1-269bp					
21437	C3	1	14	105863559	105863859	-	99.668	14-314bp										
21437	C5	2	14	106257763	106258164	-	100	1-402bp	chr4:190070369-190090487	+	7	100	440-788bp					
21437	C5	2	14	105888555	105888575	-	100	408-428bp	chr4:190070369-190090487	+	7	100	440-788bp					
21568	C1	1	14	105864690	105865242	+	100	1-553bp	chr4:190079907-190083685	+	2	100	590-1074bp					
21689	scaffold1	1	14	105863990	105864174	-	99.459	391-575bp	chr4:190067770-190173605	-	9	100	1-100bp	chr10:133666986-133759692	-	12	1-100bp	
21689	scaffold2	1	14	106268607	106268913	-	99.674	1-307bp	chr4:190068798-190175019	-	8	100	411-793bp					
22006	C1	1	14	105901893	105902300	+	100	446-853bp						chr10:133666713-133756568	-	7	99.332	1-446bp
22037	scaffold1	2	14	105863997	105864217	+	99.548	1-221bp	chr4:190067335-190173418	+	7	100	408-667bp	chr10:133666530-133755937	+	7	100	408-667bp
22037	scaffold1	2	14	105864218	105864260	+	100	328-370bp	chr4:190067335-190173418	+	7	100	408-667bp	chr10:133666530-133755937	+	7	100	408-667bp
22037	C9	1	14	106657736	106658130	-	99.494	1-395bp						chr10:133664637-133761116	-	14	100	410-873bp
22045	C1	1	14	105863798	105864252	-	99.341	364-818bp						chr10:133667114-133756521	-	7	97.878	1-374bp
22045	C3	1	14	106657724	106658230	-	99.291	1-507bp						chr10:133664651-133761097	-	14	100	529-959bp
22065	C1	1	14	105884543	105884873	+	100	1-331bp	chr4:190068805-190175024	-	8	100	351-731bp					
22065	C3	1	14	105939755	105940204	-	100	1-450bp	chr4:190022353-190174086	+	8	99.415	503-844bp	chr10:133666993-133759750	+	12	100	849-900bp
22065	C3	1	14	105939755	105940204	-	100	1-450bp	chr4:190067777-190090898	-	8	100	849-900bp	chr10:133666993-133759750	-	12	100	849-900bp
22346	scaffold1	2	14	105863102	105863261	-	96.933	309-471bp	chr4:190067820-190173758	-	9	100	1-100bp					
22346	scaffold1	2	14	105862866	105862956	-	100	615-705bp	chr4:190067820-190173758	-	9	100	1-100bp					
22346	C13	1	14	106802693	106803023	+	100	424-754bp	chr4:190068764-190174985	+	8	100	1-383bp					
22354	C1	1	14	105864827	105865246	-	100	471-890bp	chr4:190084125-190091179	-	2	100	1-462bp					
22354	C3	1	14	106802692	106803056	-	100	1-365bp	chr4:190071875-190092184	-	6	100	402-938bp					
22387	scaffold1	1	14	105862334	105862553	-	100	408-627bp	chr4:190068835-190174853	+	8	100	1-180bp					
22387	scaffold2	2	14	105914553	105914966	+	99.759	442-856bp	chr4:190082494-190082774	-	1	100	1-281bp					
22387	scaffold2	2	14	105914553	105914966	+	99.759	442-856bp	chr4:190069124-190175078	-	9	100	316-431bp	chr10:133665024-133761155	-	14	100	316-431bp
22387	C13								chr4:190072906-190092826	+	8	98.649	3-150bp					
22405	C3	1	14	105864897	105865244	+	100	1-348bp	chr4:190070377-190090488	+	7	100	375-716bp					
22405	C5	1	14	106627251	106627670	+	100	428-847bp	chr4:190068718-190174969	+	8	100	1-413bp					
22804	C5	1	14	105912263	105912666	-	99.01	1-404bp	chr4:190069128-190175012	-	9	100	408-453bp	chr10:133665028-133761089	-	14	100	408-453bp
22804	C7	1	14	106592706	106593116	+	100	63-473bp	chr4:190069128-190175012	+	9	100	1-46bp	chr10:133665028-133761089	+	14	100	1-46bp
22804	C9								chr4:190068646-190175012	-	8	100	6-533bp					
22804	C11	1	14	105864204	105864635	+	100	1-432bp	chr4:190067509-190173465	+	7	100	437-569bp	chr10:133666704-133756243	+	7	100	437-569bp
22897	C3	1	14	105862610	105863057	-	100	301-748bp						chr10:133668884-133758590	-	6	100	1-300bp
22897	C5	1	14	106628583	106629121	+	99.258	550-1088bp						chr10:133664539-133761104	+	14	99.455	1-550bp
22918	C1	1	14	105863099	105863299	+	98.01	56-256bp	chr4:189930025-189930081	+	1	100	1-57bp					
22918	C3								chr4:190082118-190082364	+	1	99.595	1-247bp	chr10:133664841-133761103	+	14	99.595	1-247bp
22918	C3								chr4:189929912-189929955	+	1	97.727	261-304bp	chr10:133664841-133761103	+	14	99.595	1-247bp
22918	C5	1	14	105882996	105883418	+	99.291	446-868bp	chr4:190174244-190174683	-	1	100	1-440bp	chr10:133670920-133760327	-	10	100	1-440bp
23074	scaffold1	2	14	105863862	105864187	+	100	1-326bp	chr4:190068756-190174965	-	8	100	471-841bp					
23074	scaffold1	2	14	105864188	105864248	+	100	380-440bp	chr4:190068756-190174965	-	8	100	471-841bp					
23078	scaffold1	1	14	105862785	105863235	-	99.557	360-810bp	chr4:190174445-190174711	+	1	99.625	1-267bp	chr10:133664512-133760794	+	14	99.625	1-267bp
23078	scaffold2	2	14	105897960	105897987	+	100	525-552bp	chr4:190086506-190090324	-	2	99.617	1-522bp					
23078	scaffold2	2	14	105897988	105898512	+	99.81	587-1111bp	chr4:190086506-190090324	-	2	99.617	1-522bp					
23078	C11	1	14	106268615	106268719	-	100	1-105bp	chr4:190066741-190093123	+	9	100	130-150bp	chr10:133665937-133758674	+	12	100	130-150bp
23114	C3	1	14	105863073	105863240	-	98.81	507-674bp						chr10:133666835-133756741	-	7	99.6	1-499bp
23114	C5	1	14	106675016	106675477	+	100	554-1015bp						chr10:133664565-133761108	+	14	100	1-528bp
23132	scaffold1	1	14	105863798	105863842	+	97.778	260-304bp						chr10:133666550-133756417	+	7	99.783	314-774bp
23132	scaffold2	2	14	105844788	105845142	-	100	1-355bp						chr10:133665685-133758782	-	12	100	519-899bp
23132	scaffold2	2	14	105844703	105844773	-	100	434-504bp						chr10:133665685-133758782	-	12	100	519-899bp
23132	scaffold3	1	14	105862172	105862578	+	100	1-407bp	chr4:190174304-190174715	-	1	100	536-947bp	chr10:133667681-133760798	-	12	100	536-947bp
23132	C17	1	14	105906401	105906867	+	100	443-909bp						chr10:133664957-133761402	-	14	100	1-430bp
23507	scaffold1	1	14	105915203	105915756	+	99.819	505-1058bp	chr4:190174154-190174637	+	1	99.794	1-485bp	chr10:133670830-133760720	+	10	99.794	1-485bp
23507	C5	1	14	105861731	105862225	+	99.192	1-495bp	chr4:190065455-190174725	-	10	100	486-624bp	chr10:133664654-133760808	-	14	100	486-624bp
23507	C7	1	14	105862851	105863250	-	99.75	383-782bp	chr4:190073892-190173469	-	6	100	1-338bp	chr10:133666503-133756247	-	7	100	
23533	C1	1	14	105863917	105864259	+	99.125	1-343bp	chr4:190084126-190091220	+	2	99.801	346-847bp					

23533	C3	1	14	105916829	105917233	-	99.753	1-405bp	chr4:190068633-190174908	-	8	100	417-853bp					
23769	C1	2	14	106606125	106606607	-	99.793	1-483bp	chr4:190074344-190091790	-	5	100	509-1475bp					
23769	C1	2	14	105863451	105863856	-	99.507	1473-1868bp	chr4:190074344-190091790	-	5	100	509-1475bp					
23842	scaffold1	1	14	105862883	105863166	+	99.296	1-284bp						chr10:133664700-133760954	-	14	100	372-610bp
23842	scaffold2	2	14	105895644	105895666	+	100	506-528bp	chr4:190070405-190090530	-	7	99.157	1-356bp	chr10:133666309-133759381	-	12	99.157	1-356bp
23842	scaffold2	2	14	106335093	106335407	+	98.73	531-845bp	chr4:190070405-190090530	-	7	99.157	1-356bp	chr10:133666309-133759381	-	12	99.157	1-356bp
23863	scaffold2	2	14	105862574	105862882	+	99.676	389-697bp						chr10:133666504-133756191	+	7	98.577	1-281bp
23863	scaffold2	2	14	105862574	105862882	+	99.676	389-697bp						chr10:133650114-133650512	+	1	98.511	748-1150bp
23863	C13	1	14	105862137	105862545	+	99.511	1-408bp						chr10:133666803-133756620	+	7	99.27	451-858bp
24391	C3	1	14	105863936	105864249	+	98.726	1-314bp	chr4:190068698-190174990	-	8	100	316-769bp					
24391	C5	1	14	105886034	105886436	+	100	416-818bp	chr4:190083987-190090580	-	2	99.5	1-400bp					
24423	scaffold1	1	14	105862475	105862814	+	100	419-758bp	chr4:190066666-190089964	-	5	98.734	1-237bp					
24423	C1								chr4:190082945-190093017	+	3	100	15-194bp					
24423	C7	1	14	105861712	105862150	-	100	411-849bp	chr4:190174306-190174718	+	1	100	1-413bp	chr10:133667683-133760801	+	12	100	1-413bp

Supplementary Table 8: Genetic variants detected by WGS involving nine genes/regions. The genes/regions are CNA screened for by MLPA for their incorporation into the UKALL-CNA classifier, used for risk-directed treatment stratification.[1] Details of each genetic variant detected by MLPA per patient sample is provided. The type of genetic variant and the level of evidence (SV or CNA) observed by WGS is provided. SV or CNA evidence of a variant was categorised: [A] somatic SV/CNA reported by Manta/Canvas and passed filtering procedures. [B] Somatic SV/CNA reported by Manta/Canvas that did not pass filtering procedures. [C] SV identified through discordantly mapped paired-reads in the bam file. [D] Germline SV/CNV reported by Manta/Canvas and passed filtering procedures. [E] No evidence of germline or somatic SV/CNA in the aligned reads. Evidence included; DEL=deletion [CN=1] (copy number =1) unless otherwise stated, GAIN=gain [CN=3] (copy number=3) unless otherwise stated, DUP=duplication, LOSS=same as deletion {CN=1} copy number =1, unless otherwise stated. The majority (332/367) of CNA involved genes used in the UKALL-CNA classifier or within *ERG*; the remaining CNA involved whole chromosome/chromosome arm gains (n=35) which are not used in the UKCNA-ALL classifier (highlighted in grey). Nine CNA were identified by MLPA only (highlighted in dark grey). A summary of agreement between the two methods for key CNA and the main reason for disagreement is provided. 'Single probe CNA' defines genetic abnormalities that affect a single exon/region not included/included in the MLPA kit or genetic abnormalities that affect multiple exons covered by a single MLPA probe (highlighted in green). 'Not detected by MLPA' indicates genetic abnormalities that did not pass the MLPA detection threshold (≤0.75). Ph-like definition in this table indicates abnormalities that drive the unique GEP of the subtype (Ph-like) that is defined by WTS.

Unique ID	WGS subgroup	Other WGS subgroups	Genetic abnormality detected by MLPA	Genetic abnormality detected by WGS	SV evidence	CNA evidence	Agreement (WGS and MLPA)	Reason for disagreement
7653	Near haploidy			Chromosome X CNN-LOH		[A] CNN-LOH:chrX [CN=2]	No	MLPA not done
8451	*MEF2D*-r		*CDKN2A/B* DEL [CN=0]	*CDKN2A/B* DEL		[A] LOSS:chr9:21159209-22502764 [CN=0]	Yes	
8451	*MEF2D*-r		*PAX5* Whole DEL	*PAX5* DEL		[A] LOSS:chr9:22507608-39047309	Yes	
8451	*MEF2D*-r		*RB1* Whole DEL	*RB1* DEL		[A] LOSS:chr13:30475758-114350837	Yes	
8878	*ZNF384*-r		*ETV6* Whole DEL	*ETV6* DEL		[A] LOSS:chr12:6688403-15047462	Yes	
9051	*IKZF1* N159Y			*IKZF1* Ex5 DUP	[A] DUP:chr7:50,382,361-50,382,856		No	Single probe CNA
9469	*DUX4*-r		*CDKN2B* Ex2 DEL	*CDKN2A/B* DEL	[A] DEL:chr9:21,827,633-22,008,353		Yes	
9469	*DUX4*-r		*ERG* Ex5-6 DEL	*ERG* Ex5-6 DEL	(A) DEL:chr21:38,391,955-38,439,565		Yes	
9469	*DUX4*-r		*IKZF1* Ex4-7 DEL	IKZF1 Ex4-7 DEL	[A] DEL:chr7:50,345,147-50,395,986		Yes	
9850	ABL-class			*PAX5* Ex2-6 DEL	[A] DEL:chr9:36,929,004-37,030,754		No	Not detected by MLPA
9850	ABL-class			*PAX5* Ex1 DEL	[A] DEL:chr9:37,025,247-37,421,039		No	Single probe CNA
9850	ABL-class		*CDKN2A* Ex2 DEL [CN=0]	*CDKN2A* DEL	[A] DEL:chr9:21,911,911-21,992,859		Yes	
9850	ABL-class		*CDKN2A/B* DEL	*CDKN2A/B* DEL	[A] DEL:chr9:21,973,581-22,008,355		Yes	
9850	ABL-class		*IKZF1* Ex1-7 DEL	*IKZF1* Ex2-7 DEL	[A] DEL:chr7:50,307,333-50,395,985		Yes	
10062	*PAX5* alt			*ETV6* Ex6-8 DEL	[A] DEL:chr12:11,883,804-11,917,092		No	Single probe CNA
10062	*PAX5* alt			*PAX5* intron 1 DEL	[A] DEL:chr9:37,025,241-37,030,754		No	Single probe CNA
10062	*PAX5* alt		*CDKN2A/B* DEL [CN=0]	*CDKN2A/B* DEL	[A] DEL:chr9:21,902,129-22,008,353	[A] LOSS:chr9:21891627-22008339 [CN=0]	Yes	
10062	*PAX5* alt		*IKZF1* Ex4-7 DEL	*IKZF1* Ex4-7 DEL	[A] DEL:chr7:50,345,142-50,395,981	[A] LOSS:chr7:50344592-50395906	Yes	
10062	*PAX5* alt		*PAX5* Whole DEL	*PAX5* DEL		[A] LOSS:chr9:22008340-39838758	Yes	
10184	*ZNF384*-r		*BTG1* Ex2 DEL	*BTG1* Ex2 DEL		[A] LOSS:chr12:91,884,003-92,144,377	Yes	
10186	*DUX4*-r						NA	
10194	*BCR::ABL1*		*CDKN2A/B* DEL	*CDKN2A/B* DEL	[A] DEL:chr9:21,970,982-21,994,136		Yes	
10194	*BCR::ABL1*		*IKZF1* Whole DEL	*IKZF1* DEL		[A] LOSS:chr7:40502-56106559	Yes	
10194	*BCR::ABL1*		*PAX5* Whole DEL	*PAX5* DEL		[A] LOSS:chr9:113141-38773594	Yes	
10310	*DUX4*-r						NA	
10442	iAMP21-ALL		*ETV6* Whole DEL	*ETV6* DEL		[A] LOSS:chr12:79009-19757425	Yes	
10442	iAMP21-ALL		*IKZF1* Whole DEL	*IKZF1* DEL		[A] LOSS:chr7:50105060-50434561 [CN=0]	Yes	
10442	iAMP21-ALL		*PAX5* Ex1-6 GAIN	*PAX5* Ex1-7 DUP	[A] DUP:chr9:36,915,692-37,259,721		Yes	
10656	*PAX5* alt		*CDKN2A/B* DEL	*CDKN2A/B* DEL		[A] LOSS:chr9:113593-40846161	Yes	
10656	*PAX5* alt		*IKZF1* Whole DEL	*IKZF1* DEL		[A] LOSS:chr7:39589538-51478667	Yes	
10656	*PAX5* alt		*PAX5* Whole DEL	*PAX5* DEL		[A] LOSS:chr9:113593-40846161	Yes	
10740	High Hyperdiploidy		PAR1 GAIN	Chromosome X GAIN		[A] GAIN:chrX [CN=3]	Yes	
10868	Other	CNTNAP3B-C20orf203, chromosomal gain	*CDKN2A* DEL [CN=0]	*CDKN2A* DEL	[A] DEL:chr9:21,825,511-21,993,868		Yes	
10868	Other	CNTNAP3B-C20orf203, chromosomal gain	*CDKN2B* DEL	*CDKN2A/B* DEL		[A] LOSS:chr9:73333-38773443	Yes	
10868	Other	CNTNAP3B-C20orf203, chromosomal gain	PAR1 GAIN	Chromosome X GAIN		[A] GAIN:chrX [CN=3]	Yes	
10868	Other	CNTNAP3B-C20orf203, chromosomal gain	*PAX5* Whole DEL	*PAX5* DEL		[A] LOSS:chr9:73333-38773443	Yes	
10876	*DUX4*-r		*CDKN2A/B* DEL [CN=0]	*CDKN2A/B* DEL		[A] LOSS:chr9:19915005-22315516 [CN=0]	Yes	
10925	*DUX4*-r		PAR1 DEL	Chromosome X LOSS		[A] LOSS:chrX	Yes	
10927	*ETV6::RUNX1*-like		PAR1 GAIN	Chromosome X GAIN		[A] GAIN:chrX [CN=2]	Yes	
10927	*ETV6::RUNX1*-like		*ETV6* Ex2-5 DEL	*ETV6* Ex2-5 DEL	[A] DEL:chr12:11,691,000-11,827,145; DEL:	[A] LOSS:chr12:11680769-11874782 [CN=0]	Yes	
11053	*DUX4*-r						NA	
11060	Low hypodiploidy		*ETV6* GAIN	Chromosome 12 GAIN		[A] GAIN:chr12	Yes	
11060	Low hypodiploidy		*EBF1* GAIN	Chromosome 5 GAIN		[A] GAIN:chr5	Yes	
11060	Low hypodiploidy		PAR1 GAIN	Chromosome X GAIN		[A] GAIN:chrX	Yes	
11062	*BCL2/MYC*			*RB1* DEL		[A] LOSS: chr13:48177743-88198564	No	MLPA not done
11148	*DUX4*-r						NA	
11149	Other	*SPOP* abnormality	*ETV6* Whole DEL	*ETV6* DEL		[B] LOSS:chr12:100000-26800000	Yes	
11178	*DUX4*-r		*ETV6* Ex2 DEL [CN=0]	*ETV6* Ex2 DEL	[A] DEL:chr12:11,746,267-11,756,513		Yes	
11178	*DUX4*-r		*ETV6* Whole DEL	*ETV6* Whole DEL		[A] LOSS:chr12:11499537-15615794	Yes	
11440	*TCF3::PBX1*						NA	
11553	*TCF3::PBX1*		*CDKN2A/B* DEL	*CDKN2A/B* DEL		[A] LOSS:chr9:20199286-39012282	Yes	
11553	*TCF3::PBX1*		*PAX5* Whole DEL	*PAX5* DEL		[A] LOSS:chr9:20199286-39012282	Yes	
11556	*DUX4*-r		*RUNX1* Whole GAIN	Chromosome 21 GAIN		[A] GAIN:chr21	Yes	

11556	*DUX4*-r		*ERG* Ex5-11 DEL	*ERG* Ex5-11 DEL	[A] DEL:chr21:38,390,503-38,439,569	[A] LOSS:chr21:38390029-38447483	Yes	
11556	*DUX4*-r		*IKZF1* Ex4-7 DEL	*IKZF1* Ex4-7 DEL	[A] DEL:chr7:50,345,146-50,395,986	[A] LOSS:chr7:50334677-50396071	Yes	
11672	*DUX4*-r			*PAX5* Ex2-5 DEL	[A] DEL:chr9:36,985,226-37,026,621		No	Not detected by MLPA
11672	*DUX4*-r		*ERG* Ex4-7 DEL	*ERG* Ex4-7 DEL	[A] DEL:chr21:38,401,815-38,504,076		Yes	
11672	*DUX4*-r		*IKZF1* Ex4-7 DEL	*IKZF1* Ex4-7 DEL	[A] DEL:chr7:50,345,146-50,395,989		Yes	
11734	*CRLF2*-r		*ETV6* Ex1 GAIN				No	No evidence in WGS data
11734	*CRLF2*-r			*PAX5* Ex1 DEL	[A] DEL:chr9:37,025,709-37,257,823		No	Single probe CNA
11734	*CRLF2*-r		*PAR1* DEL	*P2RY8::CRLF2*	[A] DEL:chrX:1,216,133-1,536,517	[A] LOSS:chrX:1216174-1536323	Yes	
11811	*DUX4*-r			*IKZF1* Ex2-3 DEL	[A] DEL:chr21:38,390,503-38,439,569		No	Not detected by MLPA
11811	*DUX4*-r			*IKZF1* Ex4-7 DEL	[A] DEL:chr7:50,345,145-50,395,982		No	Not detected by MLPA
11811	*DUX4*-r		*CDKN2A/B* DEL	*CDKN2A/B* DEL		[B] LOSS:21400000-22100000	Yes	
11832	*PAX5* alt		*CDKN2A/B* DEL [CN=0]	*CDKN2A/B* DEL	[A] DEL:chr9:21,827,636-22,008,354	[A] LOSS:chr9:21826346-22008345 [CN=0]	Yes	
11832	*PAX5* alt		*PAX5* Ex8-10 DEL	*PAX5* Ex7-10 DEL		[A] LOSS:chr9:22008346-36961499	Yes	
11886	*PAX5* alt		*CDKN2A/B* DEL [CN=0]	*CDKN2A/B* DEL		[A] LOSS:chr9:21309656-22100169 [CN=0]	Yes	
11886	*PAX5* alt		*ETV6* Ex1 DEL	*ETV6* Ex1 DEL	[A] DEL:chr12:11,583,489-11,651,124	[A] LOSS:chr12:11582827-11651382	Yes	
11957	*DUX4*-r			*PAX5* Ex7 DEL	[A] DEL:chr9:36,890,368-36,935,397		No	Single probe CNA
11957	*DUX4*-r		*ERG* Ex5-10 DEL	*ERG* Ex5-9 DEL	[A] chr21:38,391,957-38,439,570		Yes	
11959	ABL-class			*PAX5* Ex2-6 DEL	[A] DEL:chr9:36,929,006-37,030,754		No	Not detected by MLPA
11959	ABL-class		*EBF1* Ex16 DEL	*EBF1* Ex16 DEL	[A] DEL:chr5:150,128,505-158,700,805		Yes	
12083	*DUX4*-r			Chromosome 21 GAIN		[A] GAIN:chr21	No	Not detected by MLPA
12083	*DUX4*-r			*PAX5* Ex1-7 DEL	[A] DEL:chr9:36,892,099-37,284,644		No	Not detected by MLPA
12083	*DUX4*-r		*ERG* Ex5-9 DEL	*ERG* Ex5-9 DEL	[A] DEL:chr21:38,391,960-38,439,567		Yes	
12118	*DUX4*-r			*PAX5* Ex1 DEL	[A] DEL:chr9:37,025,244-37,229,583	[A] LOSS:chr9:37025221-37231506	No	Single probe CNA
12118	*DUX4*-r		*ERG* Ex4-12 DEL	*ERG* Ex4-12 DEL	[A] DEL:chr21:38,367,542-38,504,075	[A] LOSS:chr21:38367187-38508225	Yes	
12134	*DUX4*-r		*CDKN2A/B* DEL [CN=0]	*CDKN2A/B* DEL	[A] DEL:chr9:20,343,228-22,959,502	[A] LOSS:chr9:20342809-22959189 [CN=0]	Yes	
12134	*DUX4*-r		PAR1 GAIN	Chromosome X GAIN		[A] GAIN:chrX	Yes	
12134	*DUX4*-r		*ERG* Ex5-9 DEL	*ERG* Ex5-9 DEL	[A] DEL:chr21:38,391,972-38,439,567		Yes	
12202	Near haploidy		PAR1 GAIN	Chromosome X GAIN		[A] GAIN:chrX	Yes	
12334	*DUX4*-r		*ERG* Ex5-9 DEL	*ERG* Ex5-9 DEL	[A] DEL:chr21:38,391,972-38,439,564		Yes	
12335	*DUX4*-r						NA	
12356	*DUX4*-r			*ERG* Ex5-11 DEL	[A] DEL:chr21:38,390,527-38,439,567		No	Not detected by MLPA
12356	*DUX4*-r			*ERG* Ex5-9 DEL	[A] DEL:chr21:38,391,972-38,439,569		No	Not detected by MLPA
12373	High Hyperdiploidy		*CDKN2A/B* DEL [CN=0]	*CDKN2A/B* DEL	[A] chr9:19,698,261-23,334,299	[A] LOSS:chr9:19697892-23334129	Yes	
12373	High Hyperdiploidy		PAR1 GAIN	Chromosome X GAIN		[A] GAIN:chrX	Yes	
12460	*DUX4*-r		*ETV6* Ex1 DEL				No	No evidence in WGS data
12460	*DUX4*-r			*ERG* Ex4 DEL	[A] DEL:chr21:38,426,202-38,504,076		No	Single probe CNA
12522	*PAX5* alt		*CDKN2A/B* DEL	*CDKN2A/B* DEL		[A] LOSS:chr9:21840014-28626546 [CN=0]	Yes	
12522	*PAX5* alt		*PAX5* Whole DEL	*PAX5* DEL		[A] LOSS:chr9:28983585-37421440	Yes	
12641	*TCF3::PBX1*		*CDKN2A/B* DEL	*CDKN2A/B* DEL		[A] LOSS:chr9:73181-39012257	Yes	
12641	*TCF3::PBX1*		*PAX5* Whole DEL	*PAX5* DEL		[A] LOSS:chr9:73181-39012257	Yes	
12729	*ETV6::RUNX1*-like						NA	
12816	*DUX4*-r		*ERG* Ex5-11 DEL	*ERG* Ex5-11 DEL	[A] DEL:chr21:38,390,498-38,439,569	[A] LOSS:chr21:38389149-38439475	Yes	
12820	*DUX4*-r		*ERG* Ex5-9 DEL	*ERG* Ex5-9 DEL	[A] DEL:chr21:38,391,967-38,439,567		Yes	
12910	*PAX5* alt		*CDKN2A/B* DEL [CN=0]	*CDKN2A/B* DEL		[A] LOSS:chr9:19925042-22150442 [CN=0]	Yes	
12910	*PAX5* alt		*PAX5* Ex8-10 DEL	*PAX5* Ex8-10 DEL		[A] LOSS:chr9:22151314-36951349	Yes	
12911	*MEF2D*-r			*ETV6* Ex2 DUP	[A] DUP:chr12:11,715,801-11,785,965	[A] NEUTRAL:chr12:11715898-11788236 [CN=	No	Single probe CNA
12911	*MEF2D*-r		*ETV6* Whole DEL	*ETV6* Whole DEL		[A] LOSS:chr12:9582570-11715897 [CN=1]; ch	Yes	
19578	iAMP21-ALL		*CDKN2A/B* DEL [CN=0]	*CDKN2A/B* DEL		[A] LOSS:chr9:20363485-22404207 [CN=0]	Yes	
19578	iAMP21-ALL		*ETV6* Ex2 DEL	*ETV6* Ex2 DEL	[A] DEL:chr12:11,692,504-11,781,592	[A] LOSS:chr12:11692160-11782259 [CN=1]	Yes	
19578	iAMP21-ALL		*IKZF1* Whole DEL	*IKZF1* DEL		[A] LOSS:chr7:50119677-51143462 [CN=1]	Yes	
19578	iAMP21-ALL		*PAX5* Whole DEL	*PAX5* DEL		[A] LOSS:chr9:22404208-39012290 [CN=1]	Yes	
19730	*TCF3::PBX1*						NA	
19732	*PAX5* alt			*PAX5* Ex9 DEL	[A] DEL:chr9:36,840,983-36,853,930 [CN=0]		No	Single probe CNA
19732	*PAX5* alt		*CDKN2A* DEL [CN=0]	*CDKN2A/B* DEL	[A] DEL:chr9:21,832,208-21,993,868	[A] LOSS:chr9:21831295-21993928 [CN=0]	Yes	
19732	*PAX5* alt		*CDKN2A/B* DEL	*CDKN2A/B* DEL		[A] LOSS:chr9:21993929-39838756 [CN=1]	Yes	
19732	*PAX5* alt		*IKZF1* Ex2-7 DEL	*IKZF1* Ex2-7 DEL	[A] DEL:chr7:50,345,201-50,395,934	[A] LOSS:chr7:50345270-50395862 [CN=1]	Yes	
19732	*PAX5* alt		*PAX5* Whole DEL	*PAX5* DEL		[A] LOSS:chr9:21993929-39838756 [CN=1]	Yes	
19827	*DUX4*-r		*ERG* Ex5-11 DEL	*ERG* Ex5-11 DEL	[A] DEL:chr21:38,389,542-38,439,583	[A] LOSS:chr21:38389031-38439605 [CN=1]	Yes	
20035	*DUX4*-r		*ERG* Ex5-9 DEL	*ERG* Ex5-9 DEL	[A] DEL:chr21:38,391,967-38,439,569		Yes	
20035	*DUX4*-r		*IKZF1* Ex2-7 DEL	*IKZF1* Ex2-7 DEL	[A] DEL:chr7:50,307,745-50,395,981	[A] LOSS:chr7:50306339-50396150 [CN=1]	Yes	
20161	*PAX5* alt		*CDKN2A/B* DEL [CN=0]	*CDKN2A/B* DEL	[A] DEL:chr9:19,615,707-22,304,976	[A] LOSS:chr9:19615569-22305408 [CN=0]	Yes	
20315	*TCF3::PBX1*						NA	

20317	*ZEB2/CEBP*		*CDKN2A/B* DEL [CN=0]	*CDKN2A/B* DEL	[A] DEL:chr9:21827687-22008307, chr9:21,966,498-22,008,361		Yes	
20323	*ZNF384*-r		*ETV6* Ex2-8 DEL	*ETV6* Ex2-8 DEL	[A] DEL:chr12:11,650,554-12,026,253	[A] LOSS:chr12:11650257-12026452 [CN=1]	Yes	
20447	*ETV6::RUNX1*			*ETV6* Ex1-5 DEL		[A] LOSS:chr12:79069-11870468 [CN=1]	No	MLPA not done
20447	*ETV6::RUNX1*			*RB1* Ex19-27 DEL	[A] DEL:chr13:48,305,460-48,602,651	[A] LOSS:chr13:48304482-48602575 [CN=1]	No	MLPA not done
20515	*DUX4*-r			*PAX5* Ex7 DEL	[A] DEL:chr9:36,904,013-36,947,069		No	Single probe CNA
20628	ABL-class			*RB1* Ex18-27 DEL	[A] DEL:chr13:48,410,378-48,500,981		No	Not detected by MLPA
20628	ABL-class		*BTG1* Ex2 DEL	*BTG1* Ex2 DEL		[A] LOSS:chr12:91882758-92142207 [CN=0]	Yes	
20628	ABL-class		*CDKN2A/B* DEL [CN=0]	*CDKN2A/B* DEL	[A] DEL:chr9:21,827,622-22,008,353	[A] LOSS:chr9:21827355-22008453 [CN=0]	Yes	
20628	ABL-class		*IKZF1* Ex2-3 DEL	*IKZF1* Ex2-3 DEL	[A] DEL:chr7:50,305,871-50,361,368	[A] LOSS:chr7:50305426-50361567 [CN=1]	Yes	
20628	ABL-class		*PAX5* Whole DEL	*PAX5* DEL		[A] LOSS:chr9:22008454-39011893 [CN=1]	Yes	
20653	*ZNF384*-r		*CDKN2A/B* DEL [CN=0]	*CDKN2A/B* DEL		[A] LOSS:chr9:21195942-22075473 [CN=0]	Yes	
20683	*DUX4*-r			*ERG* Ex5-9 DEL	[A] DEL:chr21:38,391,967-38,439,565		No	Not detected by MLPA
20683	*DUX4*-r			*ERG* Ex1 DEL	[A] DEL:chr21:38,494,811-38,504,063		No	Single probe CNA
20696	*DUX4*-r		*CDKN2A/B* DEL	*CDKN2A/B* DEL	[A] DEL:chr9:21,917,176-22,107,478	[A] LOSS:chr9:21917017-22107893 [CN=1]	Yes	
20706	*PAX5*alt		*CDKN2A/B* DEL	*CDKN2A/B* DEL		[A] LOSS:chr9:21952924-22218768 [CN=0]	Yes	
20706	*PAX5*alt		*PAX5* Whole DEL	*PAX5* DEL		[A] LOSS:chr9:27812750-38773965 [CN=1]	Yes	
20716	*DUX4*-r			*PAX5* Ex1 DEL	[A] DEL:chr9:37,022,576-37,284,644	[A] LOSS:chr9:37022449-37294383 [CN=1]	No	Single probe CNA
20720	*DUX4*-r		*ERG* Ex5-9 DEL	*ERG* Ex5-9 DEL	[A] DEL:chr21:38,391,965-38,439,573		Yes	
20724	*DUX4*-r		*ERG* Ex4-12 DEL	*ERG* Ex4-12 DEL	[A] DEL:chr21:38,377,143-38,498,777	[A] LOSS:chr21:38376589-38499220 [CN=1]	Yes	
20755	*ETV6::RUNX1*		*ETV6* Whole DEL	*ETV6* Ex2-8 DEL	[A] DEL:chr12:11,650,552-12,034,354	[A] LOSS:chr12:11639902-11877450 [CN=1],ch	Yes	
20756	*PAX5* P80R			*CDKN2A/B* DEL		[A] LOSS:chr9:21151178-22452147 [CN=0]	No	MLPA not done
20756	*PAX5* P80R			*PAX5* DEL		[A] LOSS:chr9:22452729-39017438 [CN=1]	No	MLPA not done
20764	*ETV6::RUNX1*-like			*ETV6* Ex1 DEL	[A] DEL:chr12:11,645,719-11,667,777		No	Not detected by MLPA
20764	*ETV6::RUNX1*-like			*PAX5* Ex2-6 DEL	[A] DEL:chr9:36,928,983-37,030,741		No	Not detected by MLPA
20764	*ETV6::RUNX1*-like		*ETV6* Ex2-5 DEL [CN=0]	*ETV6* Ex2-5 DEL	[A] DEL:chr12:11,707,929-11,880,177; DEL:	[A] LOSS:chr12:11,688,009-11,870,748 [CN=1]	Yes	
20874	*PAX5* P80R		*CDKN2A* DEL [CN=0]	*CDKN2A* DEL	[A] DEL:chr9:21,967,312-21,970,385		Yes	
20874	*PAX5* P80R		*CDKN2A/B* DEL	*CDKN2A/B* DEL		[A] LOSS:chr9:73244-30197286 [CN=1]	Yes	
20874	*PAX5* P80R		*PAX5* Whole DEL	*PAX5* DEL		[A] LOSS:chr9:30505066-39012257 [CN=1]	Yes	
20951	*ETV6::RUNX1*		*PAX5* Ex1-6 DEL	*PAX5* Ex1-6 DEL	[A] DEL:chr9:36,929,001-37,187,544	[A] LOSS:chr9:36928439-37187725 [CN=1]	Yes	
21190	*ETV6::RUNX1*			*PAX5* Ex9-10 DEL		[A] LOSS:chr9:32059690-36860938 [CN=1]	No	Single probe CNA
21190	*ETV6::RUNX1*		*BTG1* Ex2 DEL	*BTG1* Ex2 DEL	[A] DEL:chr12:91,884,358-92,144,345	[A] LOSS:chr12:91884168-92141734 [CN=0]	Yes	
21190	*ETV6::RUNX1*		*CDKN2A/B* DEL [CN=0]	*CDKN2A/B* DEL	[A] DEL:chr9:21,929,048-22,008,353	[A] LOSS: chr9:21667353-23664870 [CN=0]	Yes	
21190	*ETV6::RUNX1*		*ETV6* Whole DEL	*ETV6* DEL		[A] LOSS:chr12:9023629-14824771 [CN=1]	Yes	
21198	*ETV6::RUNX1*-like		*ETV6* Ex2-3 [CN=0], Ex5-8 DE	*ETV6* DEL	[A] DEL:chr12:11,710,849-11,977,201	[A] LOSS:chr12:11710191-11866386 [CN=0], c	Yes	
21198	*ETV6::RUNX1*-like		*ETV6* Ex2-3 DEL [CN=0]	*ETV6* Ex2-4 DEL		[A] LOSS:chr12:11710191-11866386 [CN=0]	Yes	
21198	*ETV6::RUNX1*-like		*ETV6* Ex5-8 DEL	*ETV6* Ex5-8 DEL		[A] LOSS:chr12:11,866,387-11,977,127 [CN=1]	Yes	
21230	*DUX4*-r		*ERG* Ex5-12 DEL	*ERG* Ex5-12 DEL	[A] DEL:chr21:38,382,224-38,439,567	[A] LOSS:chr21:38381808-38439379 [CN=1]	Yes	
21230	*DUX4*-r		*IKZF1* Ex4-7 DEL	*IKZF1* Ex4-7 DEL	[A] DEL:chr7:50,345,147-50,395,983	[A] LOSS:chr7:50338543-50396076 [CN=1]	Yes	
21318	*KMT2A*-r						NA	
21321	*ETV6::RUNX1*-like	*CRLF2*-r		*CDKN2A/B* DEL	[A] DEL:chr9:21,973,578-22,008,352		No	Not detected by MLPA
21321	*ETV6::RUNX1*-like	*CRLF2*-r		*IKZF1* Ex1-3 DEL	[A] DEL:chr7:35,699,305-50,342,361		No	Not detected by MLPA
21321	*ETV6::RUNX1*-like	*CRLF2*-r	*ETV6* Ex1-3 DEL	*ETV6* Ex1 DEL	[A] DEL:chr12:11,580,572-11,814,534	[A] LOSS:chr12:11580419-11850141 [CN=1]	Yes	
21321	*ETV6::RUNX1*-like	*CRLF2*-r	PAR1 DEL	*P2RY8::CRLF2*	[A] INV:chrX:1,231,926-1,467,109		Yes	
21321	*ETV6::RUNX1*-like	*CRLF2*-r	*PAX5* Ex2-10 DEL	*PAX5* Ex2-10 DEL	[A] DEL:chr9:36,785,169-37,030,752	[A] LOSS:chr9:36,784,859-37,030,530 [CN=1]	Yes	
21322	*DUX4*-r						NA	
21342	*CRLF2*-r			*CDKN2A/B* DEL	[A] DEL:chr9:21,973,585-22,008,357		No	Not detected by MLPA
21342	*CRLF2*-r			*ETV6* Ex6-8 DEL	[A] DEL:chr12:11,883,813-11,917,087		No	Single probe CNA
21342	*CRLF2*-r		PAR1 DEL	*P2RY8::CRLF2*	[A] DEL:chrX:1,216,121-1,536,524		Yes	
21424	Other	*MAPK10*-r					NA	
21437	*DUX4*-r			*ERG* Ex1 DEL	[A] DEL:chr21:38,474,417-38,504,072		No	Single probe CNA
21466	*ETV6::RUNX1*-like	Ph-like	*CDKN2A/B* DEL	*CDKN2A/B* DEL		[A] LOSS:chr9:20221477-24564523 [CN=0]	Yes	
21466	*ETV6::RUNX1*-like	Ph-like	*IKZF1* DEL	*IKZF1* Whole DEL		[A] LOSS:chr7:27169556-51653187 [CN=1]	Yes	
21466	*ETV6::RUNX1*-like	Ph-like	*PAX5* DEL	*PAX5* Whole DEL		[B] LOSS:chr9:24564524-39838758 [CN=1]	Yes	
21487	*IKZF1* N159Y	*PAX5*alt		*IKZF1* Ex5 DUP	[A] DUP:chr7:50,382,257-50,382,810		No	Single probe CNA
21487	*IKZF1* N159Y	*PAX5*alt		*ETV6* Ex6-8 DEL	[A] DEL:chr12:11,877,202-11,895,300		No	Single probe CNA
21532	*NUTM1*-r		*BTG1* GAIN				No	No evidence in WGS data
21532	*NUTM1*-r		*EBF1* GAIN				No	No evidence in WGS data
21532	*NUTM1*-r		*ETV6* GAIN				No	No evidence in WGS data
21532	*NUTM1*-r		*CDKN2A/B* DEL [CN=0]	*CDKN2A/B* DEL		[A] LOSS:chr9:21,889,312-22,051,695 [CN=0]	Yes	
21532	*NUTM1*-r		*PAX5* DEL	*PAX5* Whole DEL		[A] LOSS:chrr9:36334125-39839079 [CN=1]	Yes	
21568	*DUX4*-r			*ERG* Ex5-11 DEL	[A] DEL:chr21:38,389,976-38,439,567		No	MLPA not done
21568	*DUX4*-r		*IKZF1* Ex4-7 DEL	*IKZF1* Ex4-7 DEL	[A] DEL:chr7:50,345,140-50,395,989	[A] LOSS:chr7:50344354-50396070 [CN=1]	Yes	

21689	*DUX4*-r						NA	
21693	*PAX5*alt		*CDKN2A/B* DEL [CN=0]	*CDKN2A/B* DEL		[A] LOSS:chr9:20102121-22151781 [CN=0]	Yes	
21693	*PAX5*alt		*ETV6* Whole DEL	*ETV6* DEL		[A] LOSS:chr12:11533713-12436497 [CN=1]	Yes	
21693	*PAX5*alt		*PAX5* Whole DEL	*PAX5* DEL		[A] LOSS:chr9:22151782-38773616 [CN=1]	Yes	
21694	*IKZF1* N159Y			*IKZF1* Ex5 DUP	[A] DUP:chr7:50,381,660-50,386,282		No	MLPA not done
21708	Near haploidy		PAR1 GAIN [CN=4]	Chromosome X CNN-LOH		[A] CNN-LOH:chrX [CN=2]	Yes	
21795	iAMP21-ALL						NA	
21800	*PAX5*alt		*CDKN2A/B* DEL [CN=0]	*CDKN2A/B* DEL	[A] DEL:chr9:21,902,129-22,008,356	[A] LOSS:chr9:21901759-22008138 [CN=0]	Yes	
21800	*PAX5*alt		*ETV6* DEL	*ETV6* DEL		[A] LOSS:chr12:9582497-24804550 [CN=1]	Yes	
21800	*PAX5*alt		*IKZF1* DEL	*IKZF1* DEL		[A] LOSS:chr7:49022416-56811719 [CN=1]	Yes	
21800	*PAX5*alt		*PAX5* Ex8-10 DEL	*PAX5* Ex7-10 DEL		[A] LOSS:chr9:22008562-36927696 [CN=1]	Yes	
21869	*CRLF2*-r		*CDKN2A/B* DEL	*CDKN2A/B* DEL	[A] DEL:chr9:21,971,756-22,008,355		Yes	
21869	*CRLF2*-r		*ETV6* Ex1 DEL	*ETV6* Ex1 DEL	[A] DEL:chr12:11,584,191-11,651,130		Yes	
21869	*CRLF2*-r		PAR1 DEL	*P2RY8::CRLF2*	[A] DEL:chrX:1,216,131-1,536,517		Yes	
21869	*CRLF2*-r		*PAX5* Ex2-8 DEL	*PAX5* Ex2-8 DEL	[A] DEL:chr9:36,877,439-37,030,748		Yes	
21940	*KMT2A*-r		*CDKN2A/B* DEL [CN=0]	*CDKN2A/B* DEL		[A] LOSS:chr9:21475234-22895118 [CN=1]	Yes	
22006	*DUX4*-r			*CDKN2A/B* DEL	[A] DEL:chr9:19,948,668-36,946,866		No	Not detected by MLPA
22006	*DUX4*-r			*ERG* Ex5-12 DEL	[A] DEL:chr21:38,382,226-38,439,567		No	Not detected by MLPA
22006	*DUX4*-r			*PAX5* DEL	[A] DEL:chr9:19,948,668-36,946,866		No	Not detected by MLPA
22006	*DUX4*-r			*PAX5* Ex1 DEL	[A] DEL:chr9:37,027,598-37,229,588		No	Single probe CNA
22037	*DUX4*-r		*ERG* Ex5-11 DEL	*ERG* Ex5-11 DEL	[A] DEL:chr21:38,389,561-38,439,569	[A] LOSS:chr21:38388921-38439403 [CN=1]	Yes	
22045	*DUX4*-r						NA	
22046	*ZEB2/CEBP*			*PAX5* Ex7-8 DEL	[A] DEL:chr9:36,907,847-36,950,788, chr9:36,886,888-36,928,223, chr9:36,857,586-36,953,		No	Single probe CNA
22046	*ZEB2/CEBP*		*RB1* Whole DEL	*RB1* DEL		[A] LOSS:chr13:30461569-114351080 [CN=1]	Yes	
22065	*DUX4*-r		*CDKN2B* DEL	*CDKN2A/B* DEL	[A] DEL:chr9:21,975,757-22,009,697		Yes	
22094	*ZNF384*-r		*ETV6* Whole DEL	*ETV6* Ex1-2 DEL	[A] DEL:chr12:11,595,099-11,799,330	[A] LOSS:chr12:11,594,736-11,806,508 [CN=1]	Yes	
22104	*JAK2*-r						NA	
22150	*PAX5* P80R		*CDKN2A/B* DEL [CN=0]	*CDKN2A/B* DEL		[A] LOSS:chr9:21800361-22229232 [CN=0]	Yes	
22150	*PAX5* P80R		*IKZF1* Whole DEL	*IKZF1* DEL		[A] LOSS:chr7:40468-58119312 [CN=1]	Yes	
22150	*PAX5* P80R		*PAX5* Whole DEL	*PAX5* DEL		[A] LOSS:chr9:22229233-39047953 [CN=1]	Yes	
22153	*PAX5*alt		*CDKN2A/B* DEL	*CDKN2A/B* DEL		[A] LOSS:chr9:21787170-22117399 [CN=0]	Yes	
22188	Other	*CNTNAP3B* abnormality, *MAPKAP1*-r					NA	
22224	*DUX4*-r		*ETV6* Whole DEL	*ETV6* Whole DEL		[A] LOSS:chr12:11216648-12718281 [CN=1]	Yes	
22245	*TCF3::PBX1*						NA	
22327	High Hyperdiploidy						NA	
22340	iAMP21-ALL		*BTG1* Ex2 DEL	*BTG1* Ex2 DEL	[A] DEL:chr12:91,886,500-92,144,338	[A] LOSS:chr12:91886472-92143941 [CN=1]	Yes	
22346	*DUX4*-r			*ERG* Ex6 DEL	[A] DEL:chr21:38,403,200-38,405,200		No	Single probe CNA
22354	*DUX4*-r		*CDKN2A* DEL	*CDKN2A/B* DEL		[A] LOSS:chr9:21696692-22296201 [CN=1]	Yes	
22354	*DUX4*-r		*CDKN2B* DEL [CN=0]	*CDKN2A/B* DEL	[A] DEL:chr9:21,975,757-22,009,697		Yes	
22355	*DUX4*-r	*ZEB2/CEBP*					NA	
22367	*ETV6::RUNX1*-like						NA	
22368	*KMT2A*-r		*CDKN2A/B* DEL				No	No evidence in WGS data
22368	*KMT2A*-r		PAR1 GAIN				No	No evidence in WGS data
22387	*DUX4*-r			*PAX5* Ex1 DEL	[A] DEL:chr9:37,026,415-37,257,823	[A] LOSS:chr9:37024729-37257830 [CN=1]	No	Single probe CNA
22387	*DUX4*-r		*CDKN2A/B* DEL [CN=0]	*CDKN2A/B* DEL		[A] LOSS:chr9:21195912-24939118 [CN=0]	Yes	
22387	*DUX4*-r		*ERG* Ex5-9 DEL	*ERG* Ex5-9 DEL	[A] DEL:chr21:38,391,971-38,439,566	[A] LOSS:chr21:38388681-38439739 [CN=1]	Yes	
22405	*DUX4*-r		*ERG* Ex5-9 DEL	*ERG* Ex5-9 DEL	[A] DEL:chr21:38,391,890-38,439,573	[A] LOSS:chr21:38391591-38441903 [CN=1]	Yes	
22416	High Hyperdiploidy		*PAX5* Ex2-10 DEL				No	No evidence in WGS data
22416	High Hyperdiploidy		*CDKN2A/B* DEL	*CDKN2A/B* DEL		[A[LOSS:chr9:21502262-24783443 [CN=1]	Yes	
22417	*DUX4*-r		*CDKN2A/B* DEL	*CDKN2A/B* DEL	[A] DEL:chr9:20,725,875-22,395,845		Yes	
22417	*DUX4*-r		*ERG* Ex5-9 DEL	*ERG* Ex5-9 DEL	[A] DEL:chr21:38,391,947-38,439,569		Yes	
22417	*DUX4*-r		*IKZF1* Ex4-7 DEL	*IKZF1* Ex4-7 DEL	[A] DEL:chr7:50,345,146-50,395,985	[A] LOSS:chr7:50331493-50395974 [CN=1]	Yes	
22418	*JAK2*-r		PAR1 LOSS	Chromosome X LOSS		[A] LOSS:chrX:251202-13565086, chrX:658701	Yes	
22418	*JAK2*-r		*IKZF1* Whole DEL	*IKZF1* DEL		[A] LOSS:chr7:49852196-54235297 [CN=1]	Yes	
22466	*ETV6::RUNX1*-like		*ETV6* Whole DEL	*ETV6* Whole DEL	[A] DEL:chr12:11,666,778-11,731,179; DEL:	[A] LOSS:chr12:79055-11666308 [CN=1]; LOS	Yes	
22466	*ETV6::RUNX1*-like		*IKZF1* Whole DEL	*IKZF1* DEL		[A] LOSS:chr7:37234706-50527437 [CN=1]	Yes	
22466	*ETV6::RUNX1*-like		*RB1* Whole DEL	*RB1* DEL		[A] LOSS:chr13 [CN=1]	Yes	
22572	*IGH::IL3*			*IKZF1* Ex2-7 DEL	[A] DEL:chr7:50,307,741-50,395,988		No	Not detected by MLPA
22572	*IGH::IL3*			*RB1* Ex18-27 DEL	[A] DEL:chr13:48,410,367-48,503,062	[A] LOSS:chr13:48409802-48505511 [CN=1]	No	Not detected by MLPA
22572	*IGH::IL3*			*PAX5* Ex1 DEL	[A] DEL:chr9:37,024,811-37,257,837		No	Single probe CNA
22572	*IGH::IL3*		*CDKN2A/B* DEL	*CDKN2A/B* DEL	[A] DEL:chr9:21,973,586-22,008,357		Yes	
22584	*PAX5*alt		*CDKN2A/B* DEL [CN=0]	*CDKN2A/B* DEL	[A] DEL:chr9:21,973,586-22,008,358		Yes	

22584	*PAX5* alt		*PAX5* Ex6-10 DEL	*PAX5* Ex6-10 DEL		[A] LOSS:chr9:112861-36986757 [CN=1]	Yes	
22621	*ZNF384*-r		*ETV6* Whole DEL	*ETV6* DEL		[A] LOSS:chr12:11,582,736-13,210,364 [CN=1]	Yes	
22660	ABL-class			*EBF1* Ex2-10	[A] DEL:chr5:158,757,490-159,097,158		No	Single probe CNA
22660	ABL-class		*BTG1* Ex2 DEL	*BTG1* Ex2 DEL	[A] DEL:chr12:91,884,362-92,144,347	[A] LOSS:chr12:91883860-92144200 [CN=1]	Yes	
22660	ABL-class		*CDKN2A/B* DEL [CN=0]	*CDKN2A/B* DEL	[A] DEL:chr9:21,827,633-22,008,354	[A] LOSS:chr9:21875434-22007973 [CN=0]	Yes	
22660	ABL-class		*IKZF1* Ex2-7 DEL	*IKZF1* Ex2-7 DEL	[A] DEL:chr7:50,307,742-50,395,983	[A] LOSS:chr7:50307140-50395862 [CN=1]	Yes	
22689	*TCF3::PBX1*						NA	
22716	High Hyperdiploidy						NA	
22722	*BCR::ABL1*		*IKZF1* Ex2-7 DEL	*IKZF1* Ex2-7 DEL		[A] LOSS:chr7:50307605-50396085 [CN=1]	Yes	
22722	*BCR::ABL1*		*PAX5* Ex1-6 DEL	*PAX5* Ex1 DEL	[A] DEL:chr9:37,026,414-37,257,835		Yes	
22722	*BCR::ABL1*		*PAX5* Ex1-6 DEL	*PAX5* Ex1-7 DEL	[A] DEL:chr9:36,897,444-37,284,634		Yes	
22804	*DUX4*-r			*ERG* Ex5-11 DEL	[A] DEL:chr21:38,389,967-38,439,565	[A] LOSS;chr21:38389479-38448573 [CN=1]	No	MLPA not done
22804	*DUX4*-r			*PAX5* Ex1 DEL	[A] DEL:chr9:37,021,660-37,287,982		No	MLPA not done
22804	*DUX4*-r			*PAX5* Ex1-5 DEL	[A] DEL:chr9:36,990,624-37,241,030	[A] LOSS:chr9:36990485-37240861 [CN=0]	No	MLPA not done
22897	*DUX4*-r			*ERG* Ex5-11 DEL	[A] DEL:chr21:38,389,560-38,439,565		No	Not detected by MLPA
22918	*DUX4*-r		*CDKN2A/B* DEL	*CDKN2A/B* DEL		[A] LOSS:chr9:73009-38691989 [CN=1]	Yes	
22918	*DUX4*-r		*PAX5* Whole DEL	*PAX5* DEL		[A] LOSS:chr9:73009-38691989 [CN=1]	Yes	
22922	*BCL2/MYC*						NA	
22964	*PAX5* alt		*CDKN2A/B* DEL [CN=0]	*CDKN2A/B* DEL		[A] LOSS:chr9:21166020-23295828 [CN=0]	Yes	
22964	*PAX5* alt		*IKZF1* Ex4-7 DEL	*IKZF1* Ex4-7 DEL	[A] DEL:chr7:50,345,145-50,395,985		Yes	
22972	*PAX5* alt			*CDKN2A/B* DEL		[A] LOSS:chr9:20366936-25928943 [CN=0]	No	Not detected by MLPA
22972	*PAX5* alt			*PAX5* Ex6-8 DEL	[A] DEL:chr9:36,877,822-36,990,496	[A] LOSS:chr9:36878358-36990568 [CN=0]	No	Not detected by MLPA
22980	Other						NA	
23003	*KMT2A*-r		*IKZF1* Ex2-7 DEL	*IKZF1* Ex2-7 DEL	[A] DEL:chr7:50,307,744-50,395,986	[A] LOSS:chr7:50306627-50396119 [CN=1]	Yes	
23063	*PAX5* alt		*CDKN2A/B* DEL	*CDKN2A/B* DEL		[A] LOSS:chr9:21052662-22338831 [CN=1]	Yes	
23074	*DUX4*-r			*ERG* Ex6-7 DEL	[A] DEL:chr21:38,402,149-38,405,213		No	MLPA not done
23074	*DUX4*-r		*ETV6* Whole DEL	*ETV6* DEL	[A] DEL:chr12:11,650,692-11,917,090; DEL:	[A] LOSS:chr12:11623006-11917289 [CN=1]	Yes	
23075	*HLF*-r		*BTG1* Ex2 DEL [CN=0]	*BTG1* Ex2 DEL	[A] DEL:chr12:91,884,359-92,144,338; DEL:	[A] LOSS:chr12:91884116-92144291 [CN=0]	Yes	
23075	*HLF*-r		*PAX5* Ex2-10 DEL	*PAX5* Ex2-8 DEL		[A] LOSS:chr9:36877153-37030638 [CN=1]	Yes	
23078	*DUX4*-r		*CDKN2A* DEL	*CDKN2A/B* DEL	[A] DEL:chr9:19,985,425-21,978,950	[A] LOSS:chr9:19984904-21981968 [CN=1]	Yes	
23078	*DUX4*-r		*ERG* Ex4-7 DEL	*ERG* Ex4-7 DEL	[A] DEL:chr21:38,401,808-38,493,945	[A] LOSS:chr21:38401180-38493898 [CN=1]	Yes	
23078	*DUX4*-r		*IKZF1* Ex4-7 DEL	*IKZF1* Ex4-7 DEL	[A] DEL:chr7:50,345,149-50,395,983	[A] LOSS:chr7:50344276-50395862 [CN=1]	Yes	
23081	*PAX5* alt			*IKZF1* Ex3 DEL	[A] DEL:chr7:50,326,720-50,329,156		No	Single probe CNA
23081	*PAX5* alt		*CDKN2A* DEL [CN=0]	*CDKN2A* DEL	[A] DEL:chr9:21,890,736-21,991,592	[A] LOSS:chr9:21888745-21991662 [CN=1]	Yes	
23081	*PAX5* alt		*CDKN2A/B* DEL	*CDKN2A/B* DEL		[A] LOSS:chr9:21991663-36961971 [CN=1]	Yes	
23081	*PAX5* alt		*PAX5* Ex1-6 GAIN	*PAX5* Ex1-6 GAIN		[A] GAIN:chr9:36961972-39011869 [CN=3]	Yes	
23081	*PAX5* alt		PAX5 Ex8-10 DEL	*PAX5* Ex7-10 DEL		[A] LOSS:chr9:21991663-36961971 [CN=1]	Yes	
23110	High Hyperdiploidy		PAR1 GAIN	Chromosome X GAIN		[A] GAIN:chrX [CN=3]	Yes	
23114	*DUX4*-r		*CDKN2B* DEL	*CDKN2A/B* DEL	[A] DEL:chr9:21,975,729-22,009,706		Yes	
23114	*DUX4*-r		*ERG* Ex4-12 DEL	*ERG* Ex4-12 DEL	[A] DEL:chr21:38,367,534-38,504,098	[A] LOSS:chr21:38366984-38508261 [CN=1]	Yes	
23114	*DUX4*-r		*IKZF1* Ex2-3 DEL	*IKZF1* Ex2-3 DEL	[A] DEL:chr7:50,306,352-50,345,251		Yes	
23132	*DUX4*-r	*PAX5* alt		*PAX5* Ex4-5 DUP	[A] DUP:chr9:37,002,158-37,007,199		No	Single probe CNA
23132	*DUX4*-r	*PAX5* alt	*CDKN2A/B* DEL	*CDKN2A/B* DEL	[A] chr9:21,902,123-22,008,353	[A] LOSS:chr9:21901356-22008935 [CN=1]	Yes	
23176	*PAX5* alt		*CDKN2A/B* DEL	*CDKN2A/B* DEL		[A] LOSS:chr9:9645843-39047846 [CN=1]	Yes	
23176	*PAX5* alt		*ETV6* Whole DEL	*ETV6* DEL		[A] LOSS:chr12:11542662-15052836 [CN=1]	Yes	
23176	*PAX5* alt		*IKZF1* Whole DEL	*IKZF1* DEL		[A] LOSS:chr7:38335223-54921257 [CN=1]	Yes	
23176	*PAX5* alt		*PAX5* Wole DEL	*PAX5* DEL		[A] LOSS:chr9:9645843-39047846 [CN=1]	Yes	
23176	*PAX5* alt		*RB1* Whole DEL	*RB1* DEL		[A] LOSS:chr13:39674438-114350591 [CN=1]	Yes	
23273	*PAX5* alt			*PAX5* Ex1-6 GAIN		[A] GAIN:chr9:36947205-37120760 [CN=4]	No	Not detected by MLPA
23273	*PAX5* alt			*PAX5* Ex7-10 DEL		[A] LOSS:chr9:22033006-36947204 [CN=1]	No	Not detected by MLPA
23273	*PAX5* alt		*CDKN2A/B* DEL	*CDKN2A/B* DEL		[A] LOSS:chr9:21423152-22033005 [CN=0]	Yes	
23321	*ETV6::RUNX1*-like			*ETV6* DEL		[A] LOSS:chr12:10675736-27722131 [CN=1]	No	MLPA not done
23321	*ETV6::RUNX1*-like			*IKZF1* DEL		[A] LOSS:chr7:36441606-55142869 [CN=1]	No	MLPA not done
23321	*ETV6::RUNX1*-like			*PAX5* Ex2-6 DEL	[A] DEL:chr9:36,928,998-37,026,627		No	MLPA not done
23333	*PAX5* alt			*CDKN2A* DEL	[A] DEL:chr9:21,903,599-21,993,857	[A] LOSS:chr9:21902095-21993912 [CN=1]	No	MLPA not done
23333	*PAX5* alt			*CDKN2A/B* DEL		[A] LOSS:chr9:21993913-26265199 [CN=1]	No	MLPA not done
23333	*PAX5* alt			*IKZF1* Ex4-7 DEL	[A] DEL:chr7:50,345,145-50,395,981	[A] LOSS:chr7:50339028-50395774 [CN=1]	No	MLPA not done
23333	*PAX5* alt			*PAX5* Ex6-10 DEL		[A] LOSS:chr9:26428262-36994073 [CN=1]	No	MLPA not done
23343	*TCF3::PBX1*						NA	
23353	*JAK2*-r		*ETV6* Ex5-8 DEL	*ETV6* Ex5-8 DEL		[A] LOSS:chr12:11,860,692-12,038,879 [CN=1]	Yes	
23353	*JAK2*-r		*IKZF1* Whole DEL	*IKZF1* DEL		[A] LOSS:chr7:50083593-51907825 [CN=1]	Yes	
23383	iAMP21-ALL						NA	

23445	*DUX4*-r		*IKZF1* Ex4-7 DEL	*IKZF1* Ex4-7 DEL	[A] DEL:chr7:50342899-50395932 (IKZF1 Ex	[A] LOSS:chr7:50338940-50396179 [CN=1]	Yes	
23481	*ZNF384*-r		*IKZF1* Ex4-8 DEL	*IKZF1* Ex4-8 DEL	[A] DEL:chr7:50,345,148-50,416,804	[A] LOSS:chr7:50337783-50416620 [CN=1]	Yes	
23507	*DUX4*-r		*IKZF1* Ex4-7 DEL	*IKZF1* Ex4-7 DEL	[A] DEL:chr7:50,345,146-50,395,983	[A] LOSS:chr7:50344470-50397891 [CN=1]	Yes	
23515	*ETV6::RUNX1*-like		*IKZF1* Whole DEL	*ETV6* DEL		[A] LOSS:chr12:9283374-19934499 [CN=1]	Yes	
23515	*ETV6::RUNX1*-like		*ETV6* Whole DEL	*IKZF1* DEL		[A] LOSS:chr7:38362849-53547121 [CN=1]	Yes	
23533	*DUX4*-r			Chromosome 12 GAIN		[A] GAIN:chr12 [CN=3]	No	Not detected by MLPA
23533	*DUX4*-r		*CDKN2A/B* DEL	*CDKN2A/B* DEL	[A] DEL:chr9:21,830,337-22,450,349		Yes	
23560	*PAX5*alt			*IKZF1* Ex1 DEL	[A] DEL:chr7:50,275,640-50,306,466		No	Single probe CNA
23560	*PAX5*alt		*BTG1* Ex2	*BTG1* Ex2 DEL	[A] DEL:chr12:92,081,217-92,144,345	[A] LOSS:chr12:92081118-92144420 [CN=1]	Yes	
23560	*PAX5*alt		*CDKN2A/B* DEL [CN=0]	*CDKN2A/B* DEL		[A] LOSS:chr9:21,866,844-22,213,228 [CN=0]	Yes	
23560	*PAX5*alt		*IKZF1* Ex5-8 DEL	*IKZF1* Ex5-8 DEL		[A] LOSS:chr7:50,380,366-50,448,911 [CN=1]	Yes	
23650	*PAX5*alt		*CDKN2A/B* DEL [CN=0]	*CDKN2A/B* DEL	[A] DEL:chr9:21,969,000-22,008,352	[A] LOSS:chr9:21,969,000-22,008,352 [CN=0]	Yes	
23650	*PAX5*alt		*PAX5* Whole DEL	*PAX5* DEL		[A] LOSS:chr9:112753-40846154 [CN=1]	Yes	
23673	*ETV6::RUNX1*-like			*PAX5* Ex1 DEL	[A] DEL:chr9:37,026,410-37,257,822		No	Single probe CNA
23673	*ETV6::RUNX1*-like		*ETV6* Whole DEL [CN=0]	*ETV6* DEL		[A] LOSS:chr12:9056507-12333719 [CN=0]	Yes	
23673	*ETV6::RUNX1*-like		*IKZF1* Whole DEL	*IKZF1* DEL		[A] LOSS:chr7:50,202,596-50,404,624 [CN=1]	Yes	
23678	Other	*TCF3*-r, chromosomal gain					NA	
23769	*DUX4*-r			*ERG* Ex1 DEL	[A] DEL:chr21:38,447,146-38,504,074	[A] LOSS:chr21:38438834-38503935 [CN=1]	No	Single probe CNA
23813	*ZEB2/CEBP*		*CDKN2A/B* DEL [CN=0]	*CDKN2A/B* DEL		[A] LOSS:chr9:21241959-22241498 [CN=0]	Yes	
23813	*ZEB2/CEBP*		PAR1 GAIN	Chromosome X GAIN		[A] GAIN:chrX [CN=2]	Yes	
23842	*DUX4*-r		*CDKN2A* Ex5 DEL [CN=0]	*CDKN2A* DEL	[A] DEL:chr9:21,955,973-21,969,175		Yes	
23842	*DUX4*-r		*CDKN2A/B* Ex2 DEL	*CDKN2A/B* DEL	[A] DEL:chr9:19,703,999-39,659,310 (inc. CI	[A] LOSS:chr9:19703664-39047952 [CN=1]	Yes	
23842	*DUX4*-r		*IKZF1* Ex4-7 DEL	*IKZF1* Ex4-7 INV	[A] INV:chr7:50,364,342-50,394,442		Yes	
23842	*DUX4*-r		*PAX5* Whole DEL	*PAX5* DEL		[A] LOSS:chr9:19703664-39047952 [CN=1]	Yes	
23863	*DUX4*-r		*IKZF1* Ex4-7 DEL	*IKZF1* Ex4-7 DEL	[A] DEL:chr7:50,345,146-50,395,985	[A] LOSS:chr7:50344346-50397882 [CN=1]	Yes	
23881	*PAX5*alt		*CDKN2A/B* DEL [CN=0]	*CDKN2A/B* DEL		[A] LOSS:chr9:20078553-22326860 [CN=0]	Yes	
23881	*PAX5*alt		*PAX5* Ex2-5 GAIN [CN=5]	*PAX5* Ex2-5 INV	[A] INV:chr9:36,995,339-37,026,089 [Elevated copy number based on read cont (149/108)]		Yes	
23913	*PAX5*alt			*CDKN2A/B* DEL		[A] LOSS:chr9:21309260-22198830 [CN=0]	No	MLPA not done
23913	*PAX5*alt			*PAX5* DEL		[A] LOSS:chr9:22198831-39011843 [CN=1]	No	MLPA not done
23919	*CRLF2*-r		*IKZF1* Ex4-7 DEL	*IKZF1* Ex4-7 DEL	[A] DEL:chr7:50,345,145-50,395,984	[A] LOSS:chr7:50344508-50396006 [CN=1]	Yes	
23919	*CRLF2*-r		PAR1 DEL	*P2RY8::CRLF2*	[A] DEL:chrX:1,216,129-1,536,517	[A] LOSS:chrX:1215972-1536700 [CN=1]	Yes	
23919	*CRLF2*-r		*PAX5* Ex1-5 DEL	*PAX5* Ex1-5 DEL	[A] DEL:chr9:36,975,668-37,282,012	[A] LOSS:chr9:36975203-37288141 [CN=1]	Yes	
23922	*ETV6::RUNX1*		PAR1 GAIN	Chromosome X GAIN		[A] GAIN:chrX [CN=3]	Yes	
23922	*ETV6::RUNX1*		*ETV6* Whole DEL	*ETV6* DEL		[A] LOSS:chr12:9582647-22544850 [CN=1]	Yes	
23936	*PAX5*alt		*CDKN2A* Ex5 DEL [CN=0]	*CDKN2A* DEL	[A] DEL:chr9:21,889,404-21,971,504	[A] LOSS:chr9:21888765-21971131 [CN=0]	Yes	
23936	*PAX5*alt		*CDKN2A/B* Ex2 DEL	*CDKN2A/B* DEL		[A] LOSS:chr9:21971647-37240345 [CN=1]	Yes	
23936	*PAX5*alt		*IKZF1* Ex4-7 DEL	*IKZF1* Ex4-7 DEL	[A] DEL:chr7:50,345,146-50,395,982	[A] LOSS:chr7:50344390-50395998 [CN=1]	Yes	
23936	*PAX5*alt		*PAX5* Whole DEL	*PAX5* DEL		[A] LOSS:chr9:21971647-37240345 [CN=1]	Yes	
24041	*PAX5*alt			*IKZF1* DEL		[A] LOSS:chr7:38317121-58119281 [CN=1]	No	Not detected by MLPA
24041	*PAX5*alt			*PAX5* Ex7-10 DEL		[A] LOSS:chr9:22,547,578-36,952,722 [CN=1]	No	Not detected by MLPA
24041	*PAX5*alt		*CDKN2A/B* DEL	*CDKN2A/B* DEL		[A] LOSS:chr9:21,834,596-22,547,577 [CN=0]	Yes	
24079	*MEF2D*-r		*CDKN2A/B* DEL	*CDKN2A/B* DEL		[B] LOSS:chr9:20300000-30400000 [CN=1]	Yes	
24079	*MEF2D*-r		*ETV6* Whole DEL	*ETV6* Whole DEL		[A] LOSS:chr12:9582601-26632277	Yes	
24079	*MEF2D*-r		*RB1* Whole DEL	*RB1* Whole DEL		[A] LOSS:chr13:40566071-114350290	Yes	
24082	*ZNF384*-r			Chromosome X LOSS		[A] LOSS:chrX	No	Not detected by MLPA
24082	*ZNF384*-r		*CDKN2B* DEL	*CDKN2A/B* DEL	[A] DEL:chr9:21,994,153-22,008,357		Yes	
24100	*PAX5*alt		*CDKN2A/B* DEL [CN=0]	*CDKN2A/B* DEL	[A] DEL:chr9:21,672,948-22,451,246	[A] LOSS:chr9:21672736-22164610 [CN=0]	Yes	
24100	*PAX5*alt		*EBF1* Whole GAIN	Chromosome 5 GAIN		[A] GAIN:chr5	Yes	
24232	*ETV6::RUNX1*-like		*BTG1* Ex2 DEL	*BTG1* Ex2 DEL	[A] DEL:chr12:91,884,356-92,141,778	[A] LOSS:chr12:91883896-92141825 [CN=0]	Yes	
24232	*ETV6::RUNX1*-like		*ETV6* DEL	*ETV6* DEL	[A] DEL:chr12:9,327,671-11,760,408	[A] LOSS:chr12:9585093-31111142	Yes	
24232	*ETV6::RUNX1*-like		*IKZF1* DEL	*IKZF1* DEL		[A] LOSS:chr7:45136141-52637623	Yes	
24232	*ETV6::RUNX1*-like		*PAX5* Ex1-7 DEL	*PAX5* Ex1-7 DEL	[A] DEL:chr9:36,886,409-37,363,894	[A] LOSS:chr9:36,885,940-37,363,656	Yes	
24262	*PAX5*alt						NA	
24284	Low hypodiploidy			Chromosome X GAIN			No	MLPA not done
24284	Low hypodiploidy			*RB1* Ex9-13 DEL	[A] DEL:chr13:48,363,027-48,379,251		No	MLPA not done
24332	*PAX5*alt		*CDKN2A/B* DEL [CN=0]	*CDKN2A/B* DEL		[A] LOSS:chr9:21242944-22293118 [CN=0]	Yes	
24338	*IGH::ID4*		*CDKN2A/B* DEL [CN=0]	*CDKN2A/B* DEL		[A] LOSS:chr9:20266811-22552034 [CN=0]	Yes	
24338	*IGH::ID4*		*PAX5* Whole DEL	*PAX5* DEL		[A] LOSS:chr9:22552035-38774062	Yes	
24371	High Hyperdiploidy						NA	
24390	High Hyperdiploidy	*CRLF2*-r		*P2RY8::CRLF2*	[A] DEL:chrX:1,216,134-1,536,649		No	Not detected by MLPA
24391	*DUX4*-r			*ERG* Ex5-8 DEL	[A] DEL:chr21:38,397,634-38,498,321		No	MLPA not done
24419	*ZEB2/CEBP*			*RB1* DEL		[A] LOSS:chr13:30419802-114351080	No	MLPA not done

24423	*DUX4*-r		*ETV6* Whole GAIN	Chromosome 12 GAIN		[A] GAIN: chr12	Yes	
24456	ZNF384-r						NA	
24493	*MEF2D*-r			*RB1* Whole DEL		[A] LOSS:chr13:22537111-67228537	No	MLPA not done
24553	*ETV6::RUNX1*			*BTG1* Ex2 DEL	[A] DEL:chr12:91,491,350-92,144,353	[A] LOSS:chr12:91491194-92144010	No	MLPA not done
24553	*ETV6::RUNX1*			*ETV6* DEL		[A] LOSS:chr12:20882-21752053	No	MLPA not done
24553	*ETV6::RUNX1*			*IKZF1* Ex2-3 DEL	[A] DEL:chr7:50,305,856-50,333,012		No	MLPA not done
24553	*ETV6::RUNX1*			*PAX5* Ex2-6 DEL	[A] DEL:chr9:36,929,001-37,026,628	[A] LOSS:chr9:36928505-37027189	No	MLPA not done
24566	*ZNF384*-r		*CDKN2A/B* DEL	*CDKN2A/B* DEL		[B] LOSS:chr9:21800000-22500000	Yes	
24566	*ZNF384*-r		*ETV6* DEL	*ETV6* DEL	[A] DEL:chr12:11,883,807-11,902,819	[A] LOSS:chr12:11638117-11973374	Yes	
24566	*ZNF384*-r		*RB1* Ex14-26 DEL	*RB1* Ex7-27 DEL	[A] DEL:chr13:48,410,377-48,500,864, chr13	[A] LOSS:chr13:48,357,890-48,500,847	Yes	
24601	*ZNF384*-r						NA	
24607	*ZNF384*-r						NA	
24669	Other	*NOTCH2* deletion					NA	
24680	*CRLF2*-r			*PAX5* Ex7 DEL	[A] DEL:chr9:36,899,620-36,948,832		No	Single probe CNA
24680	*CRLF2*-r		PAR1 GAIN	Chromosome X GAIN		[A] GAIN:chrX	Yes	
24680	*CRLF2*-r		PAR1 DEL	*P2RY8::CRLF2*	[A] DEL:chrX:1,216,134-1,536,649	[A] LOSS:chrX:1,216,273-1,536,945	Yes	
24848	Other	*SH2B3* abnormality	*IKZF1* Ex4-8 DEL	*IKZF1* Ex4-8 DEL	[A] DEL:chr7:50,345,142-50,416,806	[A] LOSS:chr7:50344414-50416572	Yes	
25379	*KMT2A*-r		*CDKN2A/B* DEL [CN=0]	*CDKN2A/B* DEL		[A] LOSS:chr9:21935452-22216044 [CN=0]	Yes	
25647	Near haploidy		PAR1 GAIN [CN=4]	Chromosome X GAIN		[A] GAIN:chrX [CN=4]	Yes	
26681	ABL-class			*CDKN2A/B* DEL		[A] LOSS:chr9:19978634-22263378	No	Not detected by MLPA
26681	ABL-class		*IKZF1* Ex4-7 DEL	*IKZF1* Ex4-7 DEL	[D] DEL:chr7:50,345,141-50,395,989		Yes	
26681	ABL-class		*PAX5* Ex2-5 DEL	*PAX5* Ex2-5 DEL	[D] DEL:chr9:36,988,954-37,030,751	[D] LOSS:chr9:36988398-37003251	Yes	
26681	ABL-class		*RB1* Ex19-26 DEL	*RB1* Ex19-27 DEL	[A] DEL:chr13:48,410,378-48,500,865	[A] LOSS:chr13:48409520-48500937 [CN=0]	Yes	
26772	*KMT2A*-r						NA	
27162	High Hyperdiploidy		*CDKN2A/B* GAIN				No	No evidence in WGS data
27162	High Hyperdiploidy		PAR1 GAIN	Chromosome X GAIN		[A] GAIN:chrX [CN=2]	Yes	
27304	*TCF3::PBX1*			*PAX5* Ex6 DEL	[A] DEL:chr9:36,959,388-36,967,333		No	Single probe CNA
27354	*TCF3::PBX1*						NA	
27397	*TCF3::PBX1*		*CDKN2A/B* DEL	*CDKN2A/B* DEL		[A] LOSS:chr9:73163-38773461	Yes	
27397	*TCF3::PBX1*		*PAX5* Whole DEL	*PAX5* DEL		[A] LOSS:chr9:73163-38773461	Yes	
27442	ABL-class		*CDKN2A/B* DEL [CN=0]	*CDKN2A/B* DEL	[D] DEL:chr9:21,981,147-25,406,936	[A] LOSS:chr9:21158610-22207572 [CN=0]	Yes	
27442	ABL-class		*ETV6* Whole DEL	*ETV6* DEL		[A] LOSS:chr12:20569-32887710	Yes	
27442	ABL-class		*IKZF1* Ex4-7 DEL	IKZF1 Ex4-7 DEL	[A] DEL:chr7:50,345,147-50,395,989	[A] LOSS:chr7:50344474-50396110	Yes	
27442	ABL-class		*PAX5* Whole DEL	*PAX5* DEL		[A] LOSS:chr9:22207573-39012032	Yes	
27809	High Hyperdiploidy			ETV6 Ex2-4 DEL	[A] DEL:chr12:11,750,432-11,868,434		No	Not detected by MLPA
27809	High Hyperdiploidy		*EBF1* GAIN	Chromosome 5 GAIN		[A] GAIN:chr5	Yes	
27809	High Hyperdiploidy		PAR1 GAIN	Chromosome X GAIN		[A] GAIN:chrX	Yes	
28593	High Hyperdiploidy			Chromosome X GAIN		[A] GAIN:chrX	No	Not detected by MLPA
28627	High Hyperdiploidy			Chromosome X GAIN		[A] GAIN:chrX	No	MLPA not done
28956	*BCR::ABL1*			*CDKN2A/B* DEL	[A] DEL:chr9:21,975,740-22,004,991		No	Not detected by MLPA
28956	*BCR::ABL1*		PAR1 GAIN	Chromosome X GAIN		[A] GAIN:chrX	Yes	
28956	*BCR::ABL1*		*IKZF1* Ex1,6,8 GAIN	*IKZF1* Ex2-3 DEL	[A] DEL:chr7:50,307,734-50,366,385		Yes	
28956	*BCR::ABL1*		*IKZF1* Ex1,6,8 GAIN	*IKZF1* Ex2-7 DEL	[A] DEL:chr7:50,307,739-50,395,992		Yes	
28957	High Hyperdiploidy		*CDKN2A/B* DEL [CN=0]	*CDKN2A/B* DEL	[A] DEL:chr9:21,974,793-22,778,765	[A[] LOSS:chr9:21974295-22732867 [CN=0]	Yes	
28959	High Hyperdiploidy		PAR1 GAIN	Chromosome X GAIN		[A] GAIN:chrX [CN=4]	Yes	
28982	ABL-class		*IKZF1* Ex4-8 DEL	*IKZF1* Ex4-8 DEL	[D] DEL:chr7:50,345,143-50,416,249	[A] LOSS:chr7:50344565-50416486	Yes	
29079	High Hyperdiploidy		*BTG1* GAIN	Chromosome 12 GAIN		[A] GAIN:chr12	Yes	
29079	High Hyperdiploidy		*ETV6* GAIN	Chromosome 12 GAIN		[A] GAIN:chr12	Yes	
29079	High Hyperdiploidy		PAR1 GAIN	Chromosome X GAIN		[A] GAIN:chrX [CN=2]	Yes	

References

1. Moorman AV, Enshaei A, Schwab C, et al. A novel integrated cytogenetic and genomic classification refines risk stratification in pediatric acute lymphoblastic leukemia. Blood. 2014;124(9):1434-1444.

Supplementary Table 9: UKALL-CNA risk group status of patients. UKALL-CNA risk grouping was available for 137 patients. Patients were classified as good-risk (0), intermediate-risk (1) or poor-risk (2) based on the copy number of eight key genes/regions by MLPA, as previously described.[1] Abbreviation: NA = not applicable. Ph-like definition in this table indicates abnormalities that drive the unique GEP of the subtype (Ph-like) that is defined by WTS.

Cohort	Patient	Unique ID	WGS subgroup	Other WGS subgroups	UKALL-CNA risk group[1]
1	1	28956	*BCR::ABL1*	High Hyperdiploidy	NA
1	2	22722	*BCR::ABL1*		NA
1	3	10194	*BCR::ABL1*		NA
1	4	26681	ABL-class		NA
1	5	27442	ABL-class		NA
1	6	28982	ABL-class		NA
1	7	21190	*ETV6::RUNX1*		NA
1	8	20447	*ETV6::RUNX1*		NA
1	9	24553	*ETV6::RUNX1*		NA
1	10	20755	*ETV6::RUNX1*		NA
1	11	25647	Near haploidy		NA
1	12	12202	Near haploidy		NA
1	13	27162	High Hyperdiploidy		NA
1	14	27809	High Hyperdiploidy		NA
1	15	28593	High Hyperdiploidy		NA
1	16	28957	High Hyperdiploidy		NA
1	17	28959	High Hyperdiploidy		NA
1	18	29079	High Hyperdiploidy		NA
1	19	28627	High Hyperdiploidy		NA
1	20	10740	High Hyperdiploidy		NA
1	21	23110	High Hyperdiploidy		NA
1	22	24284	Low hypodiploidy		NA
1	23	22340	iAMP21-ALL		NA
1	24	19578	iAMP21-ALL		NA
1	25	20724	*DUX4*-r	iAMP21-ALL	0
1	26	24390	High Hyperdiploidy	*CRLF2*-r	NA
1	27	20951	*ETV6::RUNX1*		NA
1	28	26772	*KMT2A*-r		NA
1	29	21318	*KMT2A*-r		NA
1	30	21940	*KMT2A*-r		NA
1	31	23003	*KMT2A*-r		NA
1	32	22368	*KMT2A*-r		NA
1	33	25379	*KMT2A*-r		NA
1	34	21708	Near haploidy		NA
1	35	7653	Near haploidy		NA
1	36	27354	*TCF3::PBX1*		NA
1	37	27397	*TCF3::PBX1*		NA
1	38	27304	*TCF3::PBX1*		NA
2	39	8451	*MEF2D*-r		1
2	40	8878	*ZNF384*-r		0
2	41	9051	*IKZF1* N159Y		0
2	42	9469	*DUX4*-r		2
2	43	9850	ABL-class	*MEF2D*-r	2
2	44	10062	*PAX5* alt		2
2	45	10184	*ZNF384*-r		0
2	46	10186	*DUX4*-r		0
2	47	10310	*DUX4*-r		0
2	48	10442	iAMP21-ALL		NA
2	49	10656	*PAX*5alt		2
2	50	10868	Other	*CNTNAP3B* abnormality, chromosomal gain	2
2	51	10876	*DUX4*-r		1
2	52	10925	*DUX4*-r		0
2	53	10927	*ETV6::RUNX1*-like		0
2	54	11053	*DUX4*-r		0
2	55	11060	Low hypodiploidy		NA
2	56	11062	*BCL2/MYC*		NA
2	57	11148	DUX4-r		0
2	58	11149	Other	*SPOP* abnormality	0
2	59	11178	*DUX4*-r		0
2	60	11440	*TCF3::PBX1*		NA
2	61	11553	*TCF3::PBX1*		NA
2	62	11556	*DUX4*-r		2
2	63	11672	*DUX4*-r		2
2	64	11734	*CRLF2*-r		NA
2	65	11811	*DUX4*-r		1
2	66	11832	*PAX5* alt		2
2	67	11886	*PAX5* alt		0
2	68	11957	*DUX4*-r		0
2	69	11959	ABL-class		0
2	70	12083	*DUX4*-r		0
2	71	12118	*DUX4*-r		0
2	72	12134	*DUX4*-r		1
2	73	12334	*DUX4*-r		0
2	74	12335	*DUX4*-r		0

2	75	12356	*DUX4*-r	iAMP21-ALL	0
2	76	12373	High Hyperdiploidy		NA
2	77	12460	*DUX4*-r		0
2	78	12522	*PAX5* alt		2
2	79	12641	*TCF3::PBX1*		NA
2	80	12729	*ETV6::RUNX1*-like		0
2	81	12816	*DUX4*-r		0
2	82	12820	*DUX4*-r		NA
2	83	12908	*PAX5* P80R		2
2	84	12910	*PAX5* alt		2
2	85	12911	*MEF2D*-r		0
2	86	19730	*TCF3::PBX1*		NA
2	87	19732	*PAX5* alt		2
2	88	19827	*DUX4*-r		NA
2	89	20035	*DUX4*-r		2
2	90	20161	*PAX5* alt		1
2	91	20315	*TCF3::PBX1*	*PAX5* alt	NA
2	92	20317	*ZEB2/CEBP*		1
2	93	20323	*ZNF384*-r		0
2	94	20515	*DUX4*-r		0
2	95	20628	ABL-class		1
2	96	20653	*ZNF384*-r		1
2	97	20683	*DUX4*-r		0
2	98	20696	*DUX4*-r		1
2	99	20706	*PAX5* alt		2
2	100	20716	*DUX4*-r		1
2	101	20720	*DUX4*-r		0
2	102	20756	*PAX5* P80R		2
2	103	20764	*ETV6::RUNX1*-like		0
2	104	20874	*PAX5* P80R		2
2	105	21198	*ETV6::RUNX1*-like		0
2	106	21230	*DUX4*-r		2
2	107	21321	*ETV6::RUNX1*-like	*CRLF2*-r	1
2	108	21322	*DUX4*-r		0
2	109	21342	*CRLF2*-r		NA
2	110	21424	Other	*MAPK10*-r	0
2	111	21437	*DUX4*-r		0
2	112	21466	*ETV6::RUNX1*-like	Ph-like	2
2	113	21487	*IKZF1* N159Y	*PAX5* alt	0
2	114	21532	*NUTM1*-r		2
2	115	21568	*DUX4*-r		2
2	116	21689	*DUX4*-r		0
2	117	21693	*PAX5* alt		1
2	118	21694	*IKZF1* N159Y		0
2	119	21795	iAMP21-ALL		NA
2	120	21800	*PAX5* alt		1
2	121	21869	*CRLF2*-r		NA
2	122	22006	*DUX4*-r		0
2	123	22037	*DUX4*-r		0
2	124	22045	*DUX4*-r		0
2	125	22046	*ZEB2/CEBP*		NA
2	126	22065	*DUX4*-r		1
2	127	22094	*ZNF384*-r		0
2	128	22104	*JAK2*- r		0
2	129	22150	*PAX5* P80R		2
2	130	22153	*PAX5* alt		1
2	131	22188	Other	*CNTNAP3B* abnormality, *MAPKAP1*-r	0
2	132	22224	*DUX4*-r		0
2	133	22245	*TCF3::PBX1*		NA
2	134	22327	High Hyperdiploidy		NA
2	135	22346	*DUX4*-r		0
2	136	22352	*PAX5* P80R		2
2	137	22354	*DUX4*-r		1
2	138	22355	*DUX4*-r	*ZEB2/CEBP*	0
2	139	22367	*ETV6::RUNX1*- like		0
2	140	22387	*DUX4*-r		1
2	141	22405	*DUX4*-r		0
2	142	22416	High Hyperdiploidy		NA
2	143	22417	*DUX4*-r		2
2	144	22418	*JAK2*-r		2
2	145	22466	*ETV6::RUNX1*-like		1
2	146	22572	*IGH::IL3*		1
2	147	22584	*PAX5* alt		2
2	148	22621	*ZNF384*-r		0
2	149	22660	ABL-class		1
2	150	22689	*TCF3::PBX1*		NA
2	151	22716	High Hyperdiploidy		NA
2	152	22804	*DUX4*-r		NA
2	153	22897	*DUX4*-r		0
2	154	22918	*DUX4*-r		2

2	155	22922	*BCL2/MYC*		NA
2	156	22964	*PAX5* alt		2
2	157	22972	*PAX5* alt		2
2	158	22980	Other		0
2	159	23063	*PAX5* alt		1
2	160	23074	*DUX4* -r		0
2	161	23075	*HLF* -r		NA
2	162	23078	*DUX4* -r		2
2	163	23081	*PAX5* alt		2
2	164	23114	*DUX4* -r		2
2	165	23132	*DUX4* -r	*PAX5* alt	1
2	166	23176	*PAX5* alt		1
2	167	23273	*PAX5* alt		1
2	168	23321	*ETV6::RUNX1* -like		NA
2	169	23333	*PAX5* alt		NA
2	170	23343	*TCF3::PBX1*		NA
2	171	23353	*JAK2* -r		1
2	172	23383	iAMP21-ALL		NA
2	173	23445	*DUX4* -r		2
2	174	23481	*ZNF384* -r		2
2	175	23507	*DUX4* -r		2
2	176	23515	*ETV6::RUNX1* -like		1
2	177	23533	*DUX4* -r		1
2	178	23560	*PAX5* alt		1
2	179	23650	*PAX5* alt		1
2	180	23673	*ETV6::RUNX1* -like		1
2	181	23678	Other	*TCF3*- r, chromosomal gain	0
2	182	23769	*DUX4* -r		0
2	183	23813	*ZEB2/CEBP*		1
2	184	23842	*DUX4* -r		2
2	185	23863	*DUX4* -r		2
2	186	23881	*PAX5* alt		2
2	187	23913	*PAX5* alt		NA
2	188	23919	*CRLF2* -r		1
2	189	23922	*ETV6::RUNX1*		NA
2	190	23936	*PAX5* alt		2
2	191	24041	*PAX5* alt		1
2	192	24079	*MEF2D* -r		1
2	193	24082	*ZNF384* -r		1
2	194	24100	*PAX5* alt		1
2	195	24232	*ETV6::RUNX1* -like		1
2	196	24262	*PAX5* alt		NA
2	197	24332	*PAX5* alt		1
2	198	24338	*IGH::ID4*		2
2	199	24371	High Hyperdiploidy		NA
2	200	24391	*DUX4* -r		0
2	201	24419	*ZEB2/CEBP*		NA
2	202	24423	*DUX4* -r		0
2	203	24456	*ZNF384* -r		0
2	204	24493	*MEF2D* -r		NA
2	205	24566	*ZNF384* -r		1
2	206	24601	*ZNF384* -r		0
2	207	24607	*ZNF384* -r		0
2	208	24669	Other	*NOTCH2* deletion	0
2	209	24680	*CRLF2* -r		NA
2	210	24848	Other	*SH2B3* abnormality	NA

References

1. Moorman AV, Enshaei A, Schwab C, et al. A novel integrated cytogenetic and genomic classification refines risk stratification in pediatric acute lymphoblastic leukemia. Blood. 2014;124(9):1434-1444.
